# Digital Mental Health Interventions for Adolescents in Low- and Middle-Income Countries: Scoping Review

**DOI:** 10.2196/51376

**Published:** 2024-10-29

**Authors:** Carolina Wani, Lisa McCann, Marilyn Lennon, Caterina Radu

**Affiliations:** 1 Department of Computer and Information Sciences University of Strathclyde Glasgow United Kingdom

**Keywords:** adolescents, mental health, low- and middle-income countries, LMICs, digital mental health interventions, DMHIs, cultural appropriateness, implementation, design, evaluation, mobile phone

## Abstract

**Background:**

Digital mental health interventions (DMHIs) are increasingly recognized as potential solutions for adolescent mental health, particularly in low- and middle-income countries (LMICs). The United Nations’ Sustainable Development Goals and universal health coverage are instrumental tools for achieving mental health for all. Within this context, understanding the design, evaluation, as well as the barriers and facilitators impacting adolescent engagement with mental health care through DMHIs is essential.

**Objective:**

This scoping review aims to provide insights into the current landscape of DMHIs for adolescents in LMICs.

**Methods:**

The Joanna Briggs Institute scoping review methodology was used, following the recommendations of the PRISMA-ScR (Preferred Reporting Items for Systematic Reviews and Meta-Analyses Extension for Scoping Reviews). Our search strategy incorporated 3 key concepts: population "adolescents," concept "digital mental health interventions," and context "LMICs." We adapted this strategy for various databases, including ACM Digital Library, APA PsycINFO, Cochrane Library, Google Scholar (including gray literature), IEEE Xplore, ProQuest, PubMed (NLM), ScienceDirect, Scopus, and Web of Science. The articles were screened against a specific eligibility criterion from January 2019 to March 2024.

**Results:**

We analyzed 20 papers focusing on DMHIs for various mental health conditions among adolescents, such as depression, well-being, anxiety, stigma, self-harm, and suicide ideation. These interventions were delivered in diverse formats, including group delivery and self-guided interventions, with support from mental health professionals or involving lay professionals. The study designs and evaluation encompassed a range of methodologies, including randomized controlled trials, mixed methods studies, and feasibility studies.

**Conclusions:**

While there have been notable advancements in DMHIs for adolescents in LMICs, the research base remains limited. Significant knowledge gaps persist regarding the long-term clinical benefits, the maturity and readiness of LMIC digital infrastructure, cultural appropriateness, and cost-effectiveness across the heterogeneous LMIC settings. Addressing these gaps necessitates large-scale, co-designed, and culturally sensitive DMHI trials. Future work should address this.

## Introduction

### Background

The World Health Organization (WHO) defines mental health as “a state of mental well-being that enables people to cope with the stresses of life, to realize their abilities, to learn well and work well, and to contribute to their communities. Mental health is an integral component of health and well-being and is more than the absence of mental disorder” [1]. The United Nations General Assembly has underscored the key role of universal health coverage (UHC)—where everyone can access the health services they need without financial hardship—in achieving health for all [1]. Indeed, UHC is central to the health-related Sustainable Development Goals (SDGs), namely target 3.4: “by 2030, reduce by one-third premature mortality from noncommunicable diseases through prevention and treatment and promote mental health and well-being” [1]. While these global agendas are instruments for achieving mental health for all, there is international recognition that adolescent mental health is a distinct and critical public health and human rights issue [2-4].

In 2020, the WHO–UNICEF (United Nations International Children’s Emergency Fund)–Lancet Commission called for a renewed focus on the SDGs for advancing child and adolescent health, including mental health [5]. The Commission made a powerful argument that children and adolescents should be at the center of the SDGs to protect their human rights. In addition, this was an ethical and economic investment [5]. Despite this, the *World Mental Health Report* states that approximately 90 WHO member states—less than half of all member states—had a mental health policy or plan specifically for children and adolescents [1]. The report emphasizes the importance of national mental health plans that are fully compliant with human rights instruments, sufficiently resourced, and regularly monitored and evaluated [1].

The onset of mental health disorders typically occurs during adolescence (between the ages of 10-19 years), an important time for developing social and emotional skills [6,7]. It is here where adolescents form coping strategies that enable mental health. Equally, it is a time when young people become vulnerable to risk-taking behaviors, for example, substance abuse. Indeed, suicide is the leading cause of adolescent death [6]. Worldwide, 13% of the world’s adolescents (aged 10-19 years) live with a mental disorder [8]; however, widening treatment gaps mean many conditions remain undiagnosed and untreated [6]. Moreover, mental health conditions disproportionately affect adolescents in low- and middle-income countries (LMICs) [1]. LMIC economies are those in which the 2022 gross national income per capita was less than US $13,845 [9]. Approximately 90% of the world’s 1.2 billion adolescents reside in LMICs [8]. This population is more vulnerable to human rights violations, and where limited mental health services are available, stigma, discrimination, and social, cultural, and economic challenges are major barriers to treatment access [6,8,10].

The COVID-19 pandemic has worsened the significant burden on this population [8,11,12]. Before the pandemic, researchers explored the feasibility and clinical benefits of digital health technologies for adolescent mental health and care in LMICs [13-15]. Indeed, COVID-19 has driven the adoption of digital health care platforms in LMICs, including digital interventions for health [8,16]. Digital health interventions (DHIs) are increasingly accepted tools for the support of adolescent mental health [17-19].

The WHO defines DHIs as a discrete functionality of digital technology that is applied to achieve health objectives [20]. In this regard, digital mental health interventions (DMHIs) can be understood as a discrete functionality of digital technology that is applied to achieve mental health objectives. DHIs are classified into 4 overarching groups based on the primary user, for instance, clients (service users), health care providers (health service delivery), health system managers (administration and oversight of public health systems), and data services (data collection, management, use, and exchange) [20]. For clients, DHIs/DMHIs can be delivered at an individual or population level; in high and low-income settings; and via several devices, for example, mobile apps, websites, wearables, and smart devices. Such interventions may be self-guided [21] or delivered with lay support, for instance, from teachers [22] or trained psychologists [23]. Furthermore, some DMHIs include theories of behavior change, such as the self-determination theory [24] or the Social Cognitive Theory [23]. Indeed, research suggests that interventions grounded in behavioral theory are more likely to be effective [25,26].

### Current Barriers to Existing Mental Health Services in LMICs

Presently, there are significant and systemic barriers that limit adolescent access to mental health services, for example, cost, time, geographic location [27], stigma [28,29], lack of community knowledge and education on mental health [30], and low mental health literacy [31]. Mental health systems in LMICs lack trained mental health specialists, leading to significantly understaffed services [30,32,33]. These shortages are unequal, with urban areas benefiting from more health specialists and resources than rural areas [34,35]. Consequently, this is a potential barrier to treatment because the physical distance may place many services out of reach, thereby reinforcing the rural-urban digital divide [36]. The term digital divide can be understood as “the gap between people who do and do not have access to forms of information and communication technology” [37]. The concept is complex, going beyond the connected versus the unconnected. It is a global phenomenon but even more pronounced in LMIC settings, leading to further social, cultural, and economic inequalities [38,39].

### Key Challenges With Adolescent DMHIs in LMICs

Adolescents in LMICs have a distinct disadvantage to digital infrastructure, namely the internet [38]. In 2019, the rural-urban gap in mobile internet use across LMICs was 37%; however, LMICs in sub-Saharan Africa had the widest rural-urban gap, with those living in rural areas being 60% less likely to access the internet than those residing in urban areas [40]. Another report found gaps in literacy and related skills had the greatest impact on mobile internet use [41]. Moreover, the GSM (Global System for Mobile) Communications reports that mobile phone affordability and lack of digital skills were significant barriers in LMICs [42].

There are issues with DMHI implementation, posing a barrier to scalability. Many DMHIs, for example, require additional guidance for adolescent adherence and engagement, such as psychologists, therapists, or lay teacher support [43,44]. This may undermine the scalability of the interventions due to the shortage of health professionals for those roles [44]. Other challenges relate to their feasibility [45], political nature [46], cultural appropriateness [47], design, deployment, evaluation [48], sustainability [49], cost-effectiveness [50], privacy and data security [51], digital maturity, and readiness [52-54]. Despite this, there is evidence that DMHIs could transform adolescent mental health in low-resource settings [50,55-57].

### Rationale and Review Question

We selected the review period 2019-2024 for 3 distinct reasons; first, in 2020, the WHO launched its first guidance on designing DHIs with and for young people, recognizing the significance of youth-centered DHIs and considering their specific needs [58]. Second, that same year, the WHO–UNICEF–Lancet Commission called for a renewed focus on the SDGs for advancing child and adolescent health, including mental health, prioritizing young people in the urgent call to action [5]. Finally, the COVID-19 pandemic has profoundly impacted adolescent mental health worldwide, leading to an increased interest and investment in the delivery of quality, person-centered, remote mental health care [59]. The post–COVID-19 era has provided an opportunity to explore which DMHIs exist for adolescents in LMICs, how they are designed and evaluated, how adolescents are involved in design activities, which theories or models were consulted, and which factors affect adolescent engagement. Examining the current state of adolescent DMHIs in the context of these recent developments allows for a timely and up-to-date insight into gaps in policy, practice, and research. This scoping review aims to collate information on adolescent DMHIs in LIMC settings. The review asks “What is known about DMHIs for adolescents in LMICs, as reported in the literature?”

### Subreview Questions

The subreview questions (subRQs) were as follows:

- SubRQ1: How are adolescent DMHIs designed and evaluated within LMICs?
- SubRQ2: What are the reported activities and approaches involving adolescents in designing DMHIs, and what are the perceived outcomes of such involvement?
- SubRQ3: Which frameworks, toolkits, models, or theories were consulted or applied to the DMHI? (including implementation activities related to digital health readiness and preparedness)
- SubRQ4: What factors facilitate or hinder adolescent engagement in DMHIs in LMICs?

## Methods

### Overview

This scoping review followed the Joanna Briggs Institute Scoping Review Methodology [60] and is reported based on the PRISMA-ScR (Preferred Reporting Items for Systematic Reviews and Meta-Analyses Extension for Scoping Reviews) guidelines [61]. The PRISMA-ScR checklist is shown in Multimedia Appendix 1. Scoping reviews are ideal for disciplines with emerging evidence, for example, DMHIs for adolescents in LMICs, where the limited availability of randomized controlled trials (RCTs) hinders researchers from performing systematic reviews and assessing the quality of evidence [62]. Consistent with the purpose of scoping studies, this review does not seek to assess or appraise the quality and robustness of the evidence, nor does it generalize the findings [61,63,64]. By systematically mapping the available literature, a scoping review can highlight the current state of knowledge, identify research gaps, and potentially reveal patterns or trends that can inform future studies [60]. Previous researchers have used scoping reviews to map available evidence in a similar field [65-67].

### Eligibility Criteria

The Joanna Briggs Institute recommends the use of the PCC mnemonic, that is, population, concept, and context, to develop the inclusion criteria [60]. The population consists of adolescents aged 10-19 years, which is consistent with the WHO definition [6]. The concept under study is DMHIs, understood as a discrete functionality of digital technology that is applied to achieve mental health objectives [20]. The context of interest is LMIC economies, defined by the World Bank as nations with a gross national income per capita less than US $13,845 [9], see Textbox 1.

**Eligibility criteria based on the participant, concept, and context framework for Joanna Briggs Institute scoping reviews.**

**Inclusion criteria**

1. Population: Adolescents (World Health Organization [WHO] definition [6]—persons between the ages of 10 and 19 years).
2. Concept: Digital mental health interventions (DMHIs) [20] that are delivered to support the mental health and well-being of adolescents in low- and middle-income countries (LMICs). DMHIs focus on emotional, behavioral, or eating disorders, psychosis, suicide, self-harm, and risk-taking behaviors. Including mental health promotion and prevention interventions and those for early detection and treatment. DMHIs include mobile apps, web applications, smart devices, telehealth, SMS text messaging, and internet-based interventions.
3. Context: Low- and middle-income settings [9].
4. Sources: Published, peer-reviewed, or gray literature of any research study design (eg, randomized controlled trials, systematic reviews, case studies, qualitative, quantitative, or mixed methods, theses or dissertations, white papers, guidelines, conference proceedings, charity reports, or posters).
5. Period: Published between January 2019 and March 2024.
6. Language: Studies published in English.

**Exclusion criteria**

1. Population: Participants that do not meet the WHO definition of adolescents, that is, are aged younger than 10 years or older than 19 years.
2. Concept: DMHIs that target or only explore the concerns and views of adolescents’ parents, caregivers, guardians, teachers, and clinicians. Studies that do not focus on digital mental health or well-being.
3. Context: Not based in low- and middle-income settings.
4. Sources: No full text is available or the paper is not retrievable.
5. Period: Published before January 2019.
6. Language: Not available in English.

### Information Sources

The information sources were the following electronic databases: (1) ACM Digital Library, (2) APA PsycINFO, (3) Cochrane Library, (4) Google Scholar (gray literature included, Multimedia Appendix 2), (5) IEEE Xplore, (6) ProQuest, (7) PubMed (NLM), (8) ScienceDirect, (9) Scopus, and (10) Web of Science.

These databases were selected because they collectively align with this review’s scope on DHIs and technologies, that is, behavioral science, mental health (psychiatry and psychology), and technology.

### Search Strategy

The database identification and search strategy were developed with guidance from a faculty librarian at the University of Strathclyde. The search terms represented the primary concepts of the objectives in the review. These included a range of keywords, free text, and medical subject headings terms and combinations of the Boolean operators. The Joanna Briggs Institute guidelines [60] for scoping reviews recommend a 3-step strategy: (1) an initial search of the database (titles and abstracts) using medical subject heading terms, (2) extending the search query to other databases and adjusting the search strategy for each database, and (3) reviewing the reference list of the selected papers.

Databases were searched from January 2019 to March 2024. This period was selected to capture key developments, for example, the WHO’s first digital health guidance for youth [58], the renewed focus on adolescents through the SDGs [5], and the COVID-19 pandemic’s exposure of deep and preexisting mental health inequalities among adolescent LMICs [11,12]. Collectively, these highlighted the need for a timely review of adolescent DMHIs in low-resource countries. The search strings and results for each database are presented in Multimedia Appendix 3. The reference lists of all related reviews and included studies were hand-searched for additional papers.

### Screening

In total, 1 reviewer (CW) initially searched the databases, focusing on the first 200-300 results per database to manage the workload [68]. The most relevant papers appeared at the top of the search results. CW exported all relevant records to EndNote (Clarivate Analytics) to remove duplicate references. CW then imported the remaining records to Rayyan (Rayyan Systems Inc) for title, abstract, and full-text screening. Rayyan places all imported papers into an “undecided” category, allowing reviewers to independently “exclude,” “maybe,” or “include” each paper based on the eligibility criteria. The first screening involved only the title and abstract review (CW, LM, and CR). During the second screening, CW retrieved the full text of papers that potentially met the eligibility criteria. Where a study had a published protocol and study outcome paper, only the study outcome paper was included in the review. The relevant papers were identified by CW, LM, and CR and placed in the “include” category in the final screening step. Any discrepancies were discussed and resolved by consensus. All authors agreed that the included papers fully met the eligibility criteria.

### Data Charting and Data Items

The authors CW, LM, and ML developed and piloted a data charting form at the protocol stage. This was further refined iteratively at the review stage, and 2 reviewers (CW and CR) extracted the data (CW extracted the data and CR independently verified it). This approach is appropriate where it is not feasible for reviewers to independently chart the data [60]. Any inconsistencies were discussed and resolved. The data items extracted for each article aligned with the review aims and objectives and included the following:

1. Bibliographic information: lead author, date of publication, and country of lead author.
2. Data items related to subRQ1: DMHI name, description, target mental health disorder, start year of intervention implementation, country of intervention, participant age, sample size, study aims, intervention type, control, evaluation, outcome measures, duration, follow-up, and costs.
3. Data items related to subRQ2: adolescent involvement in DMHI design.
4. Data items related to subRQ3: consulted frameworks, theories, or models.
5. Data items related to subRQ4: factors affecting adolescent engagement with the DMHI (facilitators or barriers).
6. Key findings.

The data charting document can be found in Multimedia Appendix 4.

### Collating, Summarizing, Synthesizing, and Reporting the Results

Selected papers were reviewed to identify the similarities and differences between the DMHIs. They were then summarized and categorized into themes related to the review questions.

## Results

### Overview

**Figure 1:**
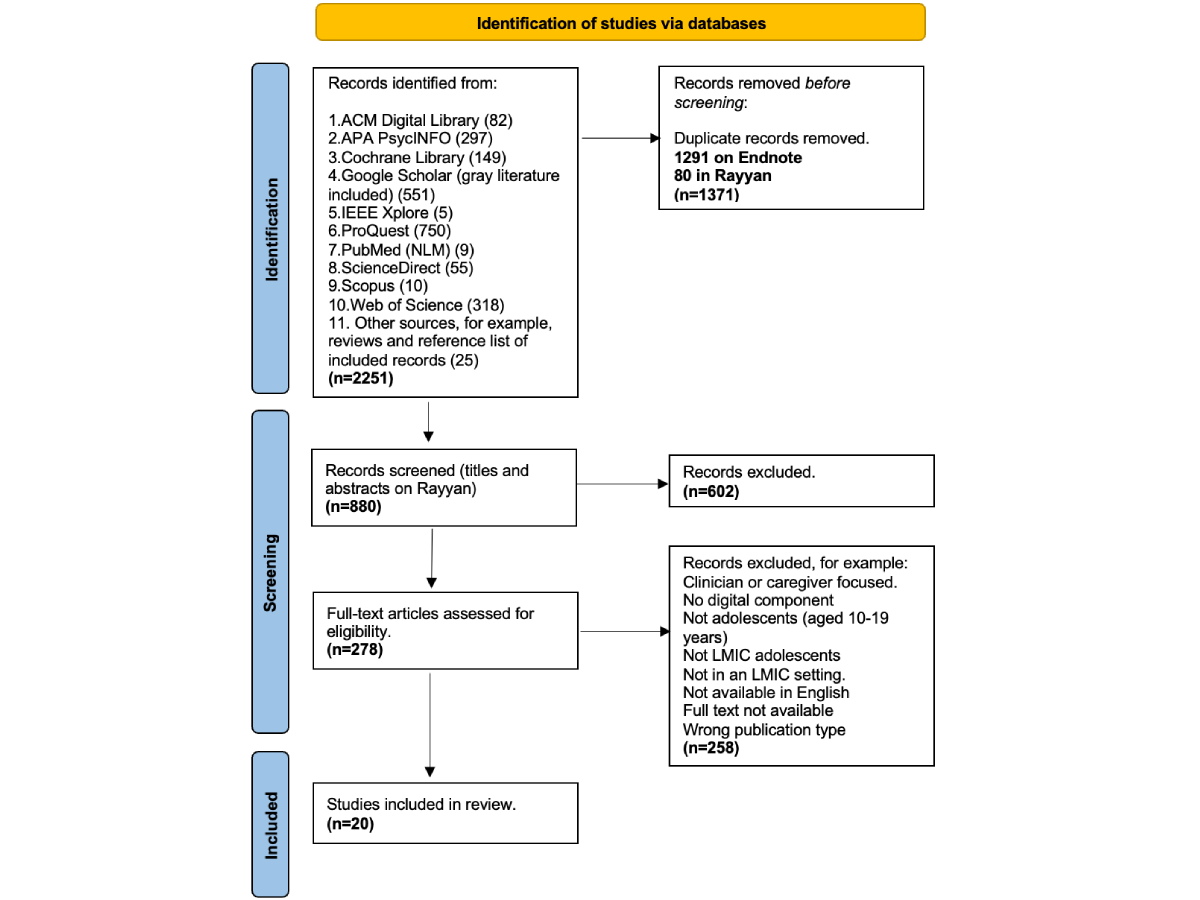
The PRISMA-ScR flowchart of the searches of 10 databases. ACM: Association for Computing Machinery; APA: American Psychological Association; IEEE: Institute of Electrical and Electronics Engineers; NLM: National Library of Medicine; LMIC: low- and middle-income country; PRISMA-ScR: Preferred Reporting Items for Systematic Reviews and Meta-Analyses Extension for Scoping Reviews.

Initial searches of 10 databases by CW in March 2024 yielded 2226 articles. Of these, 1291 (57.99%) duplicates were removed in EndNote. The remaining 935 papers were exported to Rayyan for title, abstract, and full-text screening. During the first round of screening, 80 more duplicates were removed, leaving 855 papers. CW identified 25 additional articles by searching the reference lists of excluded reviews and articles selected for inclusion. At title and abstract screening, CW reviewed 100% (880/880) of the records, and 2 reviewers (LM and CR) independently screened 25% (220/880) each, ensuring the records fulfilled the inclusion criteria. LM and CR achieved a 99% alignment score with CW’s screened papers. The detailed selection process of the articles is presented in the PRISMA-ScR flow diagram (Figure 1).

### Study Characteristics

#### Overview

A total of 20 studies were reviewed. The articles were published in English between 2019 and 2024: a total of 20% (4/20) in 2019, a total of 25% (5/20) in 2020, a total of 15% (3/20) in 2021, a total of 20% (4/20) in 2022, a total of 10% (2/20) in 2023, and 10% (2/20) in 2024. Where provided, the start year of the intervention implementation was noted: 2013, a total of 5% (1/20) [24]; 2017, a total of 5% (1/20) [69]; 2018, a total of 5% (1/20) [23]; 2019, a total of 15% (3/20) [21,56,70]; 2020, a total of 15% (3/20) [71-73]; 2021, a total of 15% (3/20) [74-76]; 2022, a total of 5% (1/20) [77]; and 2023, a total of 5% (1/20) [78]. The implementation date or year was unclear in the following references [79-84].

Geographically, the lead authors were from India (5/20, 25%); United States (5/20, 25%); Norway (3/20, 15%); and China, Colombia, Finland, Iran, Kenya, Philippines, and Switzerland (1/20, 5% each).

Concerning study design, RCTs were 25% (5/20) [22,23,56,82,84] of all studies, followed by RCT protocols (3/20, 15%) [74,75,77], qualitative studies (4/20, 20%) [69,76,79,81], mixed methods approaches (5/20, 25%) [71-73,78,80], a quasi-experimental feasibility study (1/20, 5%) [24], a multicycle usability testing approach (1/20, 5%) [70], an intervention design (1/20, 5%) [83], and a pilot study (1/20, 5%) [21]. A total of 15% (3/20) were study protocols, therefore the studies were underway.

Regarding the outcome measures, 70% (14/20) reported on primary and secondary measures, while 30% (6/20) did not. The RCTs used a total of 21 different psychometric tests, scales, and outcome measures (see Multimedia Appendix 4 for the charted data). The most common psychometric measures were the Patient Health Questionnaire-8 and Patient Health Questionnaire-9 for measuring depression and the Generalized Anxiety Disorders-7 for measuring anxiety. In 2 studies, the outcome measures were culturally validated for the target population [23,82].

In total, 100% (20/20) of studies described participants as “adolescents” aged between 10 and 19 years. Overall, the sample size was noted in 90% (18/20) of papers and ranged from 10 to 3960 participants. However, in 2 studies, 10% (2/20) did not report the sample size [76,83].

The duration of the interventions varied widely; for example, 10% (2/20) were single-session interventions [21,75], 5% (1/20) lasted 2 to 4 weeks [76], 10% (2/20) lasted 4 weeks [56,74], 5% (1/20) lasted 5 weeks [71], 5% (1/20) lasted 7 weeks [24], 5% (1/20) lasted 2 months [78], 5% (1/20) lasted 10 weeks [72], 5% (1/20) lasted 11 weeks [70], 5% (1/20) lasted 12 weeks [84], 5% (1/20) was anticipated to last between 2 and 4 months [81], 20% (4/20) lasted 6 months [23,79,80,82], and 5% (1/20) lasted 12 months [77]. The following did not specify the length of the intervention [69,73,83]. Of the 20 papers, 60% (12/20) had a follow-up period ranging from baseline to 12 months, and 40% (8/20) did not report a follow-up period. In 1 study (1/20, 5%), the authors called for a future economic evaluation of the DMHI [77]. Another paper (1/20, 5%) [76], reported on a cost-effective evaluation, noting that the cost of an incremental increase in well-being was US $37, and the cost of reducing emotional and behavioral issues was US $20. Of the other studies, 90% (18/20) did not report on the cost-effectiveness of their DMHI.

#### DMHIs

A total of 14 different DMHIs were identified, addressing one or more of the following mental health disorders: depression, 65% (13/20); well-being, 30% (6/20); emotional problem-solving, 20% (4/20); anxiety, 30% (6/20); distress, 5% (1/20); suicidal ideation, 10% (2/20); stigma, 10% (2/20); resilience, 5% (1/20); self-harm, 15% (3/20); and general mental health disorders, 10% (2/20). The main combination was depression and anxiety. Most DMHIs (13/20, 65%) were delivered or planned to be delivered in school settings, while others were delivered in clinics (2/20, 10%), hospitals (2/20, 10%), Urban Primary Health Centre in slum clusters (2/20, 10%), and refugee camps (1/20, 5%). Finally, 5% (1/20) did not specify the setting [83] (see Table 1).

**Table 1:** List of digital mental health interventions, country of implementation, settings, and mode of delivery.

Digital mental health intervention and delivery setting		Description	References
**Happy Helping Hand (Attensi Global; Lebanon, Ukraine)—schools and refugee camps**		Digital game for psychosocial support, facilitated by teachers and psychosocial support via iPads, smartphones, or computers (well-being and emotional problem-solving) Delivery: iPads, smartphones, or computers	[71,72,78]
**DepisNet-Thai (Thailand)—schools**		Web program delivered in small groups, supported by teachers (general mental health disorders) Delivery: web-based	[24]
**Intervention via WhatsApp (Meta Platforms, Inc; Kenya)—care clinics**		Intervention delivered via the WhatsApp platform with a trained pediatric HIV adherence and disclosure counselor, group chat (depression) Delivery: smartphone mobile intervention	[79,80]
**SMS text messaging intervention (China)—hospitals**		SMS text messages delivered to reduce deliberate self-harm, individual intervention (self-harm) Delivery: mobile phones	[81]
**DIALOG+S (Unit for Social and Community Psychiatry at Queen Mary University of London and the East London Foundation Trust and Pontificia Universidad Javeriana, Colombia)—schools**		A digital app-supported evidence-based intervention to assist in the interaction between patients with a mental health condition and clinicians, delivered by teachers (mental well-being and resilience) Delivery: multiple devices such as tablets or cellphones	[82]
**PRIDE^a^ research program (2016-2022)**			
	POD^b^ Adventures (India)—schools	Problem-solving game-based intervention (depression and anxiety) Delivery: smartphone	[69]
	PRIDE (2016-2022; India)—schools	Stepped-care intervention targeting common mental health problems in school-going adolescents, counselor support (general mental health conditions) Delivery: smartphone and internet-based	[74]
**STARS^c^ (South Africa)—N/A^d^**		World Health Organization transdiagnostic chatbot for distressed youths (distress) Delivery: through existing chatbot systems using different technologies (eg, apps, websites, and messaging platforms)	[83]
**Shamiri-Digital (Kenya)—schools**		Universal, computerized self-help SSI^e^, group-based, lay support (depression, anxiety, and well-being) Delivery: internet-based	[56,75]
**DAD^f^ course (Iran)—schools**		Education program, individual delivery, 6 months (8 × 30 min sessions; depression) Delivery: web-based	[23]
**ARTEMIS^g^ Intervention (India)—Urban Primary Health Centre in slum clusters**		Combines an antistigma campaign with a digital health intervention to identify and manage mental health conditions (depression, self-harm, suicide risk, or other significant emotional complaints) Delivery: tablets	[73,77]
**Kuamsha app (South Africa and Uganda)—schools**		Delivers behavioral activation to treat depression among adolescents, 6 modules taking 15-20 min to complete, supported by a brief weekly phone call from a peer mentor (depression) Delivery: smartphone	[70]
**Smartteen (India)—hospital**		Computer-assisted CBT^h^ intervention—a computer application designed to augment in-person CBT for treatment of depression in adolescents, delivered with therapist support (depression) Delivery: computer-based	[84]
**Self-help game-based mobile app intervention (Philippines)—schools**		Teaches service users skills that help them address mental health (psychosocial intervention), four 30-40-minute sessions that are spanned for 2-4 weeks, group delivery, counselor support (mild or moderate depression and anxiety) Delivery: school computer laboratories	[76]
**3 Computerized SSIs (India)—schools**		Self-guided computerized mental health intervention (depression and anxiety) Delivery: school computer laboratories	[21]

^a^PRIDE: Premium for Adolescents.

^b^POD: identifying “Problems,” generating “Options,” and creating a “Do it” plan.

^c^STARS: Sustainable Technology for Adolescents and Youth to Reduce Stress.

^d^N/A: not available.

^e^SSI: single session intervention.

^f^DAD: Dorehye Amozeshie Dokhtaran.

^g^ARTEMIS: Adolescents’ Resilience and Treatment Needs for Mental Health in Indian Slums.

^h^CBT: cognitive behavioral therapy.

Of the 20 studies, 30% (6/20) were conducted in India, 20% (4/20) in Kenya, 10% (2/20) in Lebanon, 5% (1/20) in China, 5% (1/20) in Colombia, 5% (1/20) in Iran, 5% (1/20) in the Philippines, 5% (1/20) in South Africa, 5% (1/20) in South Africa and Uganda, 5% (1/20) in Thailand, and 5% (1/20) in Ukraine. There were 4 upper-middle–income economies, 6 lower-middle–income economies, and 1 low-income economy. All met the LMIC definition set by the World Bank (see Tables 2 and 3).

**Table 2:** Country of digital mental health intervention implementation (N=20).

Study country	Studies, n (%)	References
India	6 (30)	[69,73-75,77,84]
Kenya	4 (20)	[56,75,79,80]
Lebanon	2 (10)	[71,72]
China	1 (5)	[81]
Colombia	1 (5)	[82]
Iran	1 (5)	[23]
Philippines	1 (5)	[76]
South Africa	1 (5)	[83]
South Africa and Uganda	1 (5)	[70]
Thailand	1 (5)	[24]
Ukraine	1 (5)	[78]

**Table 3:** Range of low- and middle-income countries with adolescent digital mental health interventions.

Economy type^a^	Countries, n	List of countries
Upper-middle–income economies (US $4466 to US $13,845)	4	China, Colombia, South Africa, and Thailand
Lower-middle–income economies (US $1136 to US $4465)	6	India, Iran, Kenya, Lebanon, Philippines, and Ukraine
Low-income economies (US $1135 or less)	1	Uganda

^a^World Bank Classification for the current 2024 fiscal year, low- and middle-income countries are defined as those with a gross national income per capita less than US $13,845.

### Adolescent Involvement in DMHI Design

Of the 20 papers, 60% (12/20) involved adolescents in co-designing [85] or participatory approaches at the design stage. For instance, the content of DepisNet-Thai [24] was developed from discussions with adolescents. Elsewhere, co-designing activities informed the development of POD Adventures [69], PRIDE [74], antistigma materials for ARTEMIS (Adolescents’ Resilience and Treatment Needs for Mental Health in Indian Slums) [73], and the Kuamsha app [70]. Adolescents contributed to intervention designs via group discussions for Smartteen [84] and Shamiri-Digital [21]. Some interventions were existing DMHIs adapted to the target users’ cultural contexts, for example, the Happy Helping Hand was initially launched in Norway but was developed in collaboration with adolescent Syrian refugees and Norwegian adolescents. It was later culturally adapted for use with refugee adolescents in Lebanon [71,72]. Furthermore, the Happy Helping Hand materials were also adapted for Ukrainian adolescents [78]. In an ongoing study (1/20, 5%), an Adolescent Expert Advisory Group will guide researchers on all aspects of the project design, development, and implementation [77].

### Consulted Frameworks, Theories, or Models

Of 20 studies, 60% (n=12) engaged with frameworks, toolkits, models, or theories related to behavior change interventions. These included cognitive behavioral models, social learning theory, theories of coping during adolescence, persuasive systems design, and stress-coping theory, among others. Further, 1 (5%) study consulted a digital health framework that included digital maturity and readiness content [69]. Another study referred to guidance for developing and evaluating complex interventions [73]. One paper (5%) reported how their intervention aligned with the national mental health program and the government’s national adolescent health program [77], ensuring their DMHI meets the needs and priorities of the nation’s adolescents.

### Factors Affecting Adolescent Engagement With the DMHI (Facilitators or Barriers)

A total of 90% (18/20) reported on adolescent facilitators and barriers to DMHI engagement. Further, 3 (15%) studies were underway and, therefore, had no findings or results to report at the time of the present review. However, the authors listed factors that facilitated or impeded access to DMHIs for adolescents where possible (see Textbox 2).

**Summary of barriers and facilitators (N=20).**

**Key barriers**

- COVID-19 challenges, for example, exposed adolescents’ overlooked social and personal needs.
- Complexity of app implementation, for example, lay counselor training, app development, and mental health service referrals.
- Household and school responsibilities.
- Power outages.
- Affordability, for example, insufficient phone credit, and lack of access to smartphones or mobile phones.
- Limited digital skills, low literacy levels, or both.
- Challenges with user emails reduced acceptance of the program.
- Time constraints on the app delivery by psychosocial staff or teachers.
- Boarding school attendance prevented 1 adolescent participant from participating.
- Duration of intervention.
- Stigma, discrimination, or lack of cultural appropriateness.
- Parent control of device use, for example, parents keeping adolescents’ mobile phones for most of the day.
- Lack of privacy and confidentiality.
- Concerns from teachers and parents on the time spent on phones.
- Poor local infrastructure and connectivity.
- Lack of online functionality.
- Lack of digital skills leading to a dependency on teachers or lay staff guiding the intervention.
- Device affordability, for example, phone data running out often and having to wait to top up again.
- Cost of intervention.
- Digital divide evident, for example, lack of digital device ownership and infrastructure.

**Key facilitators**

- Adapting digital mental health interventions (DMHIs) to the local context, for example, including language, cultural, and religious values.
- Embedding cultural appropriateness by design.
- Access to counselors or lay support.
- Offline access.
- Reading materials in addition to the DMHI.
- A safe space to discuss sensitive and stigmatized issues, for example, HIV-related concerns.
- Value in face-to-face interaction.
- Private school setting meant the availability of computers and internet connection.
- Family support.
- The timing of the intervention, for example, receiving a SMS text message during evenings when self-harm urges are increased.
- Peer groups comprising 10 to 15 adolescents to provide lay counseling and mental health support for adolescents in the community.
- Gamification.
- Tailored content.
- Privacy and confidentiality.
- Free to use, for example, the provision of a smartphone with the WhatsApp app preinstalled.

## Discussion

### Summary of Evidence

This scoping review aimed to explore what is known about DMHIs for adolescents in LMICs, as reported in the literature. In 2024, the World Bank categorized 134 countries as LMICs [9]. This review located only 11 countries engaged with adolescent DMHIs, illustrating the limited implementation of these solutions across LMICs and reflecting the emergent nature of the field [45]. We analyzed 20 papers to understand how these DMHIs were designed and evaluated, in what capacity adolescents were involved in the design process, what frameworks or theories were applied, and what factors impacted adolescent engagement with the DMHIs.

Overall, 14 different DMHIs were identified (POD Adventures is part of the PRIDE research program 2016-2022). DMHIs were designed for a limited range of mental health disorders, namely depression and anxiety. Almost all included adolescent input at the design stage, underscoring the importance of person-centered or user-centered approaches in DHIs. The DMHIs were delivered in diverse formats, including group delivery and self-guided methods, with lay staff or mental health professionals, including counselors, psychologists, or psychiatrists. Interventions were administered through smartphones, mobile phones, tablets, computers, and the web, often conducted in schools or clinical settings. Significant structural, psychological, and financial barriers exist in engagement with the DMHIs.

The study designs included RCTs, mixed methods, and qualitative studies. Most studies reported positive outcomes for symptom reduction, feasibility, and acceptability, measured by specific outcome measures. However, most were small scale and not trialed on a large scale over extended periods. While DMHIs were feasible and acceptable among adolescents, there remains a gap in the literature about their long-term cost- and clinical effectiveness.

### Design of Adolescent DMHIs

The DMHIs were designed for depression, anxiety, self-harm, well-being, resilience, stigma, stress, and suicide ideation. However, this is not reflective of the broad and complex range of mental health disorders that adolescents develop, including substance misuse and posttraumatic stress disorder [1,6,45]. Concerning intervention settings, most were delivered in schools or hospitals. Indeed, previous studies have recognized schools as critical settings that offer a vital entry point for receiving adolescent mental health services [86,87]. Conversely, out-of-school adolescents or those unlikely to visit those hospitals may miss the opportunity to engage with the DMHIs [88].

While RCTs remain the gold standard for evaluating the effectiveness of DHIs [89,90], they are costly and time-consuming. We identified only 5 RCTs evaluating the effectiveness of adolescent DMHIs [23,56,82,84], with a further 3 RCT protocols underway [74,75,77]. In India, Smartteen [84] was trialed on 21 adolescents with depression against a treatment-as-usual group. The DMHI was feasible, acceptable, and more effective than the treatment-as-usual at reducing symptoms at 12 weeks. Even with reduced therapist time, adolescents adhered to treatment compliance. However, the authors call for more rigorous evaluations at scale. In another study [82], DIALOG+ was adapted for adolescents in educational settings (DIALOG+S), using focus groups with teachers and adolescents. The DMHI was trialed with 70 Colombian adolescents, randomly assigned into DIALOG+S or an active control group (counseling as usual). The intervention was feasible and acceptable and could improve mental health, quality of life, and emotional symptoms. The authors call for larger studies to assess its efficacy. In Iran, the DAD (Dorehye Amozeshie Dokhtaran) [23] was trialed with 128 adolescents, randomly assigned into the DAD or a control group. The intervention showed an improvement in depression symptoms. However, the effects decreased after 12 weeks. The intervention did not affect the outcome expectations or self-efficacy. Elsewhere Shamiri-Digital [75] was trialed with 103 adolescents, randomly assigned into Shamiri-Digital or a study-skills control condition. Shamiri-Digital reduced depressive symptoms compared to the control. However, there were no significant effects on anxiety symptoms, well-being, or happiness. The authors called for replicate trials with extended follow-up periods. In a secondary analysis of this trial, the authors sought to evaluate the costs and cost-effectiveness of Shamiri-Digital through an economic evaluation [91]. Their findings indicate that Shamiri-Digital can be delivered for less than US $4 per student, which is more cost-effective than traditional interventions, for example, 12-16-week cognitive behavioral therapy sessions. However, it is difficult to draw inferences from these studies without critically assessing the quality of the evidence.

A critical design decision came from the inclusion of culturally validated elements. Some studies culturally adapted some components of the intervention [78,82]. Further, 1 study culturally validated an existing psychometric tool for their DMHI, for example, the study [24] adapted the Perceived Stress Scale to make it a reliable and valid instrument in keeping with the Thai culture. Additionally, another study [23] used the Farsi version of the Perceived Social Support Scale-Revised and the Farsi version of the Sherer General Self-Efficacy Questionnaire, reporting that the adapted scales were more appropriate and aligned to Iranian cultures. This aligns with existing literature emphasizing the importance of culturally appropriate DHIs [92,93]. Furthermore, they ensure resonance with the target populations’ beliefs, practices, and values leading to improved engagement, treatment adherence, and outcomes [10,44,65,94-96]. In contrast, the Norwegian-designed Happy Helping Hand app was not adapted to the Arab culture for Syrian adolescents, and this was detrimental to the delivery of a DMHI, as themes were deemed religiously and culturally inappropriate, and therefore, certain aspects of the DMHI were skipped [71].

An important design element was the inclusion of lay, peer, or therapist support, with adolescents preferring this option [73,76,79]. The benefits of this approach have been previously reported [55,57,97], where interventions with in-person options were more effective than self-guided or automized interventions [55].

### Adolescent Engagement With DMHI Design

The advantages of involving adolescents in participatory and co-design approaches for DMHIs are well documented [67,98-104]. In this review, for example, adolescents were engaged in a series of co-design workshops for exploring existing popular apps, for example, Temple Run (Imangi Studios) and Candy Crush (King); story building, paper prototyping, and discussions about prototype ideas for POD Adventures [69]; the cocreation of an antistigma campaign as part of the main intervention for the ARTEMIS project [77]; and 1 study incorporated feedback from recent high school graduates into the design of the group-based Shamiri-Digital intervention as an iterative process of the intervention design [56].

Collectively these studies show that participatory approaches are invaluable for generating insights for improving the DMHIs. Nevertheless, there are challenges [105], and authors are calling for the evaluation of co-design processes in diverse contexts and how that impacts technology [102,103] with clear guidance around these processes [98]. These insights will advance understanding of how and why adolescents engage in DHIs [101] and the health care outcomes of such engagements. From the review, reported activities included feasibility, acceptability, and usability studies, workshops, interviews, and focus groups. All were used clearly and meaningfully to advance the DMHI design and evaluation [24,69,70,73,74].

### Applied Frameworks and Theories

Incorporating evidence-based theories and techniques that encourage user engagement and behavior change is an essential element of DHI design [25,26,44,106]. Most DMHIs in this review were underpinned by cognitive-behavioral techniques involving behavioral activation and problem-solving features [71,72,76,78,84]. Some used stand-alone theories [23], for example, the social cognitive theory, and others used a combination of behavior change techniques [24], for example, theories of coping during adolescence, the self-determination theory, and the technology acceptance model. However, not all the papers explained how the behavior change theory or technique impacted the target user’s mental health. Or, indeed, the evidence on which the selection or combinations of theories were based [106].

Further, incorporating national or international guidance enhanced the intervention design, development, and evaluation. For example, 1 study [69] used the WHO guidelines on monitoring and evaluating DHIs, which addressed digital maturity, readiness, and scalability, whereas another [73] consulted the UK Medical Research Council guidance for developing and evaluating complex interventions focused on process evaluation and contextual factors. Finally, 1 study [77] accessed the WHO’s Mental Health Gap Action Programme-Intervention Guide, which considers training requirements and treatment protocols in low-resource settings.

### Reported Barriers and Facilitators

Textbox 2 summarizes key barriers and facilitators from the included studies. Access and engagement with DMHIs can be impacted by financial, geographical, psychological, cultural, and structural factors [107-111]. Notable barriers were the affordability of devices, cultural appropriateness, power outages, poor digital and literacy skills, inadequate internet connectivity, and limited access to devices in rural areas. The findings are consistent with other LMICs [38,44,112].

Stigma (self-stigma, eg, individual level, and societal stigma, eg, system level) remains a complex and multifaceted barrier despite the potential for increased privacy, confidentiality, anonymity, and accessibility through DMHIs [113-115]. Multiple factors contribute to persistent stigma, such as societal attitudes, the digital divide, lack of awareness, fear of exposure, discrimination, and cultural sensitivities [113,116,117]. These factors could limit engagement with DMHIs due to the negative social consequences in areas where mental health is a stigmatized issue [118,119].

Further, 1 study described how COVID-19 worsened the already dire human rights and socioeconomic conditions for displaced Syrians living in Lebanon [71], including the loss of income, food, and essential living services, disrupting education and access to learning resources. Another study [82] reported similar findings in Colombia, noting that most adolescents impacted by COVID did not have access to support and treatment. This shortage of mental health professionals and a widening treatment gap, poor education, and community knowledge reinforced the stigma and false beliefs about mental health [82].

Adolescents highlighted some facilitators, such as access to a safe space to discuss stigmatized mental health issues, culturally sensitive DMHIs that embed the local cultural and religious values by design, DMHIs that maintain face-to-face contact while offering therapist-led or lay or peer-led options, access to reading materials in addition to the DMHI, appropriate content that is entertaining, personalized, and has gamified elements, privacy and confidentiality, and DMHIs that are free to use. Previous studies have suggested that addressing the digital divide could mitigate these barriers, providing equitable access to DMHIs, yet care must be taken to ensure that the pursuit of DMHIs does not widen the existing inequalities, further reinforcing the digital divide, undermining the equitable delivery of care [120-122].

### Limitations

To the authors’ knowledge, 1 scoping review [123] has been published on DMHIs for adolescents in LMICs. Their review [123] was limited to mobile phones only; however, this review has a wider scope, which is more inclusive of the technological infrastructure in LMICs. Furthermore, this review has a more extensive search strategy, searching 10 databases and including gray literature such as dissertations and conference proceedings, and is a more robust evidence synthesis, whereas the review [123] only searched APA PsycINFO, Web of Science, Psychiatry Online, and EBSCOhost. Finally, the eligibility criteria are different, and the previous review is limited by its lack of specificity of publication parameters per publication dates. This review has been purposefully restricted to a 5-year period (2019-2024), as specified earlier in this paper.

There are some limitations to the present review; 1 reviewer performed the database searches, which may have introduced reviewer bias. However, 2 more reviewers independently screened 25% (220/880) of each of the titles and abstracts of potential papers against the eligibility criteria. This step reduces bias and adds credibility and rigor to the review process. In addition, the quality of included studies was not appraised or assessed for risk of bias therefore, there is no assurance of the quality of the evidence in this scoping review. This is consistent with the aim of a scoping review, which is to rapidly map the available literature and not perform a systematic analysis. Finally, the review was limited to papers published in English. This may have significantly reduced the number of eligible studies from LMICs.

### Conclusions

The global shortage of access to mental health treatment demonstrates the critical need for effective, low-cost, scalable solutions. The COVID-19 pandemic has highlighted the need for alternative solutions, and leveraging digital technologies through the international frameworks of the UHC and SDGs can alleviate the significant burden of mental health inequalities among adolescents in LMICs.

In this review, adolescent involvement in various stages of the theory-based design and evaluation cycle enhanced the intervention’s acceptability, feasibility, and usefulness. While RCTs of DMHIs remain the gold standard in this field, they are sparse. Researchers have called for repeat trials at larger scales in diverse settings to assess their effectiveness, scalability, and feasibility. Moreover, only some of the tools and instruments used to evaluate the DMHIs were culturally validated in LMIC contexts, making it difficult to establish how the DMHI would benefit adolescents beyond the test settings.

Addressing the digital divide and related barriers, such as stigma, will be a critical challenge to ensuring equitable access to universal and affordable mental health care. Understanding the long-term clinical implications and the cost-effectiveness of DMHIs will be another challenge for future research because that is an essential consideration for sustainable DMHIs in resource-constrained settings.

## Appendix

Multimedia Appendix 1 PRISMA-ScR (Preferred Reporting Items for Systematic Reviews and Meta-Analyses extension for Scoping Reviews) checklist.

Multimedia Appendix 2 Google Scholar, custom range search date (2019-2024).

Multimedia Appendix 3 The search strings and results for each database.

Multimedia Appendix 4 The data charting document.

## Abbreviations

ARTEMIS: Adolescents’ Resilience and Treatment Needs for Mental Health in Indian Slums
DHI: digital health intervention
DMHI: digital mental health intervention
GSM: Global System for Mobile
LMIC: low- and middle-income country
PCC: population, concept, and context
POD: identifying “Problems,” generating “Options,” and creating a “Do it” plan
PRIDE: Premium for Adolescents
PRISMA-ScR: Preferred Reporting Items for Systematic Reviews and Meta-Analyses Extension for Scoping Reviews
RCT: randomized controlled trial
SDG: Sustainable Development Goal
subRQ: subreview question
UHC: universal health coverage
UNICEF: United Nations International Children’s Emergency Fund
WHO: World Health Organization
