# Supplementary material for: Digital Mental Health Interventions for Adolescents in Low- and Middle-Income Countries: Scoping Review
Source: J Med Internet Res. 2024 Oct 29;26:e51376. doi: 10.2196/51376 (PMC11558223; doi:10.2196/51376)
Supplement: Multimedia Appendix 2 [file jmir_v26i1e51376_app2.docx]

(“digital health intervention*” AND “mental health” AND “adolescent*” AND “low and middle income”)

- **127 records:** [**LINK**](https://scholar.google.co.uk/scholar?hl=en&as_sdt=0%2C5&as_ylo=2019&as_yhi=2024&q=%28%E2%80%9Cdigital+health+intervention*%E2%80%9D+AND+%E2%80%9Cmental+health%E2%80%9D+AND+%E2%80%9Cadolescent*%E2%80%9D+AND+%E2%80%9Clow+and+middle+income%E2%80%9D%29&btnG=)

(“digital mental health intervention*” AND “adolescent*” AND “low and middle income OR LMIC*” AND (“co-design” OR "participatory") AND “evaluation”)

- **37 records:** [**LINK**](https://scholar.google.co.uk/scholar?q=%28%E2%80%9Cdigital+mental+health+intervention*%E2%80%9D+AND+%E2%80%9Cadolescent*%E2%80%9D+AND+%E2%80%9Clow+and+middle+income+OR+LMIC*%E2%80%9D+AND+%28%E2%80%9Cco-design%E2%80%9D+OR+%22participatory%22%29+AND+%E2%80%9Cevaluation%E2%80%9D%29&hl=en&as_sdt=0%2C5&as_ylo=2019&as_yhi=2024)

("digital mental health intervention*" OR (“digital health intervention” AND “mental health”) AND (“adolescen*” OR "young adult" OR “teen*”) AND ("low and middle income" OR "developing countr*" OR “LMIC*”))

- **64 records:** [**LINK**](https://scholar.google.co.uk/scholar?q=%28%22digital+mental+health+intervention*%22+OR+%28%E2%80%9Cdigital+health+intervention%E2%80%9D+AND+%E2%80%9Cmental+health%E2%80%9D%29+AND+%28%E2%80%9Cadolescen*%E2%80%9D+OR+%22young+adult%22+OR+%E2%80%9Cteen*%E2%80%9D%29+AND+%28%22low+and+middle+income%22+OR+%22developing+countr*%22+OR+%E2%80%9CLMIC*%E2%80%9D%29%29&hl=en&as_sdt=0%2C5&as_ylo=2019&as_yhi=2024)

("digital mental health intervention*" OR ("digital health intervention" AND "mental health") AND (mhealth OR ehealth OR smart* OR "mobile app*" OR "web app*" OR "health information system*" OR "computer-assisted" OR app*) AND (adolescen* OR "young adult" OR child* OR "teen*") AND ("low and middle income" OR "developing countr*" OR LMIC*))

- **244 records:** [**LINK**](https://scholar.google.co.uk/scholar?q=%28%22digital+mental+health+intervention*%22+OR+%28%22digital+health+intervention%22+AND+%22mental+health%22%29+AND+%28mhealth+OR+ehealth+OR+smart*+OR+%22mobile+app*%22+OR+%22web+app*%22+OR+%22health+information+system*%22+OR+%22computer-assisted%22+OR+app*%29+AND+%28adolescen*+OR+%22young+adult%22+OR+child*+OR+%22teen*%22%29+AND+%28%22low+and+middle+income%22+OR+%22developing+countr*%22+OR+LMIC*%29%29&hl=en&as_sdt=0%2C5&as_ylo=2019&as_yhi=2024)

((“digital mental health intervention*” OR (“digital health intervention” AND “mental health”) OR “mental disorder*” OR “psychosocial intervention” OR “depressive disorder*” OR depression OR anxiet* OR “mood disorder*” OR “substance-related disorder*” OR “self-concept” OR “feeding and eating disorder*” OR “risk-taking” OR suicide) AND (smart* OR “web app*” OR “mobile app*” OR “health information system*” OR “computer-assisted” OR “cell phone” OR “Internet” OR “virtual” OR “artificial” OR “social media” OR “remote consultation” OR “video gam*”) AND (“adolescen*” OR “child*” OR “young adult” OR “teen*” OR “young people”) AND (“low and middle income” OR “developing countr*” OR "LMIC*") AND (“co-design" OR "participatory”) AND (“evaluation”))

- **150 records:** [**LINK**](https://scholar.google.co.uk/scholar?q=%28%28%E2%80%9Cdigital+mental+health+intervention*%E2%80%9D+OR+%28%E2%80%9Cdigital+health+intervention%E2%80%9D+AND+%E2%80%9Cmental+health%E2%80%9D%29+OR+%E2%80%9Cmental+disorder*%E2%80%9D+OR+%E2%80%9Cpsychosocial+intervention%E2%80%9D+OR+%E2%80%9Cdepressive+disorder*%E2%80%9D+OR+depression+OR+anxiet*+OR+%E2%80%9Cmood+disorder*%E2%80%9D+OR+%E2%80%9Csubstance-related+disorder*%E2%80%9D+OR+%E2%80%9Cself-concept%E2%80%9D+OR+%E2%80%9Cfeeding+and+eating+disorder*%E2%80%9D+OR+%E2%80%9Crisk-taking%E2%80%9D+OR+suicide%29+AND+%28smart*+OR+%E2%80%9Cweb+app*%E2%80%9D+OR+%E2%80%9Cmobile+app*%E2%80%9D+OR+%E2%80%9Chealth+information+system*%E2%80%9D+OR+%E2%80%9Ccomputer-assisted%E2%80%9D+OR+%E2%80%9Ccell+phone%E2%80%9D+OR+%E2%80%9CInternet%E2%80%9D+OR+%E2%80%9Cvirtual%E2%80%9D+OR+%E2%80%9Cartificial%E2%80%9D+OR+%E2%80%9Csocial+media%E2%80%9D+OR+%E2%80%9Cremote+consultation%E2%80%9D+OR+%E2%80%9Cvideo+gam*%E2%80%9D%29+AND+%28%E2%80%9Cadolescen*%E2%80%9D+OR+%E2%80%9Cchild*%E2%80%9D+OR+%E2%80%9Cyoung+adult%E2%80%9D+OR+%E2%80%9Cteen*%E2%80%9D+OR+%E2%80%9Cyoung+people%E2%80%9D%29+AND+%28%E2%80%9Clow+and+middle+income%E2%80%9D+OR+%E2%80%9Cdeveloping+countr*%E2%80%9D+OR+%22LMIC*%22%29+AND+%28%E2%80%9Cco-design%22+OR+%22participatory%E2%80%9D%29+AND+%28%E2%80%9Cevaluation%E2%80%9D%29%29&hl=en&as_sdt=0%2C5&as_ylo=2019&as_yhi=2024)

((("digital mental health intervention*" OR ("digital health intervention" AND "mental health") OR "mental disorder*" OR "psychosocial intervention" OR "depressive disorder*" OR depression OR anxiet* OR "mood disorder*" OR "substance" OR "self-concept" OR "feeding and eating disorder*" OR "risk-taking" OR suicide) AND (smart* OR "web app*" OR "mobile app*" OR "health information system*" OR "computer-assisted" OR "cell phone" OR "Internet" OR "virtual" OR "artificial" OR "social media" OR "remote consultation" OR "video gam*")) AND (adolescents OR "adolescent") AND ("low and middle income" OR "developing countr*" OR "LMIC*") AND (co-design OR participatory) AND "evaluation")

- **58 records:** [**LINK**](https://scholar.google.co.uk/scholar?q=%28%28%28%22digital+mental+health+intervention*%22+OR+%28%22digital+health+intervention%22+AND+%22mental+health%22%29+OR+%22mental+disorder*%22+OR+%22psychosocial+intervention%22+OR+%22depressive+disorder*%22+OR+depression+OR+anxiet*+OR+%22mood+disorder*%22+OR+%22substance%22+OR+%22self-concept%22+OR+%22feeding+and+eating+disorder*%22+OR+%22risk-taking%22+OR+suicide%29+AND+%28smart*+OR+%22web+app*%22+OR+%22mobile+app*%22+OR+%22health+information+system*%22+OR+%22computer-assisted%22+OR+%22cell+phone%22+OR+%22Internet%22+OR+%22virtual%22+OR+%22artificial%22+OR+%22social+media%22+OR+%22remote+consultation%22+OR+%22video+gam*%22%29%29+AND+%28adolescents+OR+%22adolescent%22%29+AND+%28%22low+and+middle+income%22+OR+%22developing+countr*%22+OR+%22LMIC*%22%29+AND+%28co-design+OR+participatory%29+AND+%22evaluation%22%29&hl=en&as_sdt=0%2C5&as_ylo=2019&as_yhi=2024)

The total number of records to be exported to EndNote for deduplication is **680**. After the duplicates are removed, the remaining records should be imported to Rayyan for screening.
