# Supplementary material for: Digital Mental Health Interventions for Adolescents in Low- and Middle-Income Countries: Scoping Review
Source: J Med Internet Res. 2024 Oct 29;26:e51376. doi: 10.2196/51376 (PMC11558223; doi:10.2196/51376)
Supplement: Multimedia Appendix 3 [file jmir_v26i1e51376_app3.docx]

### Development of the search strings using MeSH terms and keywords.

The following databases were searched between Jan 2019 – March 2024:

1. ACM Digital Library

2. APA PsycINFO

3. Cochrane Library

4. Google Scholar (grey literature included)

5. IEEE Xplore

6. ProQuest

7. PubMed (NLM)

8. ScienceDirect

9. SCOPUS

10. Web of Science

### Concept 1: Digital Health Intervention

*MeSH Terms:*

"Telemedicine"[Mesh], "Smartphone"[Mesh], "Mobile Applications"[Mesh], "Health Information Systems"[Mesh], "Artificial Intelligence"[Mesh], "Therapy, Computer-Assisted"[Mesh], "Cell Phone"[Mesh]

*Text Words:*

“Digital health solution*”[Text Word], “digital health intervention*”[Text Word], “human computer interaction” [Text Word], smartphone*[Text Word], “mobile technolog*”[Text Word], “mobile app*”[Text Word], “health app*”[Text Word], “smart device*”[Text Word]

### Concept 2: Mental Health

*MeSH Terms:*

"mental health"[MeSH Terms], "mental disorders"[MeSH Terms], "psychosocial intervention"[MeSH Terms], "depressive disorder"[MeSH Terms], "depression"[MeSH Terms], "anxiety"[MeSH Terms], "mood disorders"[MeSH Terms], "substance-related disorders"[MeSH Terms], "self-concept"[MeSH Terms], "feeding and eating disorders"[MeSH Terms], "risk-taking"[MeSH Terms], "suicide"[MeSH Terms]

*Text Words:*

mental health[Text Word], mental disorder[Text Word], psychosocial intervention[Text Word], depression[Text Word], anxiety[Text Word], Trauma and Stressor Related Disorders [Text Word], mood disorders[Text Word], substance related disorders[Text Word], self-esteem[Text Word], eating disorder[Text Word], risk taking[Text Word], suicide[Text Word]

### Concept 3: Adolescents

*MeSH Terms:*

"adolescent"[MeSH Terms], "child"[MeSH Terms], "young adult"[MeSH Terms]

*Text Words:*

Adolescent[Text Word], child[Text Word], young adult[Text Word], youth[Text Word], teen[Text Word

### Concept 4: Low- and Middle-Income Countries

*MeSH Terms:*

"developing countries"[MeSH Terms]

*Text Words:*

developing countries[Text Word], Global south [Text Word], low-economic countr* [Text Word], LMIC* [Text Word]

### Database searches

The databases were then searched using a combination of the MeSH terms and keywords/search strings, adapted per database rules. All databases were searched with the following six commands e.g.

Search 1: (“digital mental health intervention” AND “adolescents” AND “low and middle income”)

Search 2: (“digital mental health intervention” AND “adolescents” AND “low and middle income” AND “co-design” AND “implementation”)

Search 3: (("digital mental health intervention*") AND ( “adolescen*” OR "young adult" ) AND ("low and middle income" OR "developing countr*" OR LMICs))

Search 4: ("digital mental health intervention*" OR (“digital health intervention” AND “mental health”) AND (“adolescen*” OR "young adult" OR “teen*”) AND ("low and middle income" OR ("developing countries" OR "developing country") OR LMICs))

Search 5: ("digital mental health intervention*" OR (“digital health intervention” AND “mental health”) AND (mhealth OR ehealth OR smart* OR mobile app* OR web app* OR “health information system*” OR “therapy computer assisted” OR app*) AND (“adolescen*” OR "young adult" OR child* OR teen*) AND ("low and middle income" OR "developing countr*" OR “LMICs”))

Search 6: ((“digital mental health intervention*” OR (“digital health intervention” AND “mental health”) OR “mental disorder*” OR “psychosocial intervention” OR “depressive disorder*” OR depression OR anxiet* OR “mood disorder*” OR “substance-related disorder*” OR “self-concept” OR “feeding and eating disorder*” OR “risk-taking” OR suicide) AND (smart* OR “web app*” OR “mobile app*” OR “health information system*” OR “therapy computer assisted” OR “cell phone” OR “Internet” OR “social media” OR “Internet” OR “remote consultation” OR “video game*”) AND (“adolescen*” OR “child*” OR “young adult” OR “teen*”) AND (“low and middle income” OR “developing countr*”) AND (“co-design”) AND (“implementation”))

1. **ACM Digital**
2. (“digital health intervention*” AND “mental health” AND “adolescent*” AND “low and middle income”)

1 Results for: [All: "digital health intervention*"] AND [All: "mental health"] AND [All: "adolescent*"] AND [All: "low and middle income"] AND [E-Publication Date: Past 5 years]

[LINK](https://dl.acm.org/action/doSearch?fillQuickSearch=false&target=advanced&expand=dl&field1=AllField&text1=%28%E2%80%9Cdigital+health+intervention*%E2%80%9D+AND+%E2%80%9Cmental+health%E2%80%9D+AND+%E2%80%9Cadolescent*%E2%80%9D+AND+%E2%80%9Clow+and+middle+income%E2%80%9D%29&EpubDate=%5B20190506+TO+20240506%5D)

1. (“digital mental health intervention*” AND “adolescent*” AND “low and middle income OR LMIC*” AND (“co-design” OR "participatory") AND “evaluation”)

0 Results for: [All: "digital mental health intervention*"] AND [All: "adolescent*"] AND [All: "low and middle income or lmic*"] AND [[All: "co-design"] OR [All: "participatory"]] AND [All: "evaluation"] AND [E-Publication Date: Past 5 years]

[LINK](https://dl.acm.org/action/doSearch?fillQuickSearch=false&target=advanced&expand=dl&field1=AllField&text1=%28%E2%80%9Cdigital+mental+health+intervention*%E2%80%9D+AND+%E2%80%9Cadolescent*%E2%80%9D+AND+%E2%80%9Clow+and+middle+income+OR+LMIC*%E2%80%9D+AND+%28%E2%80%9Cco-design%E2%80%9D+OR+%22participatory%22%29+AND+%E2%80%9Cevaluation%E2%80%9D%29&EpubDate=%5B20190506+TO+20240506%5D)

1. ("digital mental health intervention*" OR (“digital health intervention” AND “mental health”) AND (“adolescen*” OR "young adult" OR “teen*”) AND ("low and middle income" OR "developing countr*" OR “LMIC*”))

8 Results for: [All: "digital mental health intervention*"] OR [[All: "digital health intervention"] AND [All: "mental health"] AND [[All: "adolescen*"] OR [All: "young adult"] OR [All: "teen*"]] AND [[All: "low and middle income"] OR [All: "developing countr*"] OR [All: "lmic*"]]] AND [E-Publication Date: Past 5 years]

[LINK](https://dl.acm.org/action/doSearch?fillQuickSearch=false&target=advanced&expand=dl&EpubDate=%5B20190506+TO+20240506%5D&AllField=%28%22digital+mental+health+intervention*%22+OR+%28%22digital+health+intervention%22+AND+%22mental+health%22%29+AND+%28%22adolescen*%22+OR+%22young+adult%22+OR+%22teen*%22%29+AND+%28%22low+and+middle+income%22+OR+%22developing+countr*%22+OR+%22LMIC*%22%29%29)

1. (("digital mental health intervention”) OR (“digital health intervention” AND “mental health”) AND (“mhealth” OR “ehealth” OR smart* OR mobile app* OR web app* OR health information system* OR “computer-assisted” OR app*) AND (adolescen* OR "young adult" OR child* OR teen*) AND ("low and middle income" OR developing countr* OR LMIC*))

16 Results for: [All: "digital mental health intervention"] OR [[All: "digital health intervention"] AND [All: "mental health"] AND [[All: "mhealth"] OR [All: "ehealth"] OR [All: smart*] OR [All: mobile app*] OR [All: web app*] OR [All: health information system*] OR [All: "computer-assisted"] OR [All: app*]] AND [[All: adolescen*] OR [All: "young adult"] OR [All: child*] OR [All: teen*]] AND [[All: "low and middle income"] OR [All: developing countr*] OR [All: lmic*]]] AND [E-Publication Date: Past 5 years]

[LINK](https://dl.acm.org/action/doSearch?fillQuickSearch=false&target=advanced&expand=dl&EpubDate=%5B20190506+TO+20240506%5D&AllField=%28%28%22digital+mental+health+intervention%22%29+OR+%28%22digital+health+intervention%22+AND+%22mental+health%22%29+AND+%28%22mhealth%22+OR+%22ehealth%22+OR+smart*+OR+mobile+app*+OR+web+app*+OR+health+information+system*+OR+%22computer-assisted%22+OR+app*%29+AND+%28adolescen*+OR+%22young+adult%22+OR+child*+OR+teen*%29+AND+%28%22low+and+middle+income%22+OR+developing+countr*+OR+LMIC*%29%29)

1. ((“digital mental health intervention*” OR (“digital health intervention” AND “mental health”) OR “mental disorder*” OR “psychosocial intervention” OR “depressive disorder*” OR depression OR anxiet* OR “mood disorder*” OR “substance-related disorder*” OR “self-concept” OR “feeding and eating disorder*” OR “risk-taking” OR suicide) AND (smart* OR “web app*” OR “mobile app*” OR “health information system*” OR “computer-assisted” OR “cell phone” OR “Internet” OR “virtual” OR “artificial” OR “social media” OR “remote consultation” OR “video gam*”) AND (“adolescen*” OR “child*” OR “young adult” OR “teen*” OR “young people”) AND (“low and middle income” OR “developing countr*” OR "LMIC*") AND (“co-design" OR "participatory”) AND (“evaluation”))

33 Results for: [[All: "digital mental health intervention*"] OR [[All: "digital health intervention"] AND [All: "mental health"]] OR [All: "mental disorder*"] OR [All: "psychosocial intervention"] OR [All: "depressive disorder*"] OR [All: depression] OR [All: anxiet*] OR [All: "mood disorder*"] OR [All: "substance-related disorder*"] OR [All: "self-concept"] OR [All: "feeding and eating disorder*"] OR [All: "risk-taking"] OR [All: suicide]] AND [[All: smart*] OR [All: "web app*"] OR [All: "mobile app*"] OR [All: "health information system*"] OR [All: "computer-assisted"] OR [All: "cell phone"] OR [All: "internet"] OR [All: "virtual"] OR [All: "artificial"] OR [All: "social media"] OR [All: "remote consultation"] OR [All: "video gam*"]] AND [[All: "adolescen*"] OR [All: "child*"] OR [All: "young adult"] OR [All: "teen*"] OR [All: "young people"]] AND [[All: "low and middle income"] OR [All: "developing countr*"] OR [All: "lmic*"]] AND [[All: "co-design"] OR [All: "participatory"]] AND [All: "evaluation"] AND [E-Publication Date: Past 5 years]

[LINK](https://dl.acm.org/action/doSearch?fillQuickSearch=false&target=advanced&expand=dl&EpubDate=%5B20190506+TO+20240506%5D&AllField=%28%22digital+mental+health+intervention*%22+OR+%28%22digital+health+intervention%22+AND+%22mental+health%22%29+OR+%22mental+disorder*%22+OR+%22psychosocial+intervention%22+OR+%22depressive+disorder*%22+OR+depression+OR+anxiet*+OR+%22mood+disorder*%22+OR+%22substance%22+OR+%22self-concept%22+OR+%22feeding+and+eating+disorder*%22+OR+%22risk-taking%22+OR+%22suicide%22%29+AND+%28smart*+OR+%22web+app*%22+OR+%22mobile+app*%22+OR+%22health+information+system*%22+OR+%22computer-assisted%22+OR+%22cell+phone%22+OR+%22Internet%22+OR+%22virtual%22+OR+%22artificial%22+OR+%22social+media%22+OR+%22remote+consultation%22+OR+%22video+gam*%22%29+AND+%28%22adolescents%22+OR+%22adolescent%22%29+AND+%28%22low+and+middle+income%22+OR+%22developing+countr*%22+OR+%22LMIC*%22%29+AND+%28%22co-design%22+OR+%22participatory%22%29+AND+%22evaluation%22%29)

1. (“digital mental health intervention*” OR (“digital health intervention” AND “mental health”) OR “mental disorder*” OR “psychosocial intervention” OR “depressive disorder*” OR depression OR anxiet* OR “mood disorder*” OR “substance” OR “self-concept” OR “feeding and eating disorder*” OR “risk-taking” OR "suicide") AND (smart* OR “web app*” OR “mobile app*” OR “health information system*” OR “computer-assisted” OR “cell phone” OR “Internet” OR “virtual” OR “artificial” OR “social media” OR “remote consultation” OR “video gam*”) AND (“adolescents” OR "adolescent") AND (“low and middle income” OR “developing countr*” OR "LMIC*") AND (“co-design" OR "participatory”) AND "evaluation")

25 Results for: [[[All: "digital mental health intervention*"] OR [[All: "digital health intervention"] AND [All: "mental health"]] OR [All: "mental disorder*"] OR [All: "psychosocial intervention"] OR [All: "depressive disorder*"] OR [All: depression] OR [All: anxiet*] OR [All: "mood disorder*"] OR [All: "substance"] OR [All: "self-concept"] OR [All: "feeding and eating disorder*"] OR [All: "risk-taking"] OR [All: "suicide"]] AND [[All: smart*] OR [All: "web app*"] OR [All: "mobile app*"] OR [All: "health information system*"] OR [All: "computer-assisted"] OR [All: "cell phone"] OR [All: "internet"] OR [All: "virtual"] OR [All: "artificial"] OR [All: "social media"] OR [All: "remote consultation"] OR [All: "video gam*"]] AND [[All: "adolescents"] OR [All: "adolescent"]] AND [[All: "low and middle income"] OR [All: "developing countr*"] OR [All: "lmic*"]] AND [[All: "co-design"] OR [All: "participatory"]] AND [All: "evaluation"]] OR [All: ))] AND [E-Publication Date: Past 5 years]

[LINK](https://dl-acm-org.proxy.lib.strath.ac.uk/action/doSearch?AllField=%28%E2%80%9Cdigital+mental+health+intervention*%E2%80%9D+OR+%28%E2%80%9Cdigital+health+intervention%E2%80%9D+AND+%E2%80%9Cmental+health%E2%80%9D%29+OR+%E2%80%9Cmental+disorder*%E2%80%9D+OR+%E2%80%9Cpsychosocial+intervention%E2%80%9D+OR+%E2%80%9Cdepressive+disorder*%E2%80%9D+OR+depression+OR+anxiet*+OR+%E2%80%9Cmood+disorder*%E2%80%9D+OR+%E2%80%9Csubstance%E2%80%9D+OR+%E2%80%9Cself-concept%E2%80%9D+OR+%E2%80%9Cfeeding+and+eating+disorder*%E2%80%9D+OR+%E2%80%9Crisk-taking%E2%80%9D+OR+%22suicide%22%29+AND+%28smart*+OR+%E2%80%9Cweb+app*%E2%80%9D+OR+%E2%80%9Cmobile+app*%E2%80%9D+OR+%E2%80%9Chealth+information+system*%E2%80%9D+OR+%E2%80%9Ccomputer-assisted%E2%80%9D+OR+%E2%80%9Ccell+phone%E2%80%9D+OR+%E2%80%9CInternet%E2%80%9D+OR+%E2%80%9Cvirtual%E2%80%9D+OR+%E2%80%9Cartificial%E2%80%9D+OR+%E2%80%9Csocial+media%E2%80%9D+OR+%E2%80%9Cremote+consultation%E2%80%9D+OR+%E2%80%9Cvideo+gam*%E2%80%9D%29+AND+%28%E2%80%9Cadolescents%E2%80%9D+OR+%22adolescent%22%29+AND+%28%E2%80%9Clow+and+middle+income%E2%80%9D+OR+%E2%80%9Cdeveloping+countr*%E2%80%9D+OR+%22LMIC*%22%29+AND+%28%E2%80%9Cco-design%22+OR+%22participatory%E2%80%9D%29+AND+%22evaluation%22%29&startPage=&EpubDate=%5B20190323%20TO%20202403232359%5D&queryID=45/6684404593)

1. **APA PsychINFO (EBSCO)**
2. (“digital health intervention*” AND “mental health” AND “adolescent*” AND “low and middle income”)

(“digital health intervention*” AND “mental health” AND “adolescent*” AND “low and middle income”) Limiters - Publication Date: 20190101-20241231

Expanders - Apply equivalent subjects

Search modes - Find all my search terms View Results (2)

[LINK](https://web.p.ebscohost.com/ehost/resultsadvanced?vid=6&sid=b1537aad-8152-4f5d-894f-bb295d2669c2%40redis)

1. (“digital mental health intervention*” AND “adolescent*” AND “low and middle income OR LMIC*” AND (“co-design” OR "participatory") AND “evaluation”)

(“digital mental health intervention*” AND “adolescent*” AND “low and middle income OR LMIC*” AND (“co-design” OR "participatory") AND “evaluation”) Limiters - Publication Date: 20190101-20241231

Expanders - Apply equivalent subjects

Search modes - SmartText Searching View Results (488)

[LINK](https://web.p.ebscohost.com/ehost/resultsadvanced?sid=b1537aad-8152-4f5d-894f-bb295d2669c2%40redis&vid=12&HistoryItemID=S5&bquery=(%e2%80%9cdigital+mental+health+intervention*%e2%80%9d+AND+%e2%80%9cadolescent*%e2%80%9d+AND+%e2%80%9clow+and+middle+income+OR+LMIC*%e2%80%9d+AND+(%e2%80%9cco-design%e2%80%9d+OR+%22participatory%22)+AND+%e2%80%9cevaluation%e2%80%9d)&bdata=JmRiPXBzeWgmY2xpMD1EVDEmY2x2MD0yMDE5MDEtMjAyNDEyJmF1dGh0eXBlPXNoaWImdHlwZT0xJnNlYXJjaE1vZGU9UmVsZXZhbmN5JnNpdGU9ZWhvc3QtbGl2ZQ%3d%3d#resultListAnchor)

1. ("digital mental health intervention*" OR (“digital health intervention” AND “mental health”) AND (“adolescen*” OR "young adult" OR “teen*”) AND ("low and middle income" OR "developing countr*" OR “LMIC*”))

("digital mental health intervention*" OR (“digital health intervention” AND “mental health”) AND (“adolescen*” OR "young adult" OR “teen*”) AND ("low and middle income" OR "developing countr*" OR “LMIC*”)) Limiters - Publication Date: 20190101-20241231

Expanders - Apply equivalent subjects

Search modes - Find all my search terms View Results (111)

[LINK](https://web.p.ebscohost.com/ehost/resultsadvanced?vid=21&sid=b1537aad-8152-4f5d-894f-bb295d2669c2%40redis&bquery=(%22digital+mental+health+intervention*%22+OR+(%e2%80%9cdigital+health+intervention%e2%80%9d+AND+%e2%80%9cmental+health%e2%80%9d)+AND+(%e2%80%9cadolescen*%e2%80%9d+OR+%22young+adult%22+OR+%e2%80%9cteen*%e2%80%9d)+AND+(%22low+and+middle+income%22+OR+%22developing+countr*%22+OR+%e2%80%9cLMIC*%e2%80%9d))&bdata=JmRiPXBzeWgmY2xpMD1EVDEmY2x2MD0yMDE5MDEtMjAyNDEyJmF1dGh0eXBlPXNoaWImdHlwZT0xJnNlYXJjaE1vZGU9QW5kJnNpdGU9ZWhvc3QtbGl2ZQ%3d%3d)

1. (("digital mental health intervention”) OR (“digital health intervention” AND “mental health”) AND (“mhealth” OR “ehealth” OR smart* OR mobile app* OR web app* OR health information system* OR “computer-assisted” OR app*) AND (adolescen* OR "young adult" OR child* OR teen*) AND ("low and middle income" OR developing countr* OR LMIC*))

(("digital mental health intervention”) OR (“digital health intervention” AND “mental health”) AND (“mhealth” OR “ehealth” OR smart* OR mobile app* OR web app* OR health information system* OR “computer-assisted” OR app*) AND (adolescen* OR "young adult" OR child* OR teen*) AND ("low and middle income" OR developing countr* OR LMIC*)) Limiters - Publication Date: 20190101-20241231

Expanders - Apply equivalent subjects

Search modes - Find all my search terms View Results (34)

[LINK](https://web.p.ebscohost.com/ehost/resultsadvanced?vid=23&sid=b1537aad-8152-4f5d-894f-bb295d2669c2%40redis&bquery=((%22digital+mental+health+intervention%e2%80%9d)+OR+(%e2%80%9cdigital+health+intervention%e2%80%9d+AND+%e2%80%9cmental+health%e2%80%9d)+AND+(%e2%80%9cmhealth%e2%80%9d+OR+%e2%80%9cehealth%e2%80%9d+OR+smart*+OR+mobile+app*+OR++web+app*+OR+health+information+system*+OR+%e2%80%9ccomputer-assisted%e2%80%9d+OR+app*)+AND+(adolescen*+OR+%22young+adult%22+OR+child*+OR+teen*)+AND+(%22low+and+middle+income%22++OR++developing+countr*+OR+LMIC*))&bdata=JmRiPXBzeWgmY2xpMD1EVDEmY2x2MD0yMDE5MDEtMjAyNDEyJmF1dGh0eXBlPXNoaWImdHlwZT0xJnNlYXJjaE1vZGU9QW5kJnNpdGU9ZWhvc3QtbGl2ZQ%3d%3d)

1. ((“digital mental health intervention*” OR (“digital health intervention” AND “mental health”) OR “mental disorder*” OR “psychosocial intervention” OR “depressive disorder*” OR depression OR anxiet* OR “mood disorder*” OR “substance-related disorder*” OR “self-concept” OR “feeding and eating disorder*” OR “risk-taking” OR suicide) AND (smart* OR “web app*” OR “mobile app*” OR “health information system*” OR “computer-assisted” OR “cell phone” OR “Internet” OR “virtual” OR “artificial” OR “social media” OR “remote consultation” OR “video gam*”) AND (“adolescen*” OR “child*” OR “young adult” OR “teen*” OR “young people”) AND (“low and middle income” OR “developing countr*” OR "LMIC*") AND (“co-design" OR "participatory”) AND (“evaluation”))

((“digital mental health intervention*” OR (“digital health intervention” AND “mental health”) OR “mental disorder*” OR “psychosocial intervention” OR “depressive disorder*” OR depression OR anxiet* OR “mood disorder*” OR “substance-related disorder*” OR “self-concept” OR “feeding and eating disorder*” OR “risk-taking” OR suicide) AND (smart* OR “web app*” OR “mobile app*” OR “health information system*” OR “computer-assisted” OR “cell phone” OR “Internet” OR “virtual” OR “artificial” OR “social ...

Limiters - Publication Date: 20190101-20241231

Expanders - Apply equivalent subjects

Search modes - SmartText Searching View Results (0)

[LINK](https://web.p.ebscohost.com/ehost/search/advanced?vid=29&sid=b1537aad-8152-4f5d-894f-bb295d2669c2%40redis)

6. (“digital mental health intervention*” OR (“digital health intervention” AND “mental health”) OR “mental disorder*” OR “psychosocial intervention” OR “depressive disorder*” OR depression OR anxiet* OR “mood disorder*” OR “substance” OR “self-concept” OR “feeding and eating disorder*” OR “risk-taking” OR "suicide") AND (smart* OR “web app*” OR “mobile app*” OR “health information system*” OR “computer-assisted” OR “cell phone” OR “Internet” OR “virtual” OR “artificial” OR “social media” OR “remote consultation” OR “video gam*”) AND (“adolescents” OR "adolescent") AND (“low and middle income” OR “developing countr*” OR "LMIC*") AND (“co-design" OR "participatory”) AND "evaluation")

(“digital mental health intervention*” OR (“digital health intervention” AND “mental health”) OR “mental disorder*” OR “psychosocial intervention” OR “depressive disorder*” OR depression OR anxiet* OR “mood disorder*” OR “substance” OR “self-concept” OR “feeding and eating disorder*” OR “risk-taking” OR "suicide") AND (smart* OR “web app*” OR “mobile app*” OR “health information system*” OR “computer-assisted” OR “cell phone” OR “Internet” OR “virtual” OR “artificial” OR “social media” OR “remot ...

Limiters - Publication Date: 20190101-20241231

Expanders - Apply equivalent subjects

Search modes - SmartText Searching View Results (0)

[LINK](https://web.p.ebscohost.com/ehost/search/advanced?vid=33&sid=b1537aad-8152-4f5d-894f-bb295d2669c2%40redis)

1. **Cochrane Library**
2. (“digital health intervention*” **AND** “mental health” **AND** “adolescent*” **AND** “low and middle income”)

0 Cochrane Reviews matching (“digital health intervention*” AND “mental health” AND “adolescent*” AND “low and middle income”) in Title Abstract Keyword - with Cochrane Library publication date Between Jan 2019 and Apr 2024 (Word variations have been searched)

1. (“digital mental health intervention*” AND “adolescent*” AND “low and middle income OR LMIC*” AND (“co-design” OR "participatory") AND “evaluation”)

0 Cochrane Reviews matching (“digital mental health intervention*” AND “adolescent*” AND “low and middle income OR LMIC*” AND (“co-design” OR "participatory") AND “evaluation”) in Title Abstract Keyword - with Cochrane Library publication date Between Jan 2019 and Apr 2024 (Word variations have been searched)

1. ("digital mental health intervention*" OR (“digital health intervention” AND “mental health”) AND (“adolescen*” OR "young adult" OR “teen*”) AND ("low and middle income" OR "developing countr*" OR “LMIC*”))

68 Trials matching ("digital mental health intervention*" OR (“digital health intervention” AND “mental health”) AND (“adolescen*” OR "young adult" OR “teen*”) AND ("low and middle income" OR "developing countr*" OR “LMIC*”)) in Title Abstract Keyword - with Cochrane Library publication date Between Jan 2019 and Apr 2024 (Word variations have been searched)

1. ("digital mental health intervention*" OR ("digital health intervention" AND "mental health") AND (mhealth OR ehealth OR smart* OR "mobile app*" OR "web app*" OR "health information system*" OR "computer-assisted" OR app*) AND (adolescen* OR "young adult" OR child* OR "teen*") AND ("low and middle income" OR "developing countr*" OR LMIC*))

81 Trials matching ("digital mental health intervention*" OR ("digital health intervention" AND "mental health") AND (mhealth OR ehealth OR smart* OR "mobile app*" OR "web app*" OR "health information system*" OR "computer-assisted" OR app*) AND (adolescen* OR "young adult" OR child* OR "teen*") AND ("low and middle income" OR "developing countr*" OR LMIC*)) in Title Abstract Keyword - with Cochrane Library publication date Between Jan 2019 and Apr 2024 (Word variations have been searched)

1. ((“digital mental health intervention*” OR (“digital health intervention” AND “mental health”) OR “mental disorder*” OR “psychosocial intervention” OR “depressive disorder*” OR depression OR anxiet* OR “mood disorder*” OR “substance-related disorder*” OR “self-concept” OR “feeding and eating disorder*” OR “risk-taking” OR suicide) AND (smart* OR “web app*” OR “mobile app*” OR “health information system*” OR “computer-assisted” OR “cell phone” OR “Internet” OR “virtual” OR “artificial” OR “social media” OR “remote consultation” OR “video gam*”) AND (“adolescen*” OR “child*” OR “young adult” OR “teen*” OR “young people”) AND (“low and middle income” OR “developing countr*” OR "LMIC*") AND (“co-design" OR "participatory”) AND (“evaluation”))

0 Cochrane Reviews matching ((“digital mental health intervention*” OR (“digital health intervention” AND “mental health”) OR “mental disorder*” OR “psychosocial intervention” OR “depressive disorder*” OR depression OR anxiet* OR “mood disorder*” OR “substance-related disorder*” OR “self-concept” OR “feeding and eating disorder*” OR “risk-taking” OR suicide) AND (smart* OR “web app*” OR “mobile app*” OR “health information system*” OR “computer-assisted” OR “cell phone” OR “Internet” OR “virtual” OR “artificial” OR “social media” OR “remote consultation” OR “video gam*”) AND (“adolescen*” OR “child*” OR “young adult” OR “teen*” OR “young people”) AND (“low and middle income” OR “developing countr*” OR "LMIC*") AND (“co-design" OR "participatory”) AND (“evaluation”)) in Title Abstract Keyword - with Cochrane Library publication date Between Jan 2019 and Apr 2024 (Word variations have been searched)

1. ((("digital mental health intervention*" OR ("digital health intervention" AND "mental health") OR "mental disorder*" OR "psychosocial intervention" OR "depressive disorder*" OR depression OR anxiet* OR "mood disorder*" OR "substance" OR "self-concept" OR "feeding and eating disorder*" OR "risk-taking" OR suicide) AND (smart* OR "web app*" OR "mobile app*" OR "health information system*" OR "computer-assisted" OR "cell phone" OR "Internet" OR "virtual" OR "artificial" OR "social media" OR "remote consultation" OR "video gam*")) AND (adolescents OR "adolescent") AND ("low and middle income" OR "developing countr*" OR "LMIC*") AND (co-design OR participatory) AND "evaluation")

0 Cochrane Reviews matching ((("digital mental health intervention*" OR ("digital health intervention" AND "mental health") OR "mental disorder*" OR "psychosocial intervention" OR "depressive disorder*" OR depression OR anxiet* OR "mood disorder*" OR "substance" OR "self-concept" OR "feeding and eating disorder*" OR "risk-taking" OR suicide) AND (smart* OR "web app*" OR "mobile app*" OR "health information system*" OR "computer-assisted" OR "cell phone" OR "Internet" OR "virtual" OR "artificial" OR "social media" OR "remote consultation" OR "video gam*")) AND (adolescents OR "adolescent") AND ("low and middle income" OR "developing countr*" OR "LMIC*") AND (co-design OR participatory) AND "evaluation") in Title Abstract Keyword - with Cochrane Library publication date Between Jan 2019 and Apr 2024 (Word variations have been searched)

1. **Google Scholar**

1. (“digital health intervention*” AND “mental health” AND “adolescent*” AND “low and middle income”)

About 118 results (0.10 sec)

[LINK](https://scholar.google.co.uk/scholar?q=%28%E2%80%9Cdigital+health+intervention*%E2%80%9D+AND+%E2%80%9Cmental+health%E2%80%9D+AND+%E2%80%9Cadolescent*%E2%80%9D+AND+%E2%80%9Clow+and+middle+income%E2%80%9D%29&hl=en&as_sdt=0%2C5&as_ylo=2019&as_yhi=2024)

2. (“digital mental health intervention*” AND “adolescent*” AND “low and middle income OR LMIC*” AND (“co-design” OR "participatory") AND “evaluation”)

About 35 results (0.04 sec)

[LINK](https://scholar.google.co.uk/scholar?hl=en&as_sdt=0%2C5&as_ylo=2019&as_yhi=2024&q=%28%E2%80%9Cdigital+mental+health+intervention*%E2%80%9D+AND+%E2%80%9Cadolescent*%E2%80%9D+AND+%E2%80%9Clow+and+middle+income+OR+LMIC*%E2%80%9D+AND+%28%E2%80%9Cco-design%E2%80%9D+OR+%22participatory%22%29+AND+%E2%80%9Cevaluation%E2%80%9D%29&btnG=)

3. ("digital mental health intervention*" OR (“digital health intervention” AND “mental health”) AND (“adolescen*” OR "young adult" OR “teen*”) AND ("low and middle income" OR "developing countr*" OR “LMIC*”))

About 61 results (0.18 sec)

[LINK](https://scholar.google.co.uk/scholar?hl=en&as_sdt=0%2C5&as_ylo=2019&as_yhi=2024&q=%28%22digital+mental+health+intervention*%22+OR+%28%E2%80%9Cdigital+health+intervention%E2%80%9D+AND+%E2%80%9Cmental+health%E2%80%9D%29+AND+%28%E2%80%9Cadolescen*%E2%80%9D+OR+%22young+adult%22+OR+%E2%80%9Cteen*%E2%80%9D%29+AND+%28%22low+and+middle+income%22+OR+%22developing+countr*%22+OR+%E2%80%9CLMIC*%E2%80%9D%29%29&btnG=)

1. ("digital mental health intervention*" OR ("digital health intervention" AND "mental health") AND (mhealth OR ehealth OR smart* OR "mobile app*" OR "web app*" OR "health information system*" OR "computer-assisted" OR app*) AND (adolescen* OR "young adult" OR child* OR "teen*") AND ("low and middle income" OR "developing countr*" OR LMIC*))

About 234 results (0.04 sec) – logged first 150 only

[LINK](https://scholar.google.co.uk/scholar?q=%28%22digital+mental+health+intervention*%22+OR+%28%22digital+health+intervention%22+AND+%22mental+health%22%29+AND+%28mhealth+OR+ehealth+OR+smart*+OR+%22mobile+app*%22+OR+%22web+app*%22+OR+%22health+information+system*%22+OR+%22computer-assisted%22+OR+app*%29+AND+%28adolescen*+OR+%22young+adult%22+OR+child*+OR+%22teen*%22%29+AND+%28%22low+and+middle+income%22+OR+%22developing+countr*%22+OR+LMIC*%29%29&hl=en&as_sdt=0%2C5&as_ylo=2019&as_yhi=2024)

5. ((“digital mental health intervention*” OR (“digital health intervention” AND “mental health”) OR “mental disorder*” OR “psychosocial intervention” OR “depressive disorder*” OR depression OR anxiet* OR “mood disorder*” OR “substance-related disorder*” OR “self-concept” OR “feeding and eating disorder*” OR “risk-taking” OR suicide) AND (smart* OR “web app*” OR “mobile app*” OR “health information system*” OR “computer-assisted” OR “cell phone” OR “Internet” OR “virtual” OR “artificial” OR “social media” OR “remote consultation” OR “video gam*”) AND (“adolescen*” OR “child*” OR “young adult” OR “teen*” OR “young people”) AND (“low and middle income” OR “developing countr*” OR "LMIC*") AND (“co-design" OR "participatory”) AND (“evaluation”))

Page 15 of 142 results (0.06 sec)

[LINK](https://scholar.google.co.uk/scholar?start=140&q=((%E2%80%9Cdigital+mental+health+intervention*%E2%80%9D+OR+(%E2%80%9Cdigital+health+intervention%E2%80%9D+AND+%E2%80%9Cmental+health%E2%80%9D)+OR+%E2%80%9Cmental+disorder*%E2%80%9D+OR+%E2%80%9Cpsychosocial+intervention%E2%80%9D+OR+%E2%80%9Cdepressive+disorder*%E2%80%9D+OR+depression+OR+anxiet*+OR+%E2%80%9Cmood+disorder*%E2%80%9D+OR+%E2%80%9Csubstance-related+disorder*%E2%80%9D+OR+%E2%80%9Cself-concept%E2%80%9D+OR+%E2%80%9Cfeeding+and+eating+disorder*%E2%80%9D+OR+%E2%80%9Crisk-taking%E2%80%9D+OR+suicide)+AND+(smart*+OR+%E2%80%9Cweb+app*%E2%80%9D+OR+%E2%80%9Cmobile+app*%E2%80%9D+OR+%E2%80%9Chealth+information+system*%E2%80%9D+OR+%E2%80%9Ccomputer-assisted%E2%80%9D+OR+%E2%80%9Ccell+phone%E2%80%9D+OR+%E2%80%9CInternet%E2%80%9D+OR+%E2%80%9Cvirtual%E2%80%9D+OR+%E2%80%9Cartificial%E2%80%9D+OR+%E2%80%9Csocial+media%E2%80%9D+OR+%E2%80%9Cremote+consultation%E2%80%9D+OR+%E2%80%9Cvideo+gam*%E2%80%9D)+AND+(%E2%80%9Cadolescen*%E2%80%9D+OR+%E2%80%9Cchild*%E2%80%9D+OR+%E2%80%9Cyoung+adult%E2%80%9D+OR+%E2%80%9Cteen*%E2%80%9D+OR+%E2%80%9Cyoung+people%E2%80%9D)+AND+(%E2%80%9Clow+and+middle+income%E2%80%9D+OR+%E2%80%9Cdeveloping+countr*%E2%80%9D+OR+%22LMIC*%22)+AND+(%E2%80%9Cco-design%22+OR+%22participatory%E2%80%9D)+AND+(%E2%80%9Cevaluation%E2%80%9D))&hl=en&as_sdt=0,5&as_ylo=2019&as_yhi=2024)

6. ((("digital mental health intervention*" OR ("digital health intervention" AND "mental health") OR "mental disorder*" OR "psychosocial intervention" OR "depressive disorder*" OR depression OR anxiet* OR "mood disorder*" OR "substance" OR "self-concept" OR "feeding and eating disorder*" OR "risk-taking" OR suicide) AND (smart* OR "web app*" OR "mobile app*" OR "health information system*" OR "computer-assisted" OR "cell phone" OR "Internet" OR "virtual" OR "artificial" OR "social media" OR "remote consultation" OR "video gam*")) AND (adolescents OR "adolescent") AND ("low and middle income" OR "developing countr*" OR "LMIC*") AND (co-design OR participatory) AND "evaluation")

About 56 results (0.06 sec)

[LINK](https://scholar.google.co.uk/scholar?q=%28%28%28%22digital+mental+health+intervention*%22+OR+%28%22digital+health+intervention%22+AND+%22mental+health%22%29+OR+%22mental+disorder*%22+OR+%22psychosocial+intervention%22+OR+%22depressive+disorder*%22+OR+depression+OR+anxiet*+OR+%22mood+disorder*%22+OR+%22substance%22+OR+%22self-concept%22+OR+%22feeding+and+eating+disorder*%22+OR+%22risk-taking%22+OR+suicide%29+AND+%28smart*+OR+%22web+app*%22+OR+%22mobile+app*%22+OR+%22health+information+system*%22+OR+%22computer-assisted%22+OR+%22cell+phone%22+OR+%22Internet%22+OR+%22virtual%22+OR+%22artificial%22+OR+%22social+media%22+OR+%22remote+consultation%22+OR+%22video+gam*%22%29%29+AND+%28adolescents+OR+%22adolescent%22%29+AND+%28%22low+and+middle+income%22+OR+%22developing+countr*%22+OR+%22LMIC*%22%29+AND+%28co-design+OR+participatory%29+AND+%22evaluation%22%29&hl=en&as_sdt=0%2C5&as_ylo=2019&as_yhi=2024)

1. **IEEE Xplore**

1.

(“digital health intervention*” AND “mental health” AND “adolescent*” AND “low and middle income”)

No results foundfor

("All Metadata":(“digital health intervention*” AND "All Metadata":“mental health” AND "All Metadata":“adolescent*” AND "All Metadata":“low and middle income”))

Filters Applied:

2019 – 2024

[LINK](https://ieeexplore.ieee.org/search/searchresult.jsp?action=search&newsearch=true&matchBoolean=true&queryText=(%22All%20Metadata%22:(%E2%80%9Cdigital%20health%20intervention*%E2%80%9D%20AND%20%22All%20Metadata%22:%E2%80%9Cmental%20health%E2%80%9D%20AND%20%22All%20Metadata%22:%E2%80%9Cadolescent*%E2%80%9D%20AND%20%22All%20Metadata%22:%E2%80%9Clow%20and%20middle%20income%E2%80%9D))&ranges=2019_2024_Year)

2.

(“digital mental health intervention*” AND “adolescent*” AND “low and middle income OR LMIC*” AND (“co-design” OR "participatory") AND “evaluation”)

No results foundfor

("All Metadata":(“digital health intervention*” AND "All Metadata":“mental health” AND "All Metadata":“adolescent*” AND "All Metadata":“low and middle income”))

Filters Applied:

2019 – 2024

[LINK](https://ieeexplore.ieee.org/search/searchresult.jsp?action=search&newsearch=true&matchBoolean=true&queryText=(%22All%20Metadata%22:(%E2%80%9Cdigital%20health%20intervention*%E2%80%9D%20AND%20%22All%20Metadata%22:%E2%80%9Cmental%20health%E2%80%9D%20AND%20%22All%20Metadata%22:%E2%80%9Cadolescent*%E2%80%9D%20AND%20%22All%20Metadata%22:%E2%80%9Clow%20and%20middle%20income%E2%80%9D))&ranges=2019_2024_Year)

3.

("digital mental health intervention*" OR (“digital health intervention” AND “mental health”) AND (“adolescen*” OR "young adult" OR “teen*”) AND ("low and middle income" OR "developing countr*" OR “LMIC*”))

Showing 1-5 of 5 resultsfor (2021-2023) even though range was 2014 - 2024

("digital mental health intervention*" OR (“digital health intervention” AND “mental health”) AND (“adolescen*” OR "young adult" OR “teen*”) AND ("low and middle income" OR "developing countr*" OR “LMIC*”))

[LINK](https://ieeexplore.ieee.org/search/searchresult.jsp?newsearch=true&queryText=(%22digital%20mental%20health%20intervention*%22%20OR%20(%E2%80%9Cdigital%20health%20intervention%E2%80%9D%20AND%20%E2%80%9Cmental%20health%E2%80%9D)%20AND%20(%E2%80%9Cadolescen*%E2%80%9D%20OR%20%22young%20adult%22%20OR%20%E2%80%9Cteen*%E2%80%9D)%20AND%20(%22low%20and%20middle%20income%22%20OR%20%22developing%20countr*%22%20OR%20%E2%80%9CLMIC*%E2%80%9D)))

4.

("digital mental health intervention*" OR ("digital health intervention" AND "mental health") AND (mhealth OR ehealth OR smart* OR "mobile app*" OR "web app*" OR "health information system*" OR "computer-assisted" OR app*) AND (adolescen* OR "young adult" OR child* OR "teen*") AND ("low and middle income" OR "developing countr*" OR LMIC*))

No results foundfor

("All Metadata":("digital mental health intervention*" OR "All Metadata":("digital health intervention" AND "All Metadata":"mental health")) AND ("All Metadata":(mhealth OR "All Metadata":ehealth OR "All Metadata":smart* OR "All Metadata":"mobile app*" OR "All Metadata":"web app*" OR "All Metadata":"health information system*" OR "All Metadata":"computer-assisted" OR "All Metadata":app*)) AND ("All Metadata":(adolescen* OR "All Metadata":"young adult" OR "All Metadata":child* OR "All Metadata":"teen*")) AND ("All Metadata":("low and middle income" OR "All Metadata":"developing countr*" OR "All Metadata":LMIC*)))

Filters Applied:

2019 - 2024

[LINK](https://ieeexplore.ieee.org/search/searchresult.jsp?action=search&newsearch=true&matchBoolean=true&queryText=(%22All%20Metadata%22:(%22digital%20mental%20health%20intervention*%22%20OR%20%22All%20Metadata%22:(%22digital%20health%20intervention%22%20AND%20%22All%20Metadata%22:%22mental%20health%22))%20AND%20(%22All%20Metadata%22:(mhealth%20OR%20%22All%20Metadata%22:ehealth%20OR%20%22All%20Metadata%22:smart*%20OR%20%22All%20Metadata%22:%22mobile%20app*%22%20OR%20%22All%20Metadata%22:%22web%20app*%22%20OR%20%22All%20Metadata%22:%22health%20information%20system*%22%20OR%20%22All%20Metadata%22:%22computer-assisted%22%20OR%20%22All%20Metadata%22:app*))%20AND%20(%22All%20Metadata%22:(adolescen*%20OR%20%22All%20Metadata%22:%22young%20adult%22%20OR%20%22All%20Metadata%22:child*%20OR%20%22All%20Metadata%22:%22teen*%22))%20AND%20(%22All%20Metadata%22:(%22low%20and%20middle%20income%22%20OR%20%22All%20Metadata%22:%22developing%20countr*%22%20OR%20%22All%20Metadata%22:LMIC*)))&ranges=2019_2024_Year)

5.

((“digital mental health intervention*” OR (“digital health intervention” AND “mental health”) AND (“mental disorder*” OR “psychosocial intervention” OR “depressive disorder*” OR depression OR anxiet* OR “mood disorder*” OR “substance-related disorder*” OR “self-concept” OR “feeding and eating disorder*” OR “risk-taking” OR suicide) AND (smart* OR “web app*” OR “mobile app*” OR “health information system*” OR “computer-assisted” OR “cell phone” OR “Internet” OR “virtual” OR “artificial” OR “social media” OR “remote consultation” OR “video gam*”) AND (“adolescen*” OR “child*” OR “young adult” OR “teen*” OR “young people”) AND (“low and middle income” OR “developing countr*” OR "LMIC*") AND (“co-design" OR "participatory”) AND (“evaluation”))

No results foundfor

("All Metadata":((“digital mental health intervention*” OR "All Metadata":(“digital health intervention” AND "All Metadata":“mental health”) OR "All Metadata":“mental disorder*” OR "All Metadata":“psychosocial intervention” OR "All Metadata":“depressive disorder*” OR "All Metadata":depression OR "All Metadata":anxiet* OR "All Metadata":“mood disorder*” OR "All Metadata":“substance-related disorder*” OR "All Metadata":“self-concept” OR "All Metadata":“feeding and eating disorder*” OR "All Metadata":“risk-taking” OR "All Metadata":suicide)) AND ("All Metadata":(smart* OR "All Metadata":“web app*” OR "All Metadata":“mobile app*” OR "All Metadata":“health information system*” OR "All Metadata":“computer-assisted” OR "All Metadata":“cell phone” OR "All Metadata":“Internet” OR "All Metadata":“virtual” OR "All Metadata":“artificial” OR "All Metadata":“social media” OR "All Metadata":“remote consultation” OR "All Metadata":“video gam*”)) AND ("All Metadata":(“adolescen*” OR "All Metadata":“child*” OR "All Metadata":“young adult” OR "All Metadata":“teen*” OR "All Metadata":“young people”)) AND ("All Metadata":(“low and middle income” OR "All Metadata":“developing countr*” OR "All Metadata":"LMIC*")) AND ("All Metadata":(“co-design" OR "All Metadata":"participatory”) AND "All Metadata":(“evaluation”)))

Filters Applied:

2019 – 2024

LINK: https://ieeexplore.ieee.org/search/searchresult.jsp?action=search&newsearch=true&matchBoolean=true&queryText=(%22All%20Metadata%22:((%E2%80%9Cdigital%20mental%20health%20intervention*%E2%80%9D%20OR%20%22All%20Metadata%22:(%E2%80%9Cdigital%20health%20intervention%E2%80%9D%20AND%20%22All%20Metadata%22:%E2%80%9Cmental%20health%E2%80%9D)%20OR%20%22All%20Metadata%22:%E2%80%9Cmental%20disorder*%E2%80%9D%20OR%20%22All%20Metadata%22:%E2%80%9Cpsychosocial%20intervention%E2%80%9D%20OR%20%22All%20Metadata%22:%E2%80%9Cdepressive%20disorder*%E2%80%9D%20OR%20%22All%20Metadata%22:depression%20OR%20%22All%20Metadata%22:anxiet*%20OR%20%22All%20Metadata%22:%E2%80%9Cmood%20disorder*%E2%80%9D%20OR%20%22All%20Metadata%22:%E2%80%9Csubstance-related%20disorder*%E2%80%9D%20OR%20%22All%20Metadata%22:%E2%80%9Cself-concept%E2%80%9D%20OR%20%22All%20Metadata%22:%E2%80%9Cfeeding%20and%20eating%20disorder*%E2%80%9D%20OR%20%22All%20Metadata%22:%E2%80%9Crisk-taking%E2%80%9D%20OR%20%22All%20Metadata%22:suicide))%20AND%20(%22All%20Metadata%22:(smart*%20OR%20%22All%20Metadata%22:%E2%80%9Cweb%20app*%E2%80%9D%20OR%20%22All%20Metadata%22:%E2%80%9Cmobile%20app*%E2%80%9D%20OR%20%22All%20Metadata%22:%E2%80%9Chealth%20information%20system*%E2%80%9D%20OR%20%22All%20Metadata%22:%E2%80%9Ccomputer-assisted%E2%80%9D%20OR%20%22All%20Metadata%22:%E2%80%9Ccell%20phone%E2%80%9D%20OR%20%22All%20Metadata%22:%E2%80%9CInternet%E2%80%9D%20OR%20%22All%20Metadata%22:%E2%80%9Cvirtual%E2%80%9D%20OR%20%22All%20Metadata%22:%E2%80%9Cartificial%E2%80%9D%20OR%20%22All%20Metadata%22:%E2%80%9Csocial%20media%E2%80%9D%20OR%20%22All%20Metadata%22:%E2%80%9Cremote%20consultation%E2%80%9D%20OR%20%22All%20Metadata%22:%E2%80%9Cvideo%20gam*%E2%80%9D))%20AND%20(%22All%20Metadata%22:(%E2%80%9Cadolescen*%E2%80%9D%20OR%20%22All%20Metadata%22:%E2%80%9Cchild*%E2%80%9D%20OR%20%22All%20Metadata%22:%E2%80%9Cyoung%20adult%E2%80%9D%20OR%20%22All%20Metadata%22:%E2%80%9Cteen*%E2%80%9D%20OR%20%22All%20Metadata%22:%E2%80%9Cyoung%20people%E2%80%9D))%20AND%20(%22All%20Metadata%22:(%E2%80%9Clow%20and%20middle%20income%E2%80%9D%20OR%20%22All%20Metadata%22:%E2%80%9Cdeveloping%20countr*%E2%80%9D%20OR%20%22All%20Metadata%22:%22LMIC*%22))%20AND%20(%22All%20Metadata%22:(%E2%80%9Cco-design%22%20OR%20%22All%20Metadata%22:%22participatory%E2%80%9D)%20AND%20%22All%20Metadata%22:(%E2%80%9Cevaluation%E2%80%9D)))&ranges=2019_2024_Year

6.

((("digital mental health intervention*" OR ("digital health intervention" AND "mental health") OR "mental disorder*" OR "psychosocial intervention" OR "depressive disorder*" OR depression OR anxiet* OR "mood disorder*" OR "substance" OR "self-concept" OR "feeding and eating disorder*" OR "risk-taking" OR suicide) AND (smart* OR "web app*" OR "mobile app*" OR "health information system*" OR "computer-assisted" OR "cell phone" OR "Internet" OR "virtual" OR "artificial" OR "social media" OR "remote consultation" OR "video gam*")) AND (adolescents OR "adolescent") AND ("low and middle income" OR "developing countr*" OR "LMIC*") AND (co-design OR participatory) AND "evaluation")

No results foundfor

("All Metadata":((("digital mental health intervention*" OR "All Metadata":("digital health intervention" AND "All Metadata":"mental health") OR "All Metadata":"mental disorder*" OR "All Metadata":"psychosocial intervention" OR "All Metadata":"depressive disorder*" OR "All Metadata":depression OR "All Metadata":anxiet* OR "All Metadata":"mood disorder*" OR "All Metadata":"substance" OR "All Metadata":"self-concept" OR "All Metadata":"feeding and eating disorder*" OR "All Metadata":"risk-taking" OR "All Metadata":suicide)) AND ("All Metadata":(smart* OR "All Metadata":"web app*" OR "All Metadata":"mobile app*" OR "All Metadata":"health information system*" OR "All Metadata":"computer-assisted" OR "All Metadata":"cell phone" OR "All Metadata":"Internet" OR "All Metadata":"virtual" OR "All Metadata":"artificial" OR "All Metadata":"social media" OR "All Metadata":"remote consultation" OR "All Metadata":"video gam*"))) AND ("All Metadata":(adolescents OR "All Metadata":"adolescent") AND "All Metadata":("low and middle income" OR "All Metadata":"developing countr*" OR "All Metadata":"LMIC*")) AND ("All Metadata":(co-design OR "All Metadata":participatory) AND "All Metadata":"evaluation"))

Filters Applied:

2019 – 2024

[LINK](https://ieeexplore.ieee.org/search/searchresult.jsp?action=search&newsearch=true&matchBoolean=true&queryText=(%22All%20Metadata%22:(((%22digital%20mental%20health%20intervention*%22%20OR%20%22All%20Metadata%22:(%22digital%20health%20intervention%22%20AND%20%22All%20Metadata%22:%22mental%20health%22)%20OR%20%22All%20Metadata%22:%22mental%20disorder*%22%20OR%20%22All%20Metadata%22:%22psychosocial%20intervention%22%20OR%20%22All%20Metadata%22:%22depressive%20disorder*%22%20OR%20%22All%20Metadata%22:depression%20OR%20%22All%20Metadata%22:anxiet*%20OR%20%22All%20Metadata%22:%22mood%20disorder*%22%20OR%20%22All%20Metadata%22:%22substance%22%20OR%20%22All%20Metadata%22:%22self-concept%22%20OR%20%22All%20Metadata%22:%22feeding%20and%20eating%20disorder*%22%20OR%20%22All%20Metadata%22:%22risk-taking%22%20OR%20%22All%20Metadata%22:suicide))%20AND%20(%22All%20Metadata%22:(smart*%20OR%20%22All%20Metadata%22:%22web%20app*%22%20OR%20%22All%20Metadata%22:%22mobile%20app*%22%20OR%20%22All%20Metadata%22:%22health%20information%20system*%22%20OR%20%22All%20Metadata%22:%22computer-assisted%22%20OR%20%22All%20Metadata%22:%22cell%20phone%22%20OR%20%22All%20Metadata%22:%22Internet%22%20OR%20%22All%20Metadata%22:%22virtual%22%20OR%20%22All%20Metadata%22:%22artificial%22%20OR%20%22All%20Metadata%22:%22social%20media%22%20OR%20%22All%20Metadata%22:%22remote%20consultation%22%20OR%20%22All%20Metadata%22:%22video%20gam*%22)))%20AND%20(%22All%20Metadata%22:(adolescents%20OR%20%22All%20Metadata%22:%22adolescent%22)%20AND%20%22All%20Metadata%22:(%22low%20and%20middle%20income%22%20OR%20%22All%20Metadata%22:%22developing%20countr*%22%20OR%20%22All%20Metadata%22:%22LMIC*%22))%20AND%20(%22All%20Metadata%22:(co-design%20OR%20%22All%20Metadata%22:participatory)%20AND%20%22All%20Metadata%22:%22evaluation%22))&ranges=2019_2024_Year)

1. **Pro Quest**
2. (“digital health intervention*” **AND** “mental health” **AND** “adolescent*” **AND** “low and middle income”)

| [(“digital health intervention*” AND “mental health” AND “adolescent*” AND “low and middle income”)](https://www.proquest.com/recentsearches.recentsearchtabview.recentsearchesgridview.scrolledrecentsearchlist.checkdbssearchlink:rerunsearch/BF92118315B14830PQ/None/$N?t:ac=RecentSearches)Limits applied  Databases:  All databases searched  [View list](https://www.proquest.com/recentsearches.recentsearchtabview.recentsearchesgridview.scrolledrecentsearchlist.displaydatabaselist:loadlist/2/displayDatabaseList?t:ac=RecentSearches)  *These databases are searched for part of your query.*  Narrowed by:  Entered date:  2019-03-24 - 2024-03-24 | 27 databases | [**185**](https://www.proquest.com/recentsearches.recentsearchtabview.recentsearchesgridview.scrolledrecentsearchlist.checkdbssearchlink_0:rerunsearch/BF92118315B14830PQ/None/$N?t:ac=RecentSearches) |
| --- | --- | --- |

2.

(“digital mental health intervention*” AND “adolescent*” AND “low and middle income OR LMIC*” AND (“co-design” OR "participatory") AND “evaluation”)

Your search for (“digital mental health intervention*” AND “adolescent*” AND “low and middle income OR LMIC*” AND (“co-design” OR "participatory") AND “evaluation”) found 0 results. use the following link to help refine your search termSearch tips

3.

("digital mental health intervention*" OR (“digital health intervention” AND “mental health”) AND (“adolescen*” OR "young adult" OR “teen*”) AND ("low and middle income" OR "developing countr*" OR “LMIC*”))

| [("digital mental health intervention*" OR (“digital health intervention” AND “mental health”) AND (“adolescen*” OR "young adult" OR “teen*”) AND ("low and middle income" OR ("developing countries" OR "developing country") OR “LMIC*”))](https://www.proquest.com/recentsearches.recentsearchtabview.recentsearchesgridview.scrolledrecentsearchlist.checkdbssearchlink:rerunsearch/6632EF3EED794E3CPQ/None/$N?t:ac=RecentSearches)Limits applied  Databases:  All databases searched  [View list](https://www.proquest.com/recentsearches.recentsearchtabview.recentsearchesgridview.scrolledrecentsearchlist.displaydatabaselist:loadlist/5/displayDatabaseList?t:ac=RecentSearches)  *These databases are searched for part of your query.*  Narrowed by:  Entered date:  2019-03-24 - 2024-03-24 | 27 databases | [**1,589**](https://www.proquest.com/recentsearches.recentsearchtabview.recentsearchesgridview.scrolledrecentsearchlist.checkdbssearchlink_0:rerunsearch/6632EF3EED794E3CPQ/None/$N?t:ac=RecentSearches) |
| --- | --- | --- |

4. ("digital mental health intervention*" OR ("digital health intervention" AND "mental health") AND (mhealth OR ehealth OR smart* OR "mobile app*" OR "web app*" OR "health information system*" OR "computer-assisted" OR app*) AND (adolescen* OR "young adult" OR child* OR "teen*") AND ("low and middle income" OR "developing countr*" OR LMIC*))

| [("digital mental health intervention*" OR ("digital health intervention" AND "mental health") AND (mhealth OR ehealth OR smart* OR ("mobile app" OR "mobile application" OR "mobile applications" OR "mobile apps") OR ("web app" OR "web application" OR "web applications" OR "web apps") OR "health information system*" OR "computer-assisted" OR app*) AND (adolescen* OR "young adult" OR child* OR "teen*") AND ("low and middle income" OR ("developing countries" OR "developing country") OR LMIC*))](https://www.proquest.com/recentsearches.recentsearchtabview.recentsearchesgridview.scrolledrecentsearchlist.checkdbssearchlink:rerunsearch/94E41D9455D8462FPQ/None/$N?t:ac=RecentSearches)Limits applied  Databases:  All databases searched  [View list](https://www.proquest.com/recentsearches.recentsearchtabview.recentsearchesgridview.scrolledrecentsearchlist.displaydatabaselist:loadlist/6/displayDatabaseList?t:ac=RecentSearches)  *These databases are searched for part of your query.*  Narrowed by:  Entered date:  2019-03-24 - 2024-03-24 | 27 databases | [**1,610**](https://www.proquest.com/recentsearches.recentsearchtabview.recentsearchesgridview.scrolledrecentsearchlist.checkdbssearchlink_0:rerunsearch/94E41D9455D8462FPQ/None/$N?t:ac=RecentSearches) |
| --- | --- | --- |

5. ((“digital mental health intervention*” OR (“digital health intervention” AND “mental health”) OR “mental disorder*” OR “psychosocial intervention” OR “depressive disorder*” OR depression OR anxiet* OR “mood disorder*” OR “substance-related disorder*” OR “self-concept” OR “feeding and eating disorder*” OR “risk-taking” OR suicide) AND (smart* OR “web app*” OR “mobile app*” OR “health information system*” OR “computer-assisted” OR “cell phone” OR “Internet” OR “virtual” OR “artificial” OR “social media” OR “remote consultation” OR “video gam*”) AND (“adolescen*” OR “child*” OR “young adult” OR “teen*” OR “young people”) AND (“low and middle income” OR “developing countr*” OR "LMIC*") AND (“co-design" OR "participatory”) AND (“evaluation”))

| [((“digital mental health intervention*” OR (“digital health intervention” AND “mental health”) OR ("mental disorder" OR "mental disorders") OR “psychosocial intervention” OR ("depressive disorder") OR depression OR anxiet* OR ("mood disorder" OR "mood disorders") OR “substance-related disorder*” OR “self-concept” OR “feeding and eating disorder*” OR “risk-taking” OR suicide) AND (smart* OR ("web app" OR "web application" OR "web applications" OR "web apps") OR ("mobile app" OR "mobile application" OR "mobile applications" OR "mobile apps") OR “health information system*” OR “computer-assisted” OR “cell phone” OR “Internet” OR “virtual” OR “artificial” OR “social media” OR “remote consultation” OR ("video gambling" OR "video game" OR "video gamer" OR "video gamers" OR "video games" OR "video gaming")) AND (“adolescen*” OR “child*” OR “young adult” OR “teen*” OR “young people”) AND (“low and middle income” OR ("developing countries" OR "developing country") OR "LMIC*") AND (“co-design" OR "participatory”) AND (“evaluation”))](https://www.proquest.com/recentsearches.recentsearchtabview.recentsearchesgridview.scrolledrecentsearchlist.checkdbssearchlink:rerunsearch/335BA29989934701PQ/None/$N?t:ac=RecentSearches)Limits applied  Databases:  All databases searched  [View list](https://www.proquest.com/recentsearches.recentsearchtabview.recentsearchesgridview.scrolledrecentsearchlist.displaydatabaselist:loadlist/7/displayDatabaseList?t:ac=RecentSearches)  *These databases are searched for part of your query.*  Narrowed by:  Entered date:  2019-03-24 - 2024-03-24 | 27 databases | [**8,911**](https://www.proquest.com/recentsearches.recentsearchtabview.recentsearchesgridview.scrolledrecentsearchlist.checkdbssearchlink_0:rerunsearch/335BA29989934701PQ/None/$N?t:ac=RecentSearches) |
| --- | --- | --- |

6. ((("digital mental health intervention*" OR ("digital health intervention" AND "mental health") OR "mental disorder*" OR "psychosocial intervention" OR "depressive disorder*" OR depression OR anxiet* OR "mood disorder*" OR "substance" OR "self-concept" OR "feeding and eating disorder*" OR "risk-taking" OR suicide) AND (smart* OR "web app*" OR "mobile app*" OR "health information system*" OR "computer-assisted" OR "cell phone" OR "Internet" OR "virtual" OR "artificial" OR "social media" OR "remote consultation" OR "video gam*")) AND (adolescents OR "adolescent") AND ("low and middle income" OR "developing countr*" OR "LMIC*") AND (co-design OR participatory) AND "evaluation")

| [((("digital mental health intervention*" OR ("digital health intervention" AND "mental health") OR ("mental disorder" OR "mental disorders") OR "psychosocial intervention" OR ("depressive disorder") OR depression OR anxiet* OR ("mood disorder" OR "mood disorders") OR "substance" OR "self-concept" OR "feeding and eating disorder*" OR "risk-taking" OR suicide) AND (smart* OR ("web app" OR "web application" OR "web applications" OR "web apps") OR ("mobile app" OR "mobile application" OR "mobile applications" OR "mobile apps") OR "health information system*" OR "computer-assisted" OR "cell phone" OR "Internet" OR "virtual" OR "artificial" OR "social media" OR "remote consultation" OR ("video gambling" OR "video game" OR "video gamer" OR "video gamers" OR "video games" OR "video gaming"))) AND (adolescents OR "adolescent") AND ("low and middle income" OR ("developing countries" OR "developing country") OR "LMIC*") AND (co-design OR participatory) AND "evaluation")](https://www.proquest.com/recentsearches.recentsearchtabview.recentsearchesgridview.scrolledrecentsearchlist.checkdbssearchlink:rerunsearch/3BC21C1AF88F4E24PQ/None/$N?t:ac=RecentSearches)Limits applied  Databases:  All databases searched  [View list](https://www.proquest.com/recentsearches.recentsearchtabview.recentsearchesgridview.scrolledrecentsearchlist.displaydatabaselist:loadlist/8/displayDatabaseList?t:ac=RecentSearches)  *These databases are searched for part of your query.*  Narrowed by:  Entered date:  2019-03-24 - 2024-03-24 | 27 databases | [**6,700**](https://www.proquest.com/recentsearches.recentsearchtabview.recentsearchesgridview.scrolledrecentsearchlist.checkdbssearchlink_0:rerunsearch/3BC21C1AF88F4E24PQ/None/$N?t:ac=RecentSearches) |
| --- | --- | --- |

1. **PubMed (NLM)**

1.

(“digital health intervention*” AND “mental health” AND “adolescent*” AND “low and middle income”)

= 2 results

[LINK](https://pubmed.ncbi.nlm.nih.gov/?term=%28%22digital+health+intervention*%22+AND+%22mental+health%22+AND+%22adolescent*%22+AND+%22low+and+middle+income%22%29&filter=dates.2019-2024)

2.

(“digital mental health intervention*” AND “adolescent*” AND “low and middle income OR LMIC*” AND (“co-design” OR "participatory") AND “evaluation”)

= 0

[LINK](https://pubmed.ncbi.nlm.nih.gov/?term=%28%22digital+mental+health+intervention*%22+AND+%22adolescent*%22+AND+%22low+and+middle+income+OR+LMIC*%22+AND+%28%22co-design%22+OR+%22participatory%22%29+AND+%22evaluation%22%29&filter=datesearch.y_5)

3. ("digital mental health intervention*" OR (“digital health intervention” AND “mental health”) AND (“adolescen*” OR "young adult" OR “teen*”) AND ("low and middle income" OR "developing countr*" OR “LMIC*”))

= 4

[LINK](https://pubmed.ncbi.nlm.nih.gov/?term=%28%22digital+mental+health+intervention*%22+OR+%28%22digital+health+intervention%22+AND+%22mental+health%22%29+AND+%28%22adolescen*%22+OR+%22young+adult%22+OR+%22teen*%22%29+AND+%28%22low+and+middle+income%22+OR+%22developing+countr*%22+OR+%22LMIC*%22%29%29&filter=datesearch.y_5)

4. ("digital mental health intervention*" OR ("digital health intervention" AND "mental health") AND (mhealth OR ehealth OR smart* OR "mobile app*" OR "web app*" OR "health information system*" OR "computer-assisted" OR app*) AND (adolescen* OR "young adult" OR child* OR "teen*") AND ("low and middle income" OR "developing countr*" OR LMIC*))

= 3

[LINK](https://pubmed.ncbi.nlm.nih.gov/?term=%28%22digital+mental+health+intervention*%22+OR+%28%22digital+health+intervention%22+AND+%22mental+health%22%29+AND+%28mhealth+OR+ehealth+OR+smart*+OR+%22mobile+app*%22+OR+%22web+app*%22+OR+%22health+information+system*%22+OR+%22computer-assisted%22+OR+app*%29+AND+%28adolescen*+OR+%22young+adult%22+OR+child*+OR+%22teen*%22%29+AND+%28%22low+and+middle+income%22+OR+%22developing+countr*%22+OR+LMIC*%29%29&filter=datesearch.y_5)

5. ((“digital mental health intervention*” OR (“digital health intervention” AND “mental health”) OR “mental disorder*” OR “psychosocial intervention” OR “depressive disorder*” OR depression OR anxiet* OR “mood disorder*” OR “substance-related disorder*” OR “self-concept” OR “feeding and eating disorder*” OR “risk-taking” OR suicide) AND (smart* OR “web app*” OR “mobile app*” OR “health information system*” OR “computer-assisted” OR “cell phone” OR “Internet” OR “virtual” OR “artificial” OR “social media” OR “remote consultation” OR “video gam*”) AND (“adolescen*” OR “child*” OR “young adult” OR “teen*” OR “young people”) AND (“low and middle income” OR “developing countr*” OR "LMIC*") AND (“co-design" OR "participatory”) AND (“evaluation”))

= 0

[LINK](https://pubmed.ncbi.nlm.nih.gov/?term=%28%28%22digital+mental+health+intervention*%22+OR+%28%22digital+health+intervention%22+AND+%22mental+health%22%29+OR+%22mental+disorder*%22+OR+%22psychosocial+intervention%22+OR+%22depressive+disorder*%22+OR+depression+OR+anxiet*+OR+%22mood+disorder*%22+OR+%22substance-related+disorder*%22+OR+%22self-concept%22+OR+%22feeding+and+eating+disorder*%22+OR+%22risk-taking%22+OR+suicide%29+AND+%28smart*+OR+%22web+app*%22+OR+%22mobile+app*%22+OR+%22health+information+system*%22+OR+%22computer-assisted%22+OR+%22cell+phone%22+OR+%22Internet%22+OR+%22virtual%22+OR+%22artificial%22+OR+%22social+media%22+OR+%22remote+consultation%22+OR+%22video+gam*%22%29+AND+%28%22adolescen*%22+OR+%22child*%22+OR+%22young+adult%22+OR+%22teen*%22+OR+%22young+people%22%29+AND+%28%22low+and+middle+income%22+OR+%22developing+countr*%22+OR+%22LMIC*%22%29+AND+%28%22co-design%22+OR+%22participatory%22%29+AND+%28%22evaluation%22%29%29&filter=datesearch.y_5)

6. 2((("digital mental health intervention*" OR ("digital health intervention" AND "mental health") OR "mental disorder*" OR "psychosocial intervention" OR "depressive disorder*" OR depression OR anxiet* OR "mood disorder*" OR "substance" OR "self-concept" OR "feeding and eating disorder*" OR "risk-taking" OR suicide) AND (smart* OR "web app*" OR "mobile app*" OR "health information system*" OR "computer-assisted" OR "cell phone" OR "Internet" OR "virtual" OR "artificial" OR "social media" OR "remote consultation" OR "video gam*")) AND (adolescents OR "adolescent") AND ("low and middle income" OR "developing countr*" OR "LMIC*") AND (co-design OR participatory) AND "evaluation")

= 0

[LINK](https://pubmed.ncbi.nlm.nih.gov/?term=%28%28%28%22digital+mental+health+intervention*%22+OR+%28%22digital+health+intervention%22+AND+%22mental+health%22%29+OR+%22mental+disorder*%22+OR+%22psychosocial+intervention%22+OR+%22depressive+disorder*%22+OR+depression+OR+anxiet*+OR+%22mood+disorder*%22+OR+%22substance%22+OR+%22self-concept%22+OR+%22feeding+and+eating+disorder*%22+OR+%22risk-taking%22+OR+suicide%29+AND+%28smart*+OR+%22web+app*%22+OR+%22mobile+app*%22+OR+%22health+information+system*%22+OR+%22computer-assisted%22+OR+%22cell+phone%22+OR+%22Internet%22+OR+%22virtual%22+OR+%22artificial%22+OR+%22social+media%22+OR+%22remote+consultation%22+OR+%22video+gam*%22%29%29+AND+%28adolescents+OR+%22adolescent%22%29+AND+%28%22low+and+middle+income%22+OR+%22developing+countr*%22+OR+%22LMIC*%22%29+AND+%28co-design+OR+participatory%29+AND+%22evaluation%22%29&filter=datesearch.y_5)

1. **ScienceDirect**
2. ("digital health intervention" AND "mental health" AND "adolescent" AND "low and middle income")

=20

[LINK](https://www.sciencedirect.com/search?qs=%28%22digital%20health%20intervention%22%20AND%20%22mental%20health%22%20AND%20%22adolescent%22%20AND%20%22low%20and%20middle%20income%22%29&date=2019-2024)

1. ("digital mental health intervention" AND "adolescent" AND ("low and middle income" OR "LMICs") AND ("co-design" OR "participatory") AND "evaluation")

=1

[LINK](https://www.sciencedirect.com/search?date=2019-2024&qs=%28%22digital%20mental%20health%20intervention%22%20AND%20%22adolescent%22%20AND%20%28%22low%20and%20middle%20income%22%20OR%20%22LMICs%22%29%20AND%20%28%22co-design%22%20OR%20%22participatory%22%29%20AND%20%22evaluation%22%29)

1. (("digital mental health intervention" OR ("digital health intervention" AND "mental health")) AND ("adolescent" OR "young adult" OR "teen") AND ("low and middle income" OR "developing countr" OR "LMIC"))

= 34

[LINK](https://www.sciencedirect.com/search?date=2019-2024&qs=%28%28%22digital%20mental%20health%20intervention%22%20OR%20%28%22digital%20health%20intervention%22%20AND%20%22mental%20health%22%29%29%20AND%20%28%22adolescent%22%20OR%20%22young%20adult%22%20OR%20%22teen%22%29%20AND%20%28%22low%20and%20middle%20income%22%20OR%20%22developing%20countr%22%20OR%20%22LMIC%22%29%29&show=100)

("digital mental health intervention*" OR ("digital health intervention" **AND** "mental health") **AND** (mhealth OR ehealth OR smart* OR "mobile app*" OR "web app*" OR "health information system*" OR "computer-assisted" OR app*) **AND** (adolescen* OR "young adult" OR child* OR "teen*") **AND** ("low and middle income" OR "developing countr*" OR LMIC*))

Use fewer boolean connectors (max 8 per field)

((“digital mental health intervention*” OR (“digital health intervention” AND “mental health”) OR “mental disorder*” OR “psychosocial intervention” OR “depressive disorder*” OR depression OR anxiet* OR “mood disorder*” OR “substance-related disorder*” OR “self-concept” OR “feeding and eating disorder*” OR “risk-taking” OR suicide) **AND** (smart* OR “web app*” OR “mobile app*” OR “health information system*” OR “computer-assisted” OR “cell phone” OR “Internet” OR “virtual” OR “artificial” OR “social media” OR “remote consultation” OR “video gam*”) **AND** (“adolescen*” OR “child*” OR “young adult” OR “teen*” OR “young people”) **AND** (“low and middle income” OR “developing countr*” OR "LMIC*") **AND** (“co-design" OR "participatory”) **AND** (“evaluation”))

Use fewer boolean connectors (max 8 per field)

((("digital mental health intervention*" OR ("digital health intervention" **AND** "mental health") OR "mental disorder*" OR "psychosocial intervention" OR "depressive disorder*" OR depression OR anxiet* OR "mood disorder*" OR "substance" OR "self-concept" OR "feeding and eating disorder*" OR "risk-taking" OR suicide) **AND** (smart* OR "web app*" OR "mobile app*" OR "health information system*" OR "computer-assisted" OR "cell phone" OR "Internet" OR "virtual" OR "artificial" OR "social media" OR "remote consultation" OR "video gam*")) **AND** (adolescents OR "adolescent") AND ("low and middle income" OR "developing countr*" OR "LMIC*") **AND** (co-design OR participatory) AND "evaluation")

Use fewer boolean connectors (max 8 per field)

1. **Scopus**

1. (“digital health intervention*” AND “mental health” AND “adolescent*” AND “low and middle income”)

TITLE-ABS-KEY ( ( "digital health intervention*" AND "mental health" AND "adolescent*" AND "low and middle income" ) ) AND PUBYEAR > 2019 AND PUBYEAR < 2024

= 2

[LINK](https://www-scopus-com.proxy.lib.strath.ac.uk/results/results.uri?sort=plf-f&src=s&sid=44bf5a4e2aefd70b7e71a39810864a10&sot=a&sdt=a&sl=157&s=TITLE-ABS-KEY%28%28%22digital+health+intervention*%22+AND+%22mental+health%22+AND+%22adolescent*%22+AND+%22low+and+middle+income%22%29%29+AND+PUBYEAR+%26gt%3B+2019+AND+PUBYEAR+%26lt%3B+2024&origin=searchadvanced&editSaveSearch=&txGid=a3655a99e760799f5c82042b5b2af9b5&sessionSearchId=44bf5a4e2aefd70b7e71a39810864a10&limit=10)

2.

(“digital mental health intervention*” AND “adolescent*” AND “low and middle income OR LMIC*” AND (“co-design” OR "participatory") AND “evaluation”)

TITLE-ABS-KEY ( ( "digital mental health intervention*" AND "adolescent*" AND "low and middle income OR LMIC*" AND ( "co-design" OR "participatory" ) AND "evaluation" ) ) AND PUBYEAR > 2019 AND PUBYEAR < 2024

= 0

[LINK](https://www-scopus-com.proxy.lib.strath.ac.uk/results/results.uri?sort=plf-f&src=s&sid=dec38b442cf490f6fb852a18673d21ec&sot=a&sdt=a&sl=207&s=TITLE-ABS-KEY%28%28%22digital+mental+health+intervention*%22+AND+%22adolescent*%22+AND+%22low+and+middle+income+OR+LMIC*%22+AND+%28%22co-design%22+OR+%22participatory%22%29+AND+%22evaluation%22%29%29+AND+PUBYEAR+%26gt%3B+2019+AND+PUBYEAR+%26lt%3B+2024&origin=searchadvanced&editSaveSearch=&txGid=750578fcef753f87e509e7609df65dc8&sessionSearchId=dec38b442cf490f6fb852a18673d21ec&limit=10)

3. ("digital mental health intervention*" OR (“digital health intervention” AND “mental health”) AND (“adolescen*” OR "young adult" OR “teen*”) AND ("low and middle income" OR "developing countr*" OR “LMIC*”))

TITLE-ABS-KEY ( ( "digital mental health intervention*" OR ( "digital health intervention" AND "mental health" ) AND ( "adolescen*" OR "young adult" OR "teen*" ) AND ( "low and middle income" OR "developing countr*" OR "LMIC*" ) ) ) AND PUBYEAR > 2019 AND PUBYEAR < 2024

= 4

[LINK](https://www-scopus-com.proxy.lib.strath.ac.uk/results/results.uri?sort=plf-f&src=s&sid=c8a3630bf96245ae27f073baeff84ae7&sot=a&sdt=a&sl=265&s=TITLE-ABS-KEY%28%28%22digital+mental+health+intervention*%22+OR+%28%22digital+health+intervention%22+AND+%22mental+health%22%29+AND+%28%22adolescen*%22+OR+%22young+adult%22+OR+%22teen*%22%29+AND+%28%22low+and+middle+income%22+OR+%22developing+countr*%22+OR+%22LMIC*%22%29%29%29+AND+PUBYEAR+%26gt%3B+2019+AND+PUBYEAR+%26lt%3B+2024&origin=searchadvanced&editSaveSearch=&txGid=5d28f00041d38dd02db8087cde91eff8&sessionSearchId=c8a3630bf96245ae27f073baeff84ae7&limit=10)

4. ("digital mental health intervention*" OR ("digital health intervention" AND "mental health") AND (mhealth OR ehealth OR smart* OR "mobile app*" OR "web app*" OR "health information system*" OR "computer-assisted" OR app*) AND (adolescen* OR "young adult" OR child* OR "teen*") AND ("low and middle income" OR "developing countr*" OR LMIC*))

TITLE-ABS-KEY ( ( "digital mental health intervention*" OR ( "digital health intervention" AND "mental health" ) AND ( mhealth OR ehealth OR smart* OR "mobile app*" OR "web app*" OR "health information system*" OR "computer-assisted" OR app* ) AND ( adolescen* OR "young adult" OR child* OR "teen*" ) AND ( "low and middle income" OR "developing countr*" OR lmic* ) ) ) AND PUBYEAR > 2019 AND PUBYEAR < 2024

= 4

[LINK](https://www-scopus-com.proxy.lib.strath.ac.uk/results/results.uri?sort=plf-f&src=s&sid=80d8b82979f6da88122d7dc9fb404acf&sot=a&sdt=a&sl=400&s=TITLE-ABS-KEY%28%28%22digital+mental+health+intervention*%22+OR+%28%22digital+health+intervention%22+AND+%22mental+health%22%29+AND+%28mhealth+OR+ehealth+OR+smart*+OR+%22mobile+app*%22+OR+%22web+app*%22+OR+%22health+information+system*%22+OR+%22computer-assisted%22+OR+app*%29+AND+%28adolescen*+OR+%22young+adult%22+OR+child*+OR+%22teen*%22%29+AND+%28%22low+and+middle+income%22+OR+%22developing+countr*%22+OR+LMIC*%29%29%29+AND+PUBYEAR+%26gt%3B+2019+AND+PUBYEAR+%26lt%3B+2024&origin=searchadvanced&editSaveSearch=&txGid=b5f7323f18f34990c4a5df8a228c1921&sessionSearchId=80d8b82979f6da88122d7dc9fb404acf&limit=10)

5. ((“digital mental health intervention*” OR (“digital health intervention” AND “mental health”) OR “mental disorder*” OR “psychosocial intervention” OR “depressive disorder*” OR depression OR anxiet* OR “mood disorder*” OR “substance-related disorder*” OR “self-concept” OR “feeding and eating disorder*” OR “risk-taking” OR suicide) AND (smart* OR “web app*” OR “mobile app*” OR “health information system*” OR “computer-assisted” OR “cell phone” OR “Internet” OR “virtual” OR “artificial” OR “social media” OR “remote consultation” OR “video gam*”) AND (“adolescen*” OR “child*” OR “young adult” OR “teen*” OR “young people”) AND (“low and middle income” OR “developing countr*” OR "LMIC*") AND (“co-design" OR "participatory”) AND (“evaluation”))

TITLE-ABS-KEY ( ( ( "digital mental health intervention*" OR ( "digital health intervention" AND "mental health" ) OR "mental disorder*" OR "psychosocial intervention" OR "depressive disorder*" OR depression OR anxiet* OR "mood disorder*" OR "substance-related disorder*" OR "self-concept" OR "feeding and eating disorder*" OR "risk-taking" OR suicide ) AND ( smart* OR "web app*" OR "mobile app*" OR "health information system*" OR "computer-assisted" OR "cell phone" OR "Internet" OR "virtual" OR "artificial" OR "social media" OR "remote consultation" OR "video gam*" ) AND ( "adolescen*" OR "child*" OR "young adult" OR "teen*" OR "young people" ) AND ( "low and middle income" OR "developing countr*" OR "LMIC*" ) AND ( "co-design" OR "participatory" ) AND ( "evaluation" ) ) ) AND PUBYEAR > 2019 AND PUBYEAR < 2024

= 0

[LINK](https://www-scopus-com.proxy.lib.strath.ac.uk/results/results.uri?sort=plf-f&src=s&sid=74e9a002a2dc74add50cef3401616aa0&sot=a&sdt=a&sl=807&s=TITLE-ABS-KEY%28%28%28%22digital+mental+health+intervention*%22+OR+%28%22digital+health+intervention%22+AND+%22mental+health%22%29+OR+%22mental+disorder*%22+OR+%22psychosocial+intervention%22+OR+%22depressive+disorder*%22+OR+depression+OR+anxiet*+OR+%22mood+disorder*%22+OR+%22substance-related+disorder*%22+OR+%22self-concept%22+OR+%22feeding+and+eating+disorder*%22+OR+%22risk-taking%22+OR+suicide%29+AND+%28smart*+OR+%22web+app*%22+OR+%22mobile+app*%22+OR+%22health+information+system*%22+OR+%22computer-assisted%22+OR+%22cell+phone%22+OR+%22Internet%22+OR+%22virtual%22+OR+%22artificial%22+OR+%22social+media%22+OR+%22remote+consultation%22+OR+%22video+gam*%22%29+AND+%28%22adolescen*%22+OR+%22child*%22+OR+%22young+adult%22+OR+%22teen*%22+OR+%22young+people%22%29+AND+%28%22low+and+middle+income%22+OR+%22developing+countr*%22+OR+%22LMIC*%22%29+AND+%28%22co-design%22+OR+%22participatory%22%29+AND+%28%22evaluation%22%29%29%29+AND+PUBYEAR+%26gt%3B+2019+AND+PUBYEAR+%26lt%3B+2024&origin=searchadvanced&editSaveSearch=&txGid=f137d2babc76a69c8129278e29cdf286&sessionSearchId=74e9a002a2dc74add50cef3401616aa0&limit=10)

6. ((("digital mental health intervention*" OR ("digital health intervention" AND "mental health") OR "mental disorder*" OR "psychosocial intervention" OR "depressive disorder*" OR depression OR anxiet* OR "mood disorder*" OR "substance" OR "self-concept" OR "feeding and eating disorder*" OR "risk-taking" OR suicide) AND (smart* OR "web app*" OR "mobile app*" OR "health information system*" OR "computer-assisted" OR "cell phone" OR "Internet" OR "virtual" OR "artificial" OR "social media" OR "remote consultation" OR "video gam*")) AND (adolescents OR "adolescent") AND ("low and middle income" OR "developing countr*" OR "LMIC*") AND (co-design OR participatory) AND "evaluation")

TITLE-ABS-KEY ( ( ( ( "digital mental health intervention*" OR ( "digital health intervention" AND "mental health" ) OR "mental disorder*" OR "psychosocial intervention" OR "depressive disorder*" OR depression OR anxiet* OR "mood disorder*" OR "substance" OR "self-concept" OR "feeding and eating disorder*" OR "risk-taking" OR suicide ) AND ( smart* OR "web app*" OR "mobile app*" OR "health information system*" OR "computer-assisted" OR "cell phone" OR "Internet" OR "virtual" OR "artificial" OR "social media" OR "remote consultation" OR "video gam*" ) ) AND ( adolescents OR "adolescent" ) AND ( "low and middle income" OR "developing countr*" OR "LMIC*" ) AND ( co-design OR participatory ) AND "evaluation" ) ) AND PUBYEAR > 2019 AND PUBYEAR < 2024

= 0

[LINK](https://www-scopus-com.proxy.lib.strath.ac.uk/results/results.uri?sort=plf-f&src=s&sid=8bc056db38d640749e945a2997cf71cd&sot=a&sdt=a&sl=742&s=TITLE-ABS-KEY%28%28%28%28%22digital+mental+health+intervention*%22+OR+%28%22digital+health+intervention%22+AND+%22mental+health%22%29+OR+%22mental+disorder*%22+OR+%22psychosocial+intervention%22+OR+%22depressive+disorder*%22+OR+depression+OR+anxiet*+OR+%22mood+disorder*%22+OR+%22substance%22+OR+%22self-concept%22+OR+%22feeding+and+eating+disorder*%22+OR+%22risk-taking%22+OR+suicide%29+AND+%28smart*+OR+%22web+app*%22+OR+%22mobile+app*%22+OR+%22health+information+system*%22+OR+%22computer-assisted%22+OR+%22cell+phone%22+OR+%22Internet%22+OR+%22virtual%22+OR+%22artificial%22+OR+%22social+media%22+OR+%22remote+consultation%22+OR+%22video+gam*%22%29%29+AND+%28adolescents+OR+%22adolescent%22%29+AND+%28%22low+and+middle+income%22+OR+%22developing+countr*%22+OR+%22LMIC*%22%29+AND+%28co-design+OR+participatory%29+AND+%22evaluation%22%29%29+AND+PUBYEAR+%26gt%3B+2019+AND+PUBYEAR+%26lt%3B+2024&origin=searchadvanced&editSaveSearch=&txGid=aee8b35925b8d549fa71d320b398cf3f&sessionSearchId=8bc056db38d640749e945a2997cf71cd&limit=10)

1. **Web of Science**

(“digital health intervention*” **AND** “mental health” **AND** “adolescent*” **AND** “low and middle income”)

**3 results from Web of Science Core Collection for:**

Analyze Results

Citation Report

 Create Alert

**(“digital health intervention*” AND “mental health” AND “adolescent*” AND “low and middle income”)** (Topic)

Search

control_point Add Keywords

*| Timespan: 2019-01-01 to 2024-03-21 (Publication Date)*

[LINK](https://www-webofscience-com.proxy.lib.strath.ac.uk/wos/woscc/summary/83e885e9-fe6e-4f96-b683-4ea86ff128f4-d74db745/relevance/1)

1. ("digital mental health intervention" AND adolescent AND low and middle income OR LMIC AND (co-design OR participatory) AND evaluation)

15 results from Web of Science Core Collection for:

Analyze Results

Citation Report

Create Alert

("digital mental health intervention" AND adolescent AND low and middle income OR LMIC AND (co-design OR participatory) AND evaluation) (Topic)

Search

control_point Add Keywords

Quick add keywords:

keyboard_arrow_left

add LMIC

keyboard_arrow_right

| Timespan: 2019-01-01 to 2024-03-21 (Publication Date)

[LINK](https://www-webofscience-com.proxy.lib.strath.ac.uk/wos/woscc/summary/71121107-a4f4-4fb7-9fa4-fc1a5f35e836-d75399db/relevance/1)

1. ("digital mental health intervention*" OR (“digital health intervention” **AND** “mental health”) **AND** (“adolescen*” OR "young adult" OR “teen*”) **AND** ("low and middle income” OR "developing countr*" OR “LMIC*”))

**320 results from Web of Science Core Collection for:**

Analyze Results

Citation Report

 Create Alert

**("digital mental health intervention*" OR (“digital health intervention” AND “mental health”) AND (“adolescen*” OR "young adult" OR “teen*”) AND ("low and middle income” OR "developing countr*" OR “LMIC*”))** (Topic)

Search

control_point Add Keywords

**Quick add keywords:**

keyboard_arrow_left

add DIGITAL MENTAL HEALTH INTERVENTIONSadd DIGITAL MENTAL HEALTH INTERVENTIONadd DIGITAL MENTAL HEALTHadd DIGITAL MENTAL HEALTH INTERVENTIONS DMHISadd DIGITAL INTERVENTIONSadd HUMAN SUPPORTadd DIGITAL INTERVENTIONadd DMHIadd DIGITAL PSYCHIATRYadd E-MENTAL HEALTH

keyboard_arrow_right

*| Timespan: 2019-01-01 to 2024-03-24 (Publication Date)*

[LINK](https://www-webofscience-com.proxy.lib.strath.ac.uk/wos/woscc/summary/422cfb35-8f79-447d-a93b-c37cb858c451-d824f52c/relevance/1)

1. ("digital mental health intervention*" OR ("digital health intervention" AND "mental health") AND (mhealth OR ehealth OR smart* OR "mobile app*" OR "web app*" OR "health information system*" OR "computer-assisted" OR app*) AND (adolescen* OR "young adult" OR child* OR "teen*") AND ("low and middle income" OR "developing countr*" OR LMIC*))

321 results from Web of Science Core Collection for:

Analyze Results

Citation Report

 Create Alert

**("digital mental health intervention*" OR ("digital health intervention" AND "mental health") AND (mhealth OR ehealth OR smart* OR "mobile app*" OR "web app*" OR "health information system*" OR "computer-assisted" OR app*) AND (adolescen* OR "young adult" OR child* OR "teen*") AND ("low and middle income" OR "developing countr*" OR LMIC*))** (Topic)

Search

control_point Add Keywords

Quick add keywords:

keyboard_arrow_left

add DIGITAL MENTAL HEALTH INTERVENTIONSadd DIGITAL MENTAL HEALTH INTERVENTIONadd DIGITAL MENTAL HEALTHadd DIGITAL MENTAL HEALTH INTERVENTIONS DMHISadd DIGITAL INTERVENTIONSadd HUMAN SUPPORTadd DIGITAL INTERVENTIONadd DMHIadd DIGITAL PSYCHIATRYadd E-MENTAL HEALTH

keyboard_arrow_right

*| Timespan: 2019-01-01 to 2024-03-24 (Publication Date)*

[LINK](https://www-webofscience-com.proxy.lib.strath.ac.uk/wos/woscc/summary/f170abcb-3d09-42f4-b4e8-f17994eb1317-d82542e4/relevance/1)

1. ((“digital mental health intervention*” OR (“digital health intervention” AND “mental health”) OR “mental disorder*” OR “psychosocial intervention” OR “depressive disorder*” OR depression OR anxiet* OR “mood disorder*” OR “substance-related disorder*” OR “self-concept” OR “feeding and eating disorder*” OR “risk-taking” OR suicide) AND (smart* OR “web app*” OR “mobile app*” OR “health information system*” OR “computer-assisted” OR “cell phone” OR “Internet” OR “virtual” OR “artificial” OR “social media” OR “remote consultation” OR “video gam*”) AND (“adolescen*” OR “child*” OR “young adult” OR “teen*” OR “young people”) AND (“low and middle income” OR “developing countr*” OR "LMIC*") AND (“co-design" OR "participatory”) AND (“evaluation”))

Your search found no results

Check the spelling and/or broaden your search parameters

1. ((("digital mental health intervention*" OR ("digital health intervention" AND "mental health") OR "mental disorder*" OR "psychosocial intervention" OR "depressive disorder*" OR depression OR anxiet* OR "mood disorder*" OR "substance" OR "self-concept" OR "feeding and eating disorder*" OR "risk-taking" OR suicide) AND (smart* OR "web app*" OR "mobile app*" OR "health information system*" OR "computer-assisted" OR "cell phone" OR "Internet" OR "virtual" OR "artificial" OR "social media" OR "remote consultation" OR "video gam*")) AND (adolescents OR "adolescent") AND ("low and middle income" OR "developing countr*" OR "LMIC*") AND (co-design OR participatory) AND "evaluation")

Your search found no results

Check the spelling and/or broaden your search parameters

### Low- and middle-income country individual search

(List of LMICs obtained from the World Bank (2022): https://data.worldbank.org/income-level/Low-and-middle-income and searched within the listed databases, starting with Google Scholar).

1. AFGHANISTAN

((“digital mental health intervention*” OR (“digital health intervention” AND “mental health”) OR “mental disorder*” OR “psychosocial intervention” OR “depressive disorder*” OR depression OR anxiet* OR “mood disorder*” OR “substance-related disorder*” OR “self-concept” OR “feeding and eating disorder*” OR “risk-taking” OR suicide) AND (smart* OR “web app*” OR “mobile app*” OR “health information system*” OR “therapy computer assisted” OR “cell phone” OR “Internet” OR “social media” OR “Internet” OR “remote consultation” OR “video game*”) AND (“adolescen*” OR “child*” OR “young adult” OR “teen*”) AND (“low and middle income” OR “developing countr*”) AND (“co-design”) AND (“implementation”) AND “Afghanistan”)

or

((“digital mental health intervention*”) OR (“digital health intervention” AND “mental health”) AND (“adolescen*” OR “child*” OR “young adult” OR “teen*”) AND (“Afghanistan”))

2. ALBANIA

((“digital mental health intervention*”) OR (“digital health intervention” AND “mental health”) AND (“adolescen*” OR “child*” OR “young adult” OR “teen*”) AND (“Albania”))

3. ALGERIA

((“digital mental health intervention*”) OR (“digital health intervention” AND “mental health”) AND (“adolescen*” OR “child*” OR “young adult” OR “teen*”) AND (“Algeria”))

4. AMERICAN SAMOA

((“digital mental health intervention*”) OR (“digital health intervention” AND “mental health”) AND (“adolescen*” OR “child*” OR “young adult” OR “teen*”) AND (“American Samoa”))

5. ANGOLA

((“digital mental health intervention*”) OR (“digital health intervention” AND “mental health”) AND (“adolescen*” OR “child*” OR “young adult” OR “teen*”) AND (“Angola”))

or

((“digital mental health intervention*”) OR (“digital health intervention” AND “mental health”) OR (“mental disorder*” OR “psychosocial intervention” OR “depressive disorder*” OR depression OR anxiet* OR “mood disorder*” OR “substance-related disorder*” OR “self-concept” OR “feeding and eating disorder*” OR “risk-taking” OR suicide) AND (smart* OR “web app*” OR “mobile app*” OR “health information system*” OR “therapy computer assisted” OR “cell phone” OR “Internet” OR “social media” OR “Internet” OR “remote consultation” OR “video game*”) AND (“adolescen*” OR “child*” OR “young adult” OR “teen*”) AND (“low and middle income” OR “developing countr*”) AND (“co-design”) AND (“implementation”) AND (“Angola”))

6. ARGENTINA

((“digital mental health intervention*”) OR (“DMHI”) OR (“digital health intervention” AND “mental health”) AND (“adolescen*” OR “child*” OR “young adult” OR “teen*”) AND (“Argentina”))

7. ARMENIA

((“digital mental health intervention*”) OR (“DMHI”) OR (“digital health intervention” AND “mental health”) AND (“adolescent*” OR “child*” OR “young adult” OR “teen*”) AND (“Armenia”))

8. AZERBAIJAN

((“digital mental health intervention*”) OR (“DMHI”) OR (“digital health intervention” AND “mental health”) AND (“adolescent*” OR “child*” OR “young adult” OR “teen*”) AND (“Azerbaijan”))

9. BANGLADESH

((“digital mental health intervention*”) OR (“DMHI”) OR (“digital health intervention” AND “mental health”) AND (“adolescent*” OR “child*” OR “young adult” OR “teen*”) AND (“Bangladesh”))

10. BELARUS

((“digital mental health intervention*”) OR (“DMHI”) OR (“digital health intervention” AND “mental health”) AND (“adolescent*” OR “child*” OR “young adult” OR “teen*”) AND (“Belarus”))

11. BELIZE

((“digital mental health intervention*”) OR (“DMHI”) OR (“digital health intervention” AND “mental health”) AND (“adolescent*” OR “child*” OR “young adult” OR “teen*”) AND (“Belize”))

12. BENIN

((“digital mental health intervention*”) OR (“DMHI”) OR (“digital health intervention” AND “mental health”) AND (“adolescent*” OR “child*” OR “young adult” OR “teen*”) AND (“Benin”))

13. BHUTAN

((“digital mental health intervention*”) OR (“DMHI”) OR (“digital health intervention” AND “mental health”) AND (“adolescent*” OR “child*” OR “young adult” OR “teen*”) AND (“Bhutan”))

14. BOLIVIA

((“digital mental health intervention*”) OR (“DMHI”0 OR (“digital health intervention” AND “mental health”) AND (“adolescent*” OR “child*” OR “young adult” OR “teen*”) AND (“Bolivia”))

15. BOSNIA AND HERZEGOVINA

((“digital mental health intervention*”) OR (“DMHI”) OR (“digital health intervention” AND “mental health”) AND (“adolescent*” OR “child*” OR “young adult” OR “teen*”) AND (“Bosnia and Herzegovina”))

16. BOTSWANA

((“digital mental health intervention*”) OR (“DMHI”) OR (“digital health intervention” AND “mental health”) AND (“adolescent*” OR “child*” OR “young adult” OR “teen*”) AND (“Botswana”))

17. BRAZIL

((“digital mental health intervention*”) OR (“DMHI”) OR (“digital health intervention” AND “mental health”) AND (“adolescent*” OR “child*” OR “young adult” OR “teen*”) AND (“Brazil”))

18. BULGARIA

((“digital mental health intervention*”) OR (“DMHI”) OR (“digital health intervention” AND “mental health”) AND (“adolescent*” OR “child*” OR “young adult” OR “teen*”) AND (“Bulgaria”))

19. BURKINA FASO

((“digital mental health intervention*”) OR (“DMHI”) OR (“digital health intervention” AND “mental health”) AND (“adolescent*” OR “child*” OR “young adult” OR “teen*”) AND (“Burkina Faso”))

20. BURUNDI

((“digital mental health intervention*”) OR (“DMHI”) OR (“digital health intervention” AND “mental health”) AND (“adolescent*” OR “child*” OR “young adult” OR “teen*”) AND (“Burundi”))

21. CABO VERDE

((“digital mental health intervention*”) OR (“DMHI”) OR (“digital health intervention” AND “mental health”) AND (“adolescent*” OR “child*” OR “young adult” OR “teen*”) AND (“Cabo Verde”))

22. CAMBODIA

((“digital mental health intervention*”) OR (“DMHI”) OR (“digital health intervention” AND “mental health”) AND (“adolescent*” OR “child*” OR “young adult” OR “teen*”) AND (“Cambodia”))

23. CAMEROON

((“digital mental health intervention*”) OR (“DMHI”) OR (“digital health intervention” AND “mental health”) AND (“adolescent*” OR “child*” OR “young adult” OR “teen*”) AND (“Cameroon”))

24. CENTRAL AFRICAN REPUBLIC

((“digital mental health intervention*”) OR (“DMHI”) OR (“digital health intervention” AND “mental health”) AND (“adolescent*” OR “child*” OR “young adult” OR “teen*”) AND (“Central African Republic”))

25. CHAD

((“digital mental health intervention*”) OR (“DMHI”) OR (“digital health intervention” AND “mental health”) AND (“adolescent*” OR “child*” OR “young adult” OR “teen*”) AND (“Chad”))

26. CHINA

((“digital mental health intervention*”) OR (“DMHI”) OR (“digital health intervention” AND “mental health”) AND (“adolescent*” OR “child*” OR “young adult” OR “teen*”) AND (“China”))

27. COLOMBIA

((“digital mental health intervention*”) OR (“DMHI”) OR (“digital health intervention” AND “mental health”) AND (“adolescent*” OR “child*” OR “young adult” OR “teen*”) AND (“Colombia”))

28. COMOROS

((“digital mental health intervention*”) OR (“DMHI”) OR (“digital health intervention” AND “mental health”) AND (“adolescent*” OR “child*” OR “young adult” OR “teen*”) AND (“Comoros”))

29. CONGO, DEM. REP.

((“digital mental health intervention*”) OR (“DMHI”) OR (“digital health intervention” AND “mental health”) AND (“adolescent*” OR “child*” OR “young adult” OR “teen*”) AND (“Congo” AND “democratic”))

30. CONGO, REP.

((“digital mental health intervention*”) OR (“DMHI”) OR (“digital health intervention” AND “mental health”) AND (“adolescent*” OR “child*” OR “young adult” OR “teen*”) AND (“Congo” AND “republic”))

31. COSTA RICA

((“digital mental health intervention*”) OR (“DMHI”) OR (“digital health intervention” AND “mental health”) AND (“adolescent*” OR “child*” OR “young adult” OR “teen*”) AND (“Costa Rica”))

32. COTE D'IVOIRE

((“digital mental health intervention*”) OR (“DMHI”) OR (“digital health intervention” AND “mental health”) AND (“adolescent*” OR “child*” OR “young adult” OR “teen*”) AND (“Cote d’ivoire”))

33. CUBA

((“digital mental health intervention*”) OR (“DMHI”) OR (“digital health intervention” AND “mental health”) AND (“adolescent*” OR “child*” OR “young adult” OR “teen*”) AND (“Cuba”))

34. DJIBOUTI

((“digital mental health intervention*”) OR (“DMHI”) OR (“digital health intervention” AND “mental health”) AND (“adolescent*” OR “child*” OR “young adult” OR “teen*”) AND (“Djibouti”))

35. DOMINICA

((“digital mental health intervention*”) OR (“DMHI”) OR (“digital health intervention” AND “mental health”) AND (“adolescent*” OR “child*” OR “young adult” OR “teen*”) AND (“Dominica”))

36. DOMINICAN REPUBLIC

((“digital mental health intervention*”) OR (“DMHI”) OR (“digital health intervention” AND “mental health”) AND (“adolescent*” OR “child*” OR “young adult” OR “teen*”) AND (“Dominican Republic”))

37. ECUADOR

((“digital mental health intervention*”) OR (“DMHI”) OR (“digital health intervention” AND “mental health”) AND (“adolescent*” OR “child*” OR “young adult” OR “teen*”) AND (“Ecuador”))

38. EGYPT, ARAB REP.

((“digital mental health intervention*”) OR (“DMHI”) OR (“digital health intervention” AND “mental health”) AND (“adolescent*” OR “child*” OR “young adult” OR “teen*”) AND (“Egypt”))

39. EL SALVADOR

((“digital mental health intervention*”) OR (“DMHI”) OR (“digital health intervention” AND “mental health”) AND (“adolescent*” OR “child*” OR “young adult” OR “teen*”) AND (“El Salvador”))

40. EQUATORIAL GUINEA

((“digital mental health intervention*”) OR (“DMHI”) OR (“digital health intervention” AND “mental health”) AND (“adolescent*” OR “child*” OR “young adult” OR “teen*”) AND (“Equatorial Guinea”))

41. ERITREA

((“digital mental health intervention*”) OR (“DMHI”) OR (“digital health intervention” AND “mental health”) AND (“adolescent*” OR “child*” OR “young adult” OR “teen*”) AND (“Eritrea”))

42. ESWATINI

((“digital mental health intervention*”) OR (“DMHI”) OR (“digital health intervention” AND “mental health”) AND (“adolescent*” OR “child*” OR “young adult” OR “teen*”) AND (“Eswatini”))

43. ETHIOPIA

((“digital mental health intervention*”) OR (“DMHI”) OR (“digital health intervention” AND “mental health”) AND (“adolescent*” OR “child*” OR “young adult” OR “teen*”) AND (“Ethiopia”))

44. FIJI

((“digital mental health intervention*”) OR (“DMHI”) OR (“digital health intervention” AND “mental health”) AND (“adolescent*” OR “child*” OR “young adult” OR “teen*”) AND (“Fiji”))

45. GABON

((“digital mental health intervention*”) OR (“DMHI”) OR (“digital health intervention” AND “mental health”) AND (“adolescent*” OR “child*” OR “young adult” OR “teen*”) AND (“Gabon”))

46. GAMBIA, THE

((“digital mental health intervention*”) OR (“DMHI”) OR (“digital health intervention” AND “mental health”) AND (“adolescent*” OR “child*” OR “young adult” OR “teen*”) AND (“Gambia”))

47. GEORGIA

((“digital mental health intervention*”) OR (“DMHI”) OR (“digital health intervention” AND “mental health”) AND (“adolescent*” OR “child*” OR “young adult” OR “teen*”) AND (“Georgia”))

48. GHANA

((“digital mental health intervention*”) OR (“DMHI”) OR (“digital health intervention” AND “mental health”) AND (“adolescent*” OR “child*” OR “young adult” OR “teen*”) AND (“Ghana”))

49. GRENADA

((“digital mental health intervention*”) OR (“DMHI”) OR (“digital health intervention” AND “mental health”) AND (“adolescent*” OR “child*” OR “young adult” OR “teen*”) AND (“Grenada”))

50. GUATEMALA

((“digital mental health intervention*”) OR (“DMHI”) OR (“digital health intervention” AND “mental health”) AND (“adolescent*” OR “child*” OR “young adult” OR “teen*”) AND (“Guatemala”))

51. GUINEA

((“digital mental health intervention*”) OR (“DMHI”) OR (“digital health intervention” AND “mental health”) AND (“adolescent*” OR “child*” OR “young adult” OR “teen*”) AND (“Guinea”))

52. GUINEA-BISSAU

((“digital mental health intervention*) OR (“DMHI”) OR (“digital health intervention” AND “mental health”) AND (“adolescent*” OR “child*” OR “young adult” OR “teen*”) AND (“Guinea-Bissau”))

53. GUYANA

((“digital mental health intervention*”) OR (“DMHI”) OR (“digital health intervention” AND “mental health”) AND (“adolescent*” OR “child*” OR “young adult” OR “teen*”) AND (“Guyana”))

54. HAITI

((“digital mental health intervention*”) OR (“DMHI”) OR (“digital health intervention” AND “mental health”) AND (“adolescent*” OR “child*” OR “young adult” OR “teen*”) AND (“Haiti”))

55. HONDURAS

((“digital mental health intervention*”) OR (“DMHI”) OR (“digital health intervention” AND “mental health”) AND (“adolescent*” OR “child*” OR “young adult” OR “teen*”) AND (“Honduras”))

56. INDIA

((“digital mental health intervention*”) OR (“DMHI”) OR (“digital health intervention” AND “mental health”) AND (“adolescent*” OR “child*” OR “young adult” OR “teen*”) AND (“India”))

57. INDONESIA

((“digital mental health intervention*”) OR (“DMHI”) OR (“digital health intervention” AND “mental health”) AND (“adolescent*” OR “child*” OR “young adult” OR “teen*”) AND (“Indonesia”))

58. IRAN, ISLAMIC REP.

((“digital mental health intervention*”) OR (“DMHI”) OR (“digital health intervention” AND “mental health”) AND (“adolescent*” OR “child*” OR “young adult” OR “teen*”) AND (“Iran”))

59. IRAQ

((“digital mental health intervention*”) OR (“DMHI”) OR (“digital health intervention” AND “mental health”) AND (“adolescent*” OR “child*” OR “young adult” OR “teen*”) AND (“Iraq”))

60. JAMAICA

((“digital mental health intervention*”) OR (“DMHI”) OR (“digital health intervention” AND “mental health”) AND (“adolescent*” OR “child*” OR “young adult” OR “teen*”) AND (“Jamaica”))

61. JORDAN

((“digital mental health intervention*”) OR (“DMHI”) OR (“digital health intervention” AND “mental health”) AND (“adolescent*” OR “child*” OR “young adult” OR “teen*”) AND (“Jordan”))

62. KAZAKHSTAN

((“digital mental health intervention*”) OR (“DMHI”) OR (“digital health intervention” AND “mental health”) AND (“adolescent*” OR “child*” OR “young adult” OR “teen*”) AND (“Kazakhstan”))

63. KENYA

((“digital mental health intervention*”) OR (“DMHI”) OR (“digital health intervention” AND “mental health”) AND (“adolescent*” OR “child*” OR “young adult” OR “teen*”) AND (“Kenya”))

64. KIRIBATI

((“digital mental health intervention*”) OR (“DMHI”) OR (“digital health intervention” AND “mental health”) AND (“adolescent*” OR “child*” OR “young adult” OR “teen*”) AND (“Kiribati”))

65. KOREA, DEM. PEOPLE'S REP.

((“digital mental health intervention*”) OR (“DMHI”) OR (“digital health intervention” AND “mental health”) AND (“adolescent*” OR “child*” OR “young adult” OR “teen*”) AND (“Korea”))

66. KOSOVO

((“digital mental health intervention*”) OR (“DMHI”) OR (“digital health intervention” AND “mental health”) AND (“adolescent*” OR “child*” OR “young adult” OR “teen*”) AND (“Kosovo”))

67. KYRGYZ REPUBLIC

((“digital mental health intervention*”) OR (“DMHI”) OR (“digital health intervention” AND “mental health”) AND (“adolescent*” OR “child*” OR “young adult” OR “teen*”) AND (“Kyrgyz”))

68. LAO PDR

((“digital mental health intervention*”) OR (“DMHI”) OR (“digital health intervention” AND “mental health”) AND (“adolescent*” OR “child*” OR “young adult” OR “teen*”) AND (“Lao”))

69. LEBANON

((“digital mental health intervention*”) OR (“DMHI”) OR (“digital health intervention” AND “mental health”) AND (“adolescent*” OR “child*” OR “young adult” OR “teen*”) AND (“Lebanon”))

70. LESOTHO

((“digital mental health intervention*”) OR (“DMHI”) OR (“digital health intervention” AND “mental health”) AND (“adolescent*” OR “child*” OR “young adult” OR “teen*”) AND (“Lesotho”))

71. LIBERIA

((“digital mental health intervention*”) OR (“DMHI”) OR (“digital health intervention” AND “mental health”) AND (“adolescent*” OR “child*” OR “young adult” OR “teen*”) AND (“Liberia”))

72. LIBYA

((“digital mental health intervention*”) OR (“DMHI”) OR (“digital health intervention” AND “mental health”) AND (“adolescent*” OR “child*” OR “young adult” OR “teen*”) AND (“Libya”))

73. MADAGASCAR

((“digital mental health intervention*”) OR (“DMHI”) OR (“digital health intervention” AND “mental health”) AND (“adolescent*” OR “child*” OR “young adult” OR “teen*”) AND (“Madagascar”))

74. MALAWI

((“digital mental health intervention*”) OR (“DMHI”) OR (“digital health intervention” AND “mental health”) AND (“adolescent*” OR “child*” OR “young adult” OR “teen*”) AND (“Malawi”))

75. MALAYSIA

((“digital mental health intervention*”) OR (“DMHI”) OR (“digital health intervention” AND “mental health”) AND (“adolescent*” OR “child*” OR “young adult” OR “teen*”) AND (“Malaysia”))

76. MALDIVES

((“digital mental health intervention*”) OR (“DMHI”) OR (“digital health intervention” AND “mental health”) AND (“adolescent*” OR “child*” OR “young adult” OR “teen*”) AND (“Maldives”))

77. MALI

((“digital mental health intervention*”) OR (“DMHI”) OR (“digital health intervention” AND “mental health”) AND (“adolescent*” OR “child*” OR “young adult” OR “teen*”) AND (“Mali”))

78. MARSHALL ISLANDS

((“digital mental health intervention*”) OR (“DMHI”) OR (“digital health intervention” AND “mental health”) AND (“adolescent*” OR “child*” OR “young adult” OR “teen*”) AND (“Marshall Islands”))

79. MAURITANIA

((“digital mental health intervention*”) OR (“DMHI”) OR (“digital health intervention” AND “mental health”) AND (“adolescent*” OR “child*” OR “young adult” OR “teen*”) AND (“Mauritania”))

80. MAURITIUS

((“digital mental health intervention*”) OR (“DMHI”) OR (“digital health intervention” AND “mental health”) AND (“adolescent*” OR “child*” OR “young adult” OR “teen*”) AND (“Mauritius”))

81. MEXICO

((“digital mental health intervention*”) OR (“DMHI”) OR (“digital health intervention” AND “mental health”) AND (“adolescent*” OR “child*” OR “young adult” OR “teen*”) AND (“Mexico”))

82. MICRONESIA, FED. STS.

((“digital mental health intervention*”) OR (“DMHI”) OR (“digital health intervention” AND “mental health”) AND (“adolescent*” OR “child*” OR “young adult” OR “teen*”) AND (“Micronesia”))

83. MOLDOVA

((“digital mental health intervention*”) OR (“DMHI”) OR (“digital health intervention” AND “mental health”) AND (“adolescent*” OR “child*” OR “young adult” OR “teen*”) AND (“Moldova”))

84. MONGOLIA

((“digital mental health intervention*”) OR (“DMHI”) OR (“digital health intervention” AND “mental health”) AND (“adolescent*” OR “child*” OR “young adult” OR “teen*”) AND (“Mongolia”))

85. MONTENEGRO

((“digital mental health intervention*”) OR (“DMHI”) OR (“digital health intervention” AND “mental health”) AND (“adolescent*” OR “child*” OR “young adult” OR “teen*”) AND (“Montenegro”))86.

86. MOROCCO

((“digital mental health intervention*”) OR (“DMHI”) OR (“digital health intervention” AND “mental health”) AND (“adolescent*” OR “child*” OR “young adult” OR “teen*”) AND (“Morocco”))

87. MOZAMBIQUE

((“digital mental health intervention*”) OR (“DMHI”) OR (“digital health intervention” AND “mental health”) AND (“adolescent*” OR “child*” OR “young adult” OR “teen*”) AND (“Mozambique”))

88. MYANMAR

((“digital mental health intervention*”) OR (“DMHI”) OR (“digital health intervention” AND “mental health”) AND (“adolescent*” OR “child*” OR “young adult” OR “teen*”) AND (“Myanmar”))

89. NAMIBIA

((“digital mental health intervention*”) OR (“DMHI”) OR (“digital health intervention” AND “mental health”) AND (“adolescent*” OR “child*” OR “young adult” OR “teen*”) AND (“Namibia”))

90. NEPAL

((“digital mental health intervention*”) OR (“DMHI”) OR (“digital health intervention” AND “mental health”) AND (“adolescent*” OR “child*” OR “young adult” OR “teen*”) AND (“Nepal”))

91. NICARAGUA

((“digital mental health intervention*”) OR (“DMHI”) OR (“digital health intervention” AND “mental health”) AND (“adolescent*” OR “child*” OR “young adult” OR “teen*”) AND (“Nicaragua”))

92. NIGER

((“digital mental health intervention*”) OR (“DMHI”) OR (“digital health intervention” AND “mental health”) AND (“adolescent*” OR “child*” OR “young adult” OR “teen*”) AND (“Niger”))

93. NIGERIA

((“digital mental health intervention*”) OR (“DMHI”) OR (“digital health intervention” AND “mental health”) AND (“adolescent*” OR “child*” OR “young adult” OR “teen*”) AND (“Nigeria”))

94. NORTH MACEDONIA

((“digital mental health intervention*”) OR (“DMHI”) OR (“digital health intervention” AND “mental health”) AND (“adolescent*” OR “child*” OR “young adult” OR “teen*”) AND (“Macedonia”))

95. PAKISTAN

((“digital mental health intervention*”) OR (“DMHI”) OR (“digital health intervention” AND “mental health”) AND (“adolescent*” OR “child*” OR “young adult” OR “teen*”) AND (“Pakistan”))

96. PALAU

((“digital mental health intervention*”) OR (“DMHI”) OR (“digital health intervention” AND “mental health”) AND (“adolescent*” OR “child*” OR “young adult” OR “teen*”) AND (“Palau”))

97. PAPUA NEW GUINEA

((“digital mental health intervention*”) OR (“DMHI”) OR (“digital health intervention” AND “mental health”) AND (“adolescent*” OR “child*” OR “young adult” OR “teen*”) AND (“Papua New Guinea”))

98. PARAGUAY

((“digital mental health intervention*”) OR (“DMHI”) OR (“digital health intervention” AND “mental health”) AND (“adolescent*” OR “child*” OR “young adult” OR “teen*”) AND (“Paraguay”))

99. PERU

((“digital mental health intervention*”) OR (“DMHI”) OR (“digital health intervention” AND “mental health”) AND (“adolescent*” OR “child*” OR “young adult” OR “teen*”) AND (“Peru”))

100. PHILIPPINES

((“digital mental health intervention*”) OR (“DMHI”) OR (“digital health intervention” AND “mental health”) AND (“adolescent*” OR “child*” OR “young adult” OR “teen*”) AND (“Philippines”))

101. RUSSIAN FEDERATION

((“digital mental health intervention*”) OR (“DMHI”) OR (“digital health intervention” AND “mental health”) AND (“adolescent*” OR “child*” OR “young adult” OR “teen*”) AND (“Russian Federation”))

102. RWANDA

((“digital mental health intervention*”) OR (“DMHI”) OR (“digital health intervention” AND “mental health”) AND (“adolescent*” OR “child*” OR “young adult” OR “teen*”) AND (“Rwanda”))

103. SAMOA

((“digital mental health intervention*”) OR (“DMHI”) OR (“digital health intervention” AND “mental health”) AND (“adolescent*” OR “child*” OR “young adult” OR “teen*”) AND (“Samoa”))

104. SAO TOME AND PRINCIPE

((“digital mental health intervention*”) OR (“DMHI”) OR (“digital health intervention” AND “mental health”) AND (“adolescent*” OR “child*” OR “young adult” OR “teen*”) AND (“Sao Tome and Principle”))

105. SENEGAL

((“digital mental health intervention*”) OR (“DMHI”) OR (“digital health intervention” AND “mental health”) AND (“adolescent*” OR “child*” OR “young adult” OR “teen*”) AND (“Senegal”))

106. SERBIA

((“digital mental health intervention*”) OR (“DMHI”) OR (“digital health intervention” AND “mental health”) AND (“adolescent*” OR “child*” OR “young adult” OR “teen*”) AND (“Serbia”))

107. SIERRA LEONE

((“digital mental health intervention*”) OR (“DMHI”) OR (“digital health intervention” AND “mental health”) AND (“adolescent*” OR “child*” OR “young adult” OR “teen*”) AND (“Sierra Leone”))

108. SOLOMON ISLANDS

((“digital mental health intervention*”) OR (“DMHI”) OR (“digital health intervention” AND “mental health”) AND (“adolescent*” OR “child*” OR “young adult” OR “teen*”) AND (“Solomon Islands”))

109. SOMALIA

((“digital mental health intervention*”) OR (“DMHI”) OR (“digital health intervention” AND “mental health”) AND (“adolescent*” OR “child*” OR “young adult” OR “teen*”) AND (“Somalia”))

110. SOUTH AFRICA

((“digital mental health intervention*”) OR (“DMHI”) OR (“digital health intervention” AND “mental health”) AND (“adolescent*” OR “child*” OR “young adult” OR “teen*”) AND (“South Africa”))

111. SOUTH SUDAN

((“digital mental health intervention*”) OR (“DMHI”) OR (“digital health intervention” AND “mental health”) AND (“adolescent*” OR “child*” OR “young adult” OR “teen*”) AND (“South Sudan”))

112. SRI LANKA

((“digital mental health intervention*”) OR (“DMHI”) OR (“digital health intervention” AND “mental health”) AND (“adolescent*” OR “child*” OR “young adult” OR “teen*”) AND (“Sri Lanka”))

113. ST. LUCIA

((“digital mental health intervention*”) OR (“DMHI”) OR (“digital health intervention” AND “mental health”) AND (“adolescent*” OR “child*” OR “young adult” OR “teen*”) AND (“St. Lucia”))

114. ST. VINCENT AND THE GRENADINES

((“digital mental health intervention*”) OR (“DMHI”) OR (“digital health intervention” AND “mental health”) AND (“adolescent*” OR “child*” OR “young adult” OR “teen*”) AND (“St. Vincent and the Grenadines”))

115. SUDAN

((“digital mental health intervention*”) OR (“DMHI”) OR (“digital health intervention” AND “mental health”) AND (“adolescent*” OR “child*” OR “young adult” OR “teen*”) AND (“Sudan”))

116. SURINAME

((“digital mental health intervention*”) OR (“DMHI”) OR (“digital health intervention” AND “mental health”) AND (“adolescent*” OR “child*” OR “young adult” OR “teen*”) AND (“Suriname”))

117. SYRIAN ARAB REPUBLIC

((“digital mental health intervention*”) OR (“DMHI”) OR (“digital health intervention” AND “mental health”) AND (“adolescent*” OR “child*” OR “young adult” OR “teen*”) AND (“Syria”))

118. TAJIKISTAN

((“digital mental health intervention*”) OR (“DMHI”) OR (“digital health intervention” AND “mental health”) AND (“adolescent*” OR “child*” OR “young adult” OR “teen*”) AND (“Tajikistan”))

119. TANZANIA

((“digital mental health intervention*”) OR (“DMHI”) OR (“digital health intervention” AND “mental health”) AND (“adolescent*” OR “child*” OR “young adult” OR “teen*”) AND (“Tanzania”))

120. THAILAND

((“digital mental health intervention*”) OR (“DMHI”) OR (“digital health intervention” AND “mental health”) AND (“adolescent*” OR “child*” OR “young adult” OR “teen*”) AND (“Thailand”))

121. TIMOR-LESTE

((“digital mental health intervention*”) OR (“DMHI”) OR (“digital health intervention” AND “mental health”) AND (“adolescent*” OR “child*” OR “young adult” OR “teen*”) AND (“Timor”))

122. TOGO

((“digital mental health intervention*”) OR (“DMHI”) OR (“digital health intervention” AND “mental health”) AND (“adolescent*” OR “child*” OR “young adult” OR “teen*”) AND (“Togo”))

123. TONGA

((“digital mental health intervention*”) OR (“DMHI”) OR (“digital health intervention” AND “mental health”) AND (“adolescent*” OR “child*” OR “young adult” OR “teen*”) AND (“Tonga”))

124. TUNISIA

((“digital mental health intervention*”) OR (“DMHI”) OR (“digital health intervention” AND “mental health”) AND (“adolescent*” OR “child*” OR “young adult” OR “teen*”) AND (“Tunisia”))

125. TURKIYE

((“digital mental health intervention*”) OR (“DMHI”) OR (“digital health intervention” AND “mental health”) AND (“adolescent*” OR “child*” OR “young adult” OR “teen*”) AND (“Turkey”))

126. TURKMENISTAN

((“digital mental health intervention*”) OR (“DMHI”) OR (“digital health intervention” AND “mental health”) AND (“adolescent*” OR “child*” OR “young adult” OR “teen*”) AND (“Turkmenistan”))

127. TUVALU

((“digital mental health intervention*”) OR (“DMHI”) OR (“digital health intervention” AND “mental health”) AND (“adolescent*” OR “child*” OR “young adult” OR “teen*”) AND (“Tuvalu”))

128. UGANDA

((“digital mental health intervention*”) OR (“DMHI”) OR (“digital health intervention” AND “mental health”) AND (“adolescent*” OR “child*” OR “young adult” OR “teen*”) AND (“Uganda”))

129. UKRAINE

((“digital mental health intervention*”) OR (“DMHI”) OR (“digital health intervention” AND “mental health”) AND (“adolescent*” OR “child*” OR “young adult” OR “teen*”) AND (“Ukraine”))

130. UZBEKISTAN

((“digital mental health intervention*”) OR (“DMHI”) OR (“digital health intervention” AND “mental health”) AND (“adolescent*” OR “child*” OR “young adult” OR “teen*”) AND (“Uzbekistan”))

131. VANUATU

((“digital mental health intervention*”) OR (“DMHI”) OR (“digital health intervention” AND “mental health”) AND (“adolescent*” OR “child*” OR “young adult” OR “teen*”) AND (“Vanuatu”))

132. VIETNAM

((“digital mental health intervention*”) OR (“DMHI”) OR (“digital health intervention” AND “mental health”) AND (“adolescent*” OR “child*” OR “young adult” OR “teen*”) AND (“Vietnam”))

133. WEST BANK AND GAZA

((“digital mental health intervention*”) OR (“DMHI”) OR (“digital health intervention” AND “mental health”) AND (“adolescent*” OR “child*” OR “young adult” OR “teen*”) AND (“West Bank and Gaza”))

134. YEMEN, REP.

((“digital mental health intervention*”) OR (“DMHI”) OR (“digital health intervention” AND “mental health”) AND (“adolescent*” OR “child*” OR “young adult” OR “teen*”) AND (“Yemen”))

135. ZAMBIA

((“digital mental health intervention*”) OR (“DMHI”) OR (“digital health intervention” AND “mental health”) AND (“adolescent*” OR “child*” OR “young adult” OR “teen*”) AND (“Zambia”))

136. ZIMBABWE

((“digital mental health intervention*”) OR (“DMHI”) OR (“digital health intervention” AND “mental health”) AND (“adolescent*” OR “child*” OR “young adult” OR “teen*”) AND (“Zimbabwe”))
