# Supplementary material for: Digital Mental Health Interventions for Adolescents in Low- and Middle-Income Countries: Scoping Review
Source: J Med Internet Res. 2024 Oct 29;26:e51376. doi: 10.2196/51376 (PMC11558223; doi:10.2196/51376)
Supplement: Multimedia Appendix 4 [file jmir_v26i1e51376_app4.docx]

**Review Question:**

What is known about digital mental health interventions for adolescents in low- and middle-income countries, as reported in the literature?

**Sub-Review Questions:**

**SubRQ1**: How are adolescent digital mental health interventions designed and evaluated within low- and middle-income countries?

**SubRQ2**: What are the reported activities and approaches involving adolescents in designing digital mental health interventions, and what are the perceived outcomes of such involvement?

**SubRQ3**: Which frameworks, toolkits, models, or theories were consulted or applied to the digital mental health intervention? (including implementation activities related to digital health readiness and preparedness)

**SubRQ4**: What factors facilitate or hinder adolescent engagement in digital mental health interventions in low- and middle-income countries?

**Table S1.** Characteristics of the included digital mental health interventions.

|  | | **SubRQ1** | | | | **SubRQ2** | **SubRQ3** | **SubRQ4** |  |
| --- | --- | --- | --- | --- | --- | --- | --- | --- | --- |
| **Study #** | **Lead author, date of publication, country of lead author.** | **DMHI name, description, target mental health disorder, start year of intervention implementation, country of DMHI implementation** | **Participant age, sample size.** | **Study aim, type, intervention (including group/individual, lay support), control, evaluation** | **Outcome measures, duration, follow-up, and costs** | **Adolescent involvement in DMHI design** | **Consulted frameworks, theories, or models** | **Factors affecting adolescent engagement with the DMHI (facilitators or barriers)** | **Key findings** |
|  |  |  |  |  |  |  |  |  |  |
| 1 | Al-Khayat (2021), Norway | **Happy Helping Hand** – Digital game for psychosocial support (PSS), facilitated by teachers and psychosocial support via iPads, smartphones, or computers (**well-being and emotional problem-solving**)  **Settings:** Schools in Lebanon  **Start year of intervention:** 2020  **Country of DMHI:** Lebanon | Displaced Syrian adolescents.  12-17 years  n = 104 | **Aim**: to explore the Happy Helping Hand app as a digital psychosocial support program in education and emotional problem-solving skills and how important such interventions are for displaced Syrian adolescents who live in Lebanon.  **Type of study:** Mixed-method approach (focus group interviews and a survey with teachers/ Psychosocial support, staff, and displaced adolescents)  **Intervention:** Happy Helping Hand digital PSS app – 10 sessions. Group delivery - 10 adolescents plus teacher/PSS staff per group)  **Control:** N/A  **Evaluation:** N/A | Outcome measure(s):  **Primary outcome:** WHO 5 Well-Being Index (WHO-5) (subjective wellbeing) - taken before and directly after the end of the intervention  **Secondary outcome:** Feasibility and usefulness  **Duration**:  5 weeks (2 sessions/  week)  **Follow-up**: N/A  **Costs:** N/A | Existing DMHI (launched in Norway), to be adapted to the Arab cultural context | - Social constructivism  - Social-ecological model (SEM)  - Cognitive behavioral model  - Social learning theory (SLT) | **Facilitators**  **-** culturally adapting the DMHI to the Arab context  **barriers**  **-** COVID-19/lockdown as not all participants had access to the Internet or digital devices  - lack of cultural appropriateness meant less engagement with the DMHI  - time constraints on delivery of app by PSS staff/teachers | - the Happy Helping Hand positively affected Syrian displaced adolescents’ well-being  - The WHO 5 wellbeing index showed a total of 7.92% change in 97 adolescents‘ well-being after playing the Happy Helping Hand app.  - The game’s feasibility and usefulness was inconclusive.  - The app should be culturally adapted to the Arabian context, and parents should be involved in the intervention. |
| 2 | Anttila *et al.* (2019), Finland | **DepisNet-Thai** – Web-based program –  an expansion and implementation of good practices obtained from previous web programs for persons with mental health problems (**general mental health disorders**)  **Setting:** School (3 high schools)  **Start year of intervention:** 2013  **Country of DMHI**: Thailand | Thai adolescents  15-19 years  n = 167 | **Aim:** to study the impact, acceptance, and usability of a web program, and make a comparison between program users and non-users  **Type of study:** quasi-experimental feasibility study  **Intervention:**  **Intervention 1**: DepisNet-Thai (small groups), teachers were trained to run the program and how to act as a tutor.    A handbook for DepisNet-Thai was developed for tutors in the Thai language.  **Intervention 2:** Active control (individually)  **Control - Intervention 3:** Passive control - No access to the DepisNet-Thai  **Evaluation:** N/A | Outcome measure(s):  **Primary outcome:**  - Patient Health  Questionnaire (PHQ-9)  (depression)  - The Perceived Stress Scale (T-PSS-10) (stress)  - Client Satisfaction Questionnaire (CSQ-8)  - Technology Acceptance Model (usability feedback)  **Duration:**  7 weeks (5 weekly modules lasting 50 mins)  **Follow-up:** Baseline and a follow-up conducted after intervention  **Costs:** N/A | Content of DepisNet-Thai was developed based on discussions with adolescents in Thailand showing stress mainly to be a problem. | - Theories of coping during adolescence  - Self-determination theory  - Technology Acceptance Model | **Barriers**  - Challenges with user emails, meant several emails were sent and may have reduced acceptance of the program  - Timing: Semester examinations prevented engagement with the DMHI  - Reduced motivation to return the exercises  - Program length may have hindered participation | For depression, changes from baseline to follow-up did not differ significantly between the groups (p = 0.594).  For stress, the changes between baseline and follow-up did not differ significantly between the groups (p = 0.178).  For satisfaction, the changes between baseline and follow-up did not differ significantly between the groups (p = 0.101).  77% of all web program users were satisfied with DepisNet-Thai regardless of their group.  Overall, there is no proof that the DepisNet-Thai program is beneficial in terms of decreased depression, stress scores and increased satisfaction among users in comparison to those who did not log on. |
| 3 | Chory *et al*. (2021), USA | **Intervention via WhatsApp®** - Smartphone mobile intervention delivered via the WhatsApp® platform with a trained pediatric HIV adherence and disclosure counselor (**depression**)  **Setting**: Care clinic  **Start year of intervention**: N/A  **Country of DMHI:** Kenya | Kenyan adolescents living with HIV (ALWH)  10-19 years  n=30 | **Aims:** examine HIV-related knowledge, attitudes, beliefs, behavior, and experiences of Kenyan ALWH revealed in the contextual data from enrolment in a WhatsApp® group chat intervention  **Type of study:** Qualitative analysis (part of a broader mixed-method pilot study)  **Intervention:** Mobile Phone Intervention via WhatsApp®. A trained pediatric HIV adherence and disclosure counselor facilitates the WhatsApp® groups. Weekly counselor led modules (1-2hrs)  **Control:** N/A  **Evaluation:** N/A | **Outcome measure(s):** Outcome measures not reported in this paper but reported in the broader included study (Chory *et al*. 2022)  **Duration**: 6 -month intervention,  **Follow-up:** Baseline, 3 months, 6 months  **Costs:** N/A | N/A | N/A | **Facilitators**  - a safe space to discuss and understand HIV-related specifics  - able to discuss barriers to anti-retroviral (ART) adherence  **Barriers**  - stigma and discrimination | - ALWH engaged meaningfully with the HIV education and peer support intervention via WhatsApp®  - the platform was helpful in facilitating critical conversations and information seeking. Medication adherence and navigating challenges such as stigma |
| 4 | Chory *et al*. (2022), USA | **Intervention via WhatsApp®** - Smartphone mobile intervention delivered via the WhatsApp® platform with a trained pediatric HIV adherence and disclosure counselor (**depression**)  **Setting**: Care clinic  **Start year of intervention**: N/A  **Country of DMHI:** Kenya | Kenyan adolescents living with HIV (ALWH)  10-19 years  n=30 | **Aim:** to evaluate the acceptability and feasibility of the WhatsApp® platform to deliver individual counseling services and facilitate peer support for ALWH in western Kenya  **Type of study:** Prospective mixed-method pilot study  **Intervention:** Mobile Phone Intervention via WhatsApp®. A trained pediatric HIV adherence and disclosure counselor facilitates the WhatsApp® groups. Weekly counselor led modules (1-2hrs)  **Control:** N/A  **Evaluation:** N/A | **Outcome measure(s):**  **Primary outcomes:**  - Patient Health Questionnaire-9 (PHQ-9) (depression)  - Hopkins Symptom Checklist for Depression  - Strengths and Difficulties Questionnaire (SDQ) (symptom checklist for depression)  - Comprehensive ART Measure for Pediatrics (CAMP) Adherence Questionnaire (adherence to HIV treatment)  - Medical Event Monitoring System (electronic dose monitoring)  - Stigma in AIDS Family Inventory (SAFI) questionnaire (stigma)  **Duration**: 6-month intervention,  **Follow-up**:  Baseline, 3 months, 6 months.  **Costs:** N/A | N/A | N/A | **Facilitators**  - the provision of a smartphone with the WhatsApp® application preinstalled, a SIM card, and phone credit (~ 7 USD per month)  - Inclusion of the trained pediatric HIV adherence and disclosure counselor  -ALWH sense of community and peer support  - Access to a counselor in the group or privately from the group  **Barriers**  - boarding school attendance prevented one adolescent participant  - household and school responsibilities  - logistical issues e.g., power outages, insufficient phone credit, internet problems, and learning to use the mobile phone  - Lack of access to a smartphone /mobile phone (would be expensive at scale) | - the WhatsApp® intervention was an acceptable and feasible platform for mobile counseling and peer support for ALWH  - low rates of mental and behavioral disorders using the PHQ-9, SDQ, and Hopkins Symptoms Checklist. This could be attributed to the lack of validation of tools to evaluate mental health in settings among ALWH. |
| 5 | Duan *et al.* (2020), China | **SMS text messaging intervention** – SMS delivered to reduce deliberate self-harm (**self-harm**)  **Setting**: Hospital  **Start year of intervention**: N/A  **Country of DMHI:** China | Chinese adolescents with deliberate self-harm  12-17 years  n=23 | **Aim:** to investigate the views of Chinese adolescents with deliberate self-harm about SMS text messaging interventions to develop an acceptable and culturally competent intervention for adolescents with deliberate self-harm  **Type of study:** Qualitative study  **Intervention:** SMS text messaging intervention (individuals)  **Control:** N/A  **Evaluation:** N/A | **Outcome measure(s):** N/A  **Duration:** Anticipated 2-6 months  **Follow-up:** N/A  **Costs:** N/A | N/A | N/A | **Facilitators**  - access to research-based information.  - a contact number of someone to talk to when the adolescent wants to self-harm.  - parental involvement, such as receiving SMS messages.  - reasonable frequency and duration of the SMS texts.  - the timing of the SMS, e.g., during evenings when self-harm urges are increased.  - friendly content, tailored service.  **Barriers**  - parents keep adolescents’ mobile phones for most of the day.  - long duration of intervention could lead to disengagement. | - the SMS intervention has the potential to decrease deliberate self-harm instances by providing acceptable support for adolescents with deliberate self-harm who may be reluctant to seek face-to-face psychotherapies.  - the findings will contribute to the development of a culturally appropriate SMS text messaging intervention for adolescents with deliberate self-harm |
| 6 | Gómez-Restrepo *et al.* (2023), Colombia | **DIALOG+** is a digital app-supported and evidence-based intervention created to assist in the interaction between patients with a mental health condition and clinicians. (**mental well-being and resilience**)  **Setting**: Two schools in a post-conflict area of Colombia  **Start year of intervention**: N/A  **Country of DMHI:** Colombia | Adolescents in post-armed conflict Colombia  12-18 years  n = 70 | **Aim:** to adapt an existing patient-focused digital intervention called DIALOG+ from an adult clinical setting to an adolescent educational setting and to assess the feasibility, acceptability, and estimated effect of implementing this intervention as a tool for promoting quality of life, mental well-being, and resilience.  **Type of Study: Exploratory cluster randomized** controlled trial (exploratory mixed methods: Quantitative (cluster RCT), Qualitative (focus groups). It included 3 phases: adaptation phase (focus groups to adapt intervention), exploration phase (exploratory cluster randomized controlled trial - a total of 14 clusters), and *consolidation phase* (focus groups).  **Intervention: DIALOG+S -** app installed on multiple electronic devices (e.g., a tablet or cellphone). Guides the patient to evaluate their satisfaction with 8 quality-of-life areas (mental health, physical health, work, accommodation, leisure activities, friends, relationship with family and partner, and personal safety), and 3 treatment aspects (medication, practical help, and professional appointments). They are delivered by teachers.  **Control:** counseling as usual  **Evaluation:** RCT (impact evaluation) | Outcome measure(s):  **Primary outcome** **measures**:  -The Family adaptability, partnership, growth, affection, and resolve (APGAR)  -The Self-Reporting Questionnaire to assess mental health problems (SRQ)  - The 8-item Patient Health Questionnaire depression scale to assess depression symptoms (PHQ-8)  - The Generalized Anxiety Disorder 7-item scale to assess anxiety symptoms (GAD-7)  - The PTSD Checklist to measure symptoms  - The 25-item Connor Davidson Resilience Scale to measure resilience (CD-RISC-25)  -The Rosenberg scale to measure self-esteem    - The Manchester Short Assessment of Quality of Life to measure quality of life (MANSA)  **Duration:** 6-months  **Follow-up:** baseline and 6-months  **Costs:** N/A | Existing DMHI | N/A | **Facilitators**  **-** including domains related to academic performance and student life  - academic support from teachers and counselors  **Barrier/facilitator**  - COVID-19 exposed the overlooked social and personal needs of adolescents | - the quantitative data from the exploratory phase showed small improvements in the final assessment of the DIALOG+S group, suggesting that the intervention has the potential to improve aspects of adolescents’ mental health e.g., perceived quality of life, resilience, and anxiety symptoms (as seen in the MANSA, CD-RISC-25, and PHQ-8 scores, respectively)  - the intervention was deemed feasible and acceptable in a school setting |
| 7 | Gonsalves *et al.* (2019), India | **POD (identifying “Problems,” generating “Options,” and creating a “Do it” plan) Adventures –** Problem-solving game-based intervention, delivered by smartphone (**depression and anxiety**)  **Setting**: Schools in India  **Start year of intervention:** 2017  **Country of DMHI:** India | Adolescents from India  12-17 years  n=46 (focus groups)  n=22 (co-design workshops)  n=50 (user testing) | **Aim:** to design and develop POD Adventures, a blended problem-solving game-based intervention for adolescents with or at risk of anxiety, depression, and conduct difficulties in India.  **Type of study:** Qualitative study (included focus group discussions, co-design workshops, user testing with students and service providers)  **Intervention:** POD (identifying “Problems,” generating “Options,” and creating a “Do it” plan) Adventures  **Control:** N/A  **Evaluation:** N/A | **Outcome measure(s):** NA  **Duration:** N/A  **Follow-up:** N/A  **Costs:** N/A | Adolescents involved in the design of the intervention via:  (i) exploring a selection of popular games and apps such as Temple Run, Candy Crush, Tekken, Horizon Chase, and Kaun Banega Crorepati?  (ii) story building to create personas and problem scenarios  (iii) paper prototyping of game components and characters  and (iv) discussion about prototype ideas presented by the participants. | - The Self-Determination Theory (SDT)  - The framework for Persuasive Systems Design (PSD)  - Person-based approach to Digital Health-Related Behavior Change Interventions  - World Health Organization (WHO) guidelines on monitoring and evaluating digital health interventions (digital maturity and readiness) | **Facilitators**  - different learning styles for understanding abstract concepts  - support from a counselor for intervention success,  - gamified features  - Digital delivery allowed for personalization and better engagement  - offline access  -privacy and confidentiality  **Barriers**  - concerns from teachers and parents on the time spent on phones.  - Local infrastructural limitations and lack of online functionality | - the findings contributed to the design of the POD Adventures intervention.  - the digital blended self-help format was a culturally acceptable to school-going adolescents in this context.  - an RCT is planned to evaluate processes and impacts of POD Adventures when delivered for help-seeking students in low-resource school settings. |
| 8 | Gonsalves *et al.* (2021), India | **PRIDE (PRemIum for aDolEscents, 2016-2022) -** stepped-care intervention targeting common mental health problems in school-going adolescents, smartphone, and Internet-based delivery (**general mental health conditions**)  **Setting:** Schools  **Start year of intervention:** 2021 (COVID-19 interrupted the study)  **Country of DMHI:** India | Adolescents  13-19 years  Aiming for a sample size of at least n = 70 participants (35 per arm) | **Aim:** to assess the feasibility of research procedures and intervention delivery and to generate preliminary estimates of the effectiveness of the intervention to inform the sample size calculation of a full-scale trial  **Type of Study:** Parallel, 2-arm, individually randomized pilot-controlled trial (RCT) – protocol  **Intervention: POD Adventures—A 2-minute app overview video is followed by a 1:1 brief telephone “onboarding” session with a counsellor, after which participants can use the app** at their own time.  - On-demand telephone support from a counselor will be available  - Internet format – can be delivered by a non-specialist (“lay”) counselor in under-resourced school settings in individual and group delivery  **Control:** digital flyer consisting of information and contact details of local mental health service providers and 2 government provided helplines  **Evaluation**: RCT | **Outcome measure(s):**  **Feasibility outcome**:  - User satisfaction questionnaire at 6 weeks  **Clinical outcome:**  - Youth top problems (YTP) (psychosocial problem severity)    - Revised Child Anxiety and Depression Scale–Short Version (RCADS-25) (depression and anxiety)  **Duration:** 4 weeks (30 mins/ week)  **Follow-up:** Baseline and follow-up at 6 months  **Costs:** N/A | Co-design workshops with young people and iterative piloting suggested that the optimal delivery mode for POD Adventures involved small group sessions with up to 6 students working independently on smartphones under the supervision of a counselor | - Stress-coping theory | - Study underway  **Barrier/facilitator**  - COVID-19 restrictions offered specific insights into the viability of delivering and evaluating psychosocial interventions under conditions of social distancing and school closures  **Barrier**  - implementation barriers include a shortage of suitably trained, supervised, and motivated school counselors | - Study underway |
| 9 | Hall *et al*. (2022), Switzerland | **STARS (Sustainable Technology for Adolescents and youth to Reduce Stress) -** WHO transdiagnostic chatbot for distressed youth (**distress)**  **Setting**: N/A  **Start year of intervention**: N/A  **Country of DMHI:** Piloted in South Africa (An RCT in Jordan is forthcoming) | Adolescents  15-18 years  n= N/A | **Aim:** to develop and test an evidence-based digital psychological intervention for youth experiencing high levels of psychological distress  **Type of study:** Intervention design: human-centred design (HCD) methods to create the WHO STARS project.  - Delivered through existing chatbot systems using different technologies (e.g., apps, websites, messaging platforms) - 10 chat sessions (10 mins/session)  **Intervention: individually delivered STARS (Sustainable Technology for Adolescents and youth to Reduce Stress)**.  **Control:** N/A  **Evaluation:** N/A | **Outcome measure(s):** N/A  **Duration:** N/A  **Follow-up:** N/A  **Costs:** N/A | N/A | - Human centered design (HCD) methods  - Cognitive behavioral model | N/A | - Study underway (forthcoming RCT)  - Formative work potentially pending in South Africa, Pakistan, Jamaica, Nepal, and occupied Palestinian territories |
|  |  |  |  |  |  |  |  |  |  |
|  |  |  |  |  |  |  |  |  |  |
| 10 | Moeini *et al*. (2019), Iran | **DAD (Dorehye Amozeshie Dokhtaran) course** - Web-based education program (**depression**)  **Setting:** female schools  **Start year of Intervention:** 2018  **Country of DMHI:** Iran | Female adolescent students in high school  15-18 years  n=128 | **Aim:** to examine the effectiveness of a web-based intervention for depressive symptoms in female adolescents  **Type of study:** RCT  **Description of study:**  **Intervention: DAD (Dorehye Amozeshie Dokhtaran) -** Individual access with constant online assistance from a psychiatrist using a secure online messaging system  **Control:** Assumed treatment as usual  **Evaluation:** RCT | **Outcome measure(s):**  **Primary outcome:**  - The Center for Epidemiological Studies Depression Scale (CES-D) evaluates the occurrence of 20 depressive symptoms  - The Farsi version of the Perceived Social Support Scale-Revised (PSSS-R)  - The Farsi version of the Sherer's general self-efficacy questionnaire was used to measure social cognitive theory constructs.  **Duration:** 6 months (8 x 30min sessions)  **Follow-up:** Baseline and follow-up at 24 weeks  **Costs:** N/A | N/A | - Social Cognitive Theory (SCT) | **Facilitator**  - professional support from a psychiatrist  - having non digital support, e.g., books, video, and the website. | - The intervention program improved depression in 12 weeks based on Intent-to-treat analyses but seem to have decreased by 24 weeks in intervention group.  - The intervention had no effect on outcome expectations and self-efficacy.  - There were no statistically significant associations between theory construct changes and changes in depression (P>0.05) |
| 11 | Mukherjee *et al*. (2024), India | **Adolescents’ Resilience and Treatment nEeds for Mental Health in Indian Slums (ARTEMIS) Intervention –** combines an anti-stigma campaign with a digital health intervention to identify and manage mental health conditions, delivered by tablets.  **(depression, self-harm/stigma/ suicide risk or other significant emotional complaints)**  **Setting:** Urban Primary Health Centre (UPHC) in slum clusters  **Start year of Intervention:**  2020  **Country of DMHI:** India | Adolescents  15-19 years involved in the theory of change workshop.  n= 2752 adolescents screened | **Aim:** to report the results of the formative research phase that has informed the development of the intervention for the main ARTEMIS trial:  1. to obtain information on the local context (including key stakeholders), factors influencing the uptake of mental health services, and common stressors among adolescents living in the study area.  2. to assess the feasibility of rolling out a community-based anti-stigma campaign and the acceptability of anti-stigma information education communication (IEC) content among adolescents.  3. to assess the feasibility of rolling out the mHealth component of the intervention and acceptability of the electronic decision support system (EDSS) among Accredited Social Health activists (ASHAs) and urban primary health center (UPHC) doctors.  **Type of study:** mixed methods formative evaluation  **Intervention:** ARTEMIS intervention.  **Control**: N/A  **Evaluation**: formative evaluation | **Outcome measure(s):** N/A  **Duration**: N/A  **Follow-up:** N/A  **Costs:** N/A | Adolescents were involved (along with other stakeholders) in developing a theory of change (ToC) for the intervention and the co-design of anti-stigma IEC materials with the AEAG. | - UK Medical Research Council (MRC) guidance for developing and evaluating complex interventions | **Facilitators**  - using  peer groups comprising 10 to 15 adolescents to provide lay counseling and mental health support for adolescents in the community.  - audio-visual and game-based methods for the anti-stigma campaign  - A list of key takeaway messages for each activity will be prepared.  **Barriers**  - Questions related to suicide and pregnancy in the baseline survey tool and EDSS tool caused discomfort among adolescents  - Mental health-related stigma amongst parents  - lack of time due to long work hours  - financial constraints of the families  - reluctance of parents to engage due to stigma | - The formative evaluation of the intervention strategy led to enhanced understanding of the context, acceptability, and feasibility of the intervention.  - strategies to improve implementation included engaging with parents, organizing health camps on the sites, and forming peer groups.  - The mHealth component of the intervention was feasible and acceptable among community health workers and doctors. |
|  |  |  |  |  |  |  |  |  |  |
| 12 | Osborn *et al.*(2020), Kenya | **Shamiri-Digital -** Internet-based universal, computerized self-help single session intervention (SSI) (**depression, anxiety, and wellbeing**)  **Setting:** Schools in Kenya  **Start year of Intervention:** 2019  **Country of DMHI:** Kenya | Adolescents  13 – 18 years  n = 103 | **Aim:** to report on the outcomes of a RCT on Kenyan adolescents  **Type of study:** RCT  **Intervention:** **Shamiri-Digital -** delivered via computers in school - consists of three modules: growth mindset, gratitude, and value affirmation – lay counselor delivery  **Control:** Study Skills  **Evaluation:** RCT | **Outcome measure(s):**  **Primary outcome:**  - Patient Health Questionnaire– 8 (PHQ-8) (depression)  - Generalized Anxiety Disorder Screener–7 (GAD) (anxiety)  **Secondary outcome:**  - Engagement, Perseverance, Optimism, Connectedness, and Happiness Measure of Adolescent Well-being (EPOCH)  SWEMWBS - a shortened version of the Warwick–Edinburgh Mental Well-Being Scale  **Duration:** 4 weeks  **Follow-up:** Baseline and 2-week follow-up  **Costs:** N/A | Existing DMHI | N/A | **Facilitators**  - delivering the DMHI around periods of time with less distraction for the adolescents e.g., outside of high-stress periods may increase the adolescents’ receptiveness to the DMHI  - Private school setting meant the availability of computers and internet connection (not all schools in Kenya have this access) | - Compared to the control, Shamiri-Digital produced a more significant reduction in adolescent depressive symptoms in both the total sample (*p* = .028, *d* = 0.50) and a subsample of youths with moderate to severe depression symptoms (*p* = .010, *d* =0.83) from baseline to 2-week follow-up.  - The effects exceed the mean effects reported in meta-analyses of full-length, face-to-face psychotherapy for youth depression.  - There were no significant effects on anxiety symptoms, well-being, or happiness. |
|  |  |  |  |  |  |  |  |  |  |
| 13 | Pozuelo *et al*. (2023), USA | **Kuamsha app** - delivers behavioral activation (BA) to treat depression among adolescents, smartphone delivery (**depression**)  **Setting:** School  **Start year of Intervention:** 2019  **Country of DMHI:** South Africa and Uganda | Adolescents  15-19 years  n=160 | **Aim:** to describe the user-centered development of a smartphone app that  delivers behavioral activation (BA) to treat depression among adolescents in rural South Africa and Uganda and to summarise the findings from multicycle usability testing.  **Type of study:** multicycle usability testing  **Intervention: Kuamsha app** – 6 modules taking 15-20mins to complete, supported by a brief weekly phone call from a peer mentor to help users overcome barriers to engagement, individually delivered.  **Control:** N/A  **Evaluation:** feasibility studies in Uganda and South Africa | **Outcome measure(s):** N/A  **Duration:** 11-week intervention  **Follow-up:** N/A  **Costs**: N/A | - iterative co-design process with adolescents from rural South Africa and Uganda, who guided the development and provided feedback at each developmental phase | - Person-based approach to Digital Health-Related Behavior Change Interventions  - Behavioural activation | **Facilitators**  -use of stories, gamification, good aesthetics, content that targets common issues,  - creating of a safe space  weekly phone calls from peer mentors (lay workers)  **Barriers**  - Phone data running out often and having to wait to top up again.    - Storage – the app takes up too much space.  - If the confidentiality is not guaranteed and others can access what is on the app | - The Kuamsha app uniquely delivered BA for adolescent depression via an interactive narrative game format tailored to the South African and Ugandan contexts.  - Further studies are currently underway to examine the intervention’s feasibility, acceptability, and efficacy in reducing depressive symptoms |
| 14 | Raknes (2020), Norway | **Happy Helping Hand** – Digital game for psychosocial support(PSS), a cognitive behavioral-based tool where children learn how to problem solve, not for learning only, but for their healthy development (**well-being and emotional problem-solving**)  **Setting:** Tents in the camp where adolescents were living  **Start year of Intervention:** 2020  **Country of DMHI:** Lebanon | Syrian adolescent refugees in Lebanon  13-17 years  n=10 | **Aim:** to evaluate the feasibility and usefulness of the HH, and to examine the potential impact of the game in improving adolescents´ psychosocial wellbeing  **Type of study:** mixed method study  **Intervention: Happy Helping Hand** - A manual was used to guide the 10- sessions PSS program implemented. The game has ten scenarios, and in the PSS manual, each of these is the basis for one PSS session, guided by a PSS group leader – psychologist and facilitated by teachers and psychosocial support via Smartphones or tablets, group delivery.  **Control: N/A**  **Evaluation:** feasibility and usefulness | **Outcome measure(s):**  A five items questionnaire (scale from 0 – 10) which was specifically designed for this study was used to assess the feasibility and usefulness of the HH game.  **Duration:** 10 weeks  **Follow-up: N/A**  **Costs:** N/A | Existing DMHI – created with user involvement from Syrian refugee adolescents, Norwegian adolescents, and Lebanese, Palestinian, Syrian, and Norwegian health professionals, and teachers | - Cognitive-behavioural model | **Barriers**  - cost of intervention  - stigma associated with help-seeking  - mental health is a taboo in some Arab contexts  - lack of culturally adapted evidence-based programs  - Digital divide and poverty are barriers to the implementation of the HH app. | - Syrian refugees living in poverty found the happy HH game feasible and useful.  - Adolescent well-being significantly increased after the use of the game. |
| 15 | Raknes & Chorna (2024), Norway | **Happy Helping Hand** – Digital game for psychosocial support (**well-being and emotional problem-solving**)  **Setting:** School  **Date of intervention:** 2023  **Country of DMHI:** Ukraine | Ukrainian adolescents  13-17 years  n=10 | **Aims**: to evaluate the feasibility and potential impact of the HH program among adolescents in Ukraine  **Type of study**: Mixed methods  **Intervention:** A 10-session blended learning group program, a cognitive behavioral intervention, was implemented by a psychologist. A professional translation company translated the HH game into Ukrainian.  **Control group:** N/A  **Evaluation**: Adolescents complete a paper and pen questionnaire pre- and post-intervention. The data are interpreted by the psychologist who implemented the program. | **Outcome measure(s):**  feasibility questionnaire made for the study.  - The Revised Children's Anxiety and Depression Scale (RCADS) is a 25-item questionnaire  - The World Health Organization´s five items include the Wellbeing Index (WHO-5) (wellbeing assessed pre-intervention only).    **Duration:** 2 months, weekly 90 min sessions  **Follow-up:** N/A  **Costs:** N/A | Existing DMHI (Norway) to be adapted for Ukraine adolescents | - Cognitive behavioral model | **Facilitator**  - Inclusion of the psychologist  - the likable game  **Barriers**  - language errors were reported but not as a primary barrier. | - the HH program was feasible and showed promise in reducing anxiety and depression symptoms, enhancing communication, facilitating support-seeking behaviors, and improving emotional well-being.  - Decrease in average anxiety and depression symptoms (RCADS using Cohen´s d) from a mean score of 20.4 (SD = 14.39) before the intervention to 15.0 (SD = 8.29) afterward. |
| 16 | Srivastava *et al.* (2020), USA | **smartteen -** Computer-assisted CBT intervention - a computer application designed to augment in-person CBT for the treatment of depression in adolescents. (**depression**)  **Setting:** hospitals  **Start year of Intervention:** N/A  **Country of DMHI:** India | Adolescents with unipolar depression seeking treatment at a tertiary care hospital.  13-19 years  n=21 | **Aim:** to present results of the pilot evaluation of smartteen (a cCBT) for its feasibility, acceptability, and effectiveness in reducing depressive symptoms  **Type of Study:** Single-blinded clinical randomized control trial  **Intervention: smartteen** - Interactive multi-media presentation featuring text, flowcharts, 2D animations, graphics, quizzes, and puzzles. The twelve sessions are in a linear progression, with each session building on the knowledge gained in previous sessions and on the tasks carried out at home and delivered with therapist support.  **Control:** treatment as usual  **Evaluation:** RCT | **Outcome measure(s):**  **Primary outcome:**  - Beck’s Depression Inventory-II (BDI-II)  - Children’s Depression Rating Scale Revised (CRDS-R)  - Children’s Global Assessment Scale (CGAS)  - Clinician’s Global Impression Scale-Symptom Severity Subscale (CGI-S)  **Duration:** 12 weeks  **Follow-up:** baseline, mid-treatment (6 weeks) and post-treatment (12 weeks)  **Costs:** N/A | - intervention not described as co-design, but involved group discussions with adolescents having depression | - Cognitive-behavioural model  - Adolescent-centric user design | **Facilitators**  - high customizability, personalization, e.g., therapists could personalize the app for the participant’s needs.  **Barriers**  - minor technical issues with the smartteen intervention (e.g., occasional browser crashing, occasional occurrences of a diary entry not saving)  - issues such as distraction while using smartteen due to other ongoing sessions in the same clinic room. | - At 12 weeks, smartteen was significantly more effective than TAU in reducing depression symptoms and improving functioning on CGAS.  - Treatment compliance was better in smartteen group.  - smartteen was shown to reduce the time spent by therapists to deliver 12 sessions of CBT treatment for depression. |
| 17 | Superable (2022), Philippines and LMICs | **Self-help game-based mobile application intervention –** teaches service users skills that help them address mental health (psychosocial intervention) (**mild/moderate depression and anxiety**)  **Setting:** School  **Start year of Intervention:**  2021  **Country of DMHI:** Philippines and potentially other LMICs | Adolescents with mental health difficulties  12-19 years  n=N/A | **Aim:** to find ideas that can become highly impactful and cost-effective charities in improving the well-being of the people living in the Philippines and other LMICs, with a focus on child and adolescent mental health  **Type of study:**  Qualitative study  **Intervention: self-help game-based mobile application—school-based, evidence-based app—includes minimal guidance from lay workers, peer-to-peer support, group delivery with independent game play, and** four 30 – 40-minute sessions that are spanned for 2 – 4 weeks. The intervention is currently ideated to be delivered in a group session, with 5 – 6 members each, to maximize counselor time.  **Control:** N/A  **Evaluation:** N/A | **Outcome measure(s):** N/A  **Duration:** four 30 – 40-minute sessions that are spanned for 2 – 4 weeks  **Follow-up:** N/A  **Costs:** Cost-effective evaluation conducted. | - notes the importance of involving adolescents and key stakeholders such as parents, teachers, and mental health providers to determine adolescent’s challenges, coping strategies, and help-seeking behaviors to ensure the appropriateness of the treatment component and content for the service users | Cognitive behavioral model | **Facilitators**  - mobile phone ownership is more common than computers in underserved communities  - privacy or discretion it provides  - tailored for Philippine settings – cultural settings  - gamified app  - Remote supervision can be conducted using digital tools through mobile calls or online conferencing  **Barriers**  – complexity of app implementation, e.g., lay counselor training, app development, mental health service referrals.  - Associated stigma against mental health.  - The digital ownership and infrastructure in the Philippines limit the users who may benefit from the intervention  - poor internet connectivity and limited access to devices in rural areas | - the app is evidence-based and could potentially reduce emotional and behavioral problems and improve the well-being of adolescents.  - compared to other interventions proposed, the program might take longer to implement due to the multitude and complexity of its components, namely, lay counselor training and supervision, app development, stakeholder partnerships with schools, and mental health services for referrals  - the sustainability of the program remains uncertain and will depend on the feasibility of integrating this into the government health system |
| 18 | Wasil *et al*. (2020), USA | **three computerized single-session interventions (SSIs) -** Self-guided computerized mental health intervention (**depression and anxiety**)  **Setting:** Schools  **Start year of Intervention:** 2019  **Country of DMHI:** India | Adolescents  10-19 years  n= 15 (second focus group - students) | **Aim:** to examine the acceptability, feasibility, and appropriateness of three interventions among school leadership and students.  **Type of study:** Pilot study (intervention design, focus groups, hybrid Type 1 effectiveness-implementation study to evaluate the interventions  **Intervention:** **three computerized single-session interventions (SSIs) -**  Self-guided computerized mental health intervention - delivered via school computer labs - based on behavioral activation, gratitude, and growth mindset exercises.  **Control:** N/A  **Evaluation:** related multi-arm RCT underway | **Outcome measure(s):** N/A  **Duration:** Single session intervention  **Follow-up:** Follow up noted in trial description at baseline, 1 and 3-months  **Costs:** N/A | Adolescent students did not co-design the interventions but offered useful suggestions via focus group discussions | - Cognitive behavioral model - growth mindsets (drawn from social, educational, and clinical psychology), gratitude (drawn from positive psychology), and behavioral activation (drawn from reviews of youth psychotherapy protocols). | **Facilitators**  - inclusion of short testimonials of how the intervention helped other students  - short breaks in the middle of the intervention  - students desired more reading materials about research related to each concept.  - students had several suggestions to make the vignettes in the “saying is believing” exercise more relatable  - better aesthetics  - using Commonwealth spelling (rather than American English) | - Computerised SSIs may offer innovative, scalable ways to disseminate evidence-based interventions. |
| 19 | Wasil *et al.* (2021), USA | **Shamiri-Digital –** Online single-session intervention (**focuses on growth mindset, gratitude, and value affirmations**)  And  **Digital CBT** – Online single-session intervention (focuses on behavioral activation, cognitive restructuring, and problem-solving)  (**depression and anxiety**)  **Setting:** Schools  **Start year of Intervention:** 2021  **Country of DMHI:** Kenya | Kenyan adolescents  13-19 years  n = 926 | **Aim:** to evaluate the effectiveness of two digital single-session interventions (Shamiri-Digital and DigitalCBT (cognitive-behavioral therapy)) among Kenyan adolescents.  **Type of study:** Double-blind RCT with three arms: Shamiri-Digital, Digital-CBT- protocol  **Intervention 1:** Shamiri-Digital—delivered electronically in school computer labs—consists of three modules: growth mindset, gratitude, and value affirmation groups.  **Intervention** 2—Digital CBT, delivered electronically in school computer labs, focuses on behavioral activation, cognitive restructuring, and problem-solving groups.  **Control:** Online single-session study skills – focuses on effective study habits  **Evaluation:** RCT | **Outcome measure(s):**  **Primary outcome (intervention):**  - Patient Health Questionnaire-8 (PHQ-8) – (depression)  - Generalized Anxiety Disorders (GAD-7) – (anxiety)  - Short Warwick-Edinburgh Mental Well-being Scale – (subjective wellbeing)  **secondary outcome (intervention):**  - Acceptability of Intervention Measure (AIM)  Intervention Appropriateness Measure (IAM)  **primary outcome** **(control):**  - Perceived Control Scale for Children (PCSC) – (extent individuals believe they can affect objective conditions in their lives)  **secondary outcome (control):**  - Secondary Control Scale for Children (SCSC) – (extent individuals believe they can manage the personal and psychological impact of objective events)  **Duration:** Single session intervention  **Follow-up:** Baseline and 2, 4, and 12-week follow-up  **Costs:** N/A | Existing DMHI  – adapted for Kenyan adolescents | - Cognitive behavioral model | N/A | Study underway |
| 20 | Yatirajula *et al.*(2022), India | **Adolescents’ Resilience and Treatment nEeds for Mental Health in Indian Slums (ARTEMIS)** adapted version of the Systematic Medical Appraisal, Referral and Treatment (SMART) Mental Health Programme and is a multifaceted program that includes an anti-stigma campaign (**Stigma, depression, self-harm, suicide**)  **Setting:** Urban Primary Health Centre (UPHC) in slum clusters  **Start year of Intervention:** 2022  **Country of DMHI:** India | **High-risk and non-high-risk adolescents for depression, suicide, self-harm**  10-19 years  n = 3960 (aiming to recruit 1980 per arm) | **Aim:** to develop and evaluate a complex intervention incorporating an anti-stigma campaign and a mobile technology-based electronic decision support systems (EDSS) to facilitate the delivery of mental health care for depression, other significant emotional or medically unexplained complaints, and increased risk of self-harm/ suicide among adolescents living in urban slums.  **Type of study:** Cluster randomized control trial (cRCT) – protocol  **Intervention:** ARTEMIS/ SMART project - ARTEMIS intervention – The software will be developed on an Android platform, optimized for 7-inch tablets - anti-stigma campaign and electronic decision support system  **Control:** usual healthcare  **Evaluation:** RCT | **Outcome measure(s):**  - Patient Health Questionnaire (PHQ9)  - Knowledge, Attitude, and Behaviour (KAB) scale  - Barriers to Access to Care Evaluation- Treatment Stigma (BACE-TS)  - Connor-Davidson Resilience Scale (CD-RISC - 10)  **Duration:** 12 months  **Follow-up:** 3, 6, 12 months  **Costs:** economic evaluation to be conducted. | N/A | - WHO’s Mental Health Gap Action Programme- Intervention Guide (mhGAP-IG)  - Michie’s Behaviour Change Theory | Study underway | Study underway |

1. Al-Khayat, A. M. (2021). *Digitalized Psychosocial Support in Education: exploring the impact of the Happy Helping Hand app for displaced Syrian adolescents in Lebanon* (Master's thesis, OsloMet-Storbyuniversitetet).
2. Anttila, M., Sittichai, R., Katajisto, J., & Välimäki, M.. (2019). Impact of a Web Program to Support the Mental Wellbeing of High School Students: A Quasi Experimental Feasibility Study. *International Journal of Environmental Research and Public Health*, *16*(14), 2473. <https://doi.org/10.3390/ijerph16142473>
3. Chory, A., Nyandiko, W., Martin, R., Aluoch, J., Scanlon, M., Ashimosi, C., Njoroge, T., Mcateer, C., Apondi, E., & Vreeman, R.. (2021). HIV-Related Knowledge, Attitudes, Behaviors and Experiences of Kenyan Adolescents Living with HIV Revealed in WhatsApp Group Chats. *Journal of the International Association of Providers of AIDS Care (JIAPAC)*, *20*, 232595822199957. <https://doi.org/10.1177/2325958221999579>
4. Chory, A., Callen, G., Nyandiko, W., Njoroge, T., Ashimosi, C., Aluoch, J., Scanlon, M., Mcateer, C., Apondi, E., & Vreeman, R.. (2022). A Pilot Study of a Mobile Intervention to Support Mental Health and Adherence Among Adolescents Living with HIV in Western Kenya. *AIDS and Behavior*, *26*(1), 232–242. <https://doi.org/10.1007/s10461-021-03376-9>
5. Duan, S., Wang, H., Wilson, A., Qiu, J., Chen, G., He, Y., Wang, Y., Ou, J., & Chen, R.. (2020). Developing a Text Messaging Intervention to Reduce Deliberate Self-Harm in Chinese Adolescents: Qualitative Study. *JMIR Mhealth and Uhealth*, *8*(6), e16963. <https://doi.org/10.2196/16963>
6. Gómez-Restrepo, C., Sarmiento-Suárez, M. J., Alba-Saavedra, M., Calvo-Valderrama, M. G., Rincón-Rodríguez, C. J., Bird, V. J., Priebe, S., & Van Loggerenberg, F.. (2023). Development and Implementation of DIALOG+S in the School Setting as a Tool for Promoting Adolescent Mental Well-Being and Resilience in a Post–Armed Conflict Area in Colombia: Exploratory Cluster Randomized Controlled Trial. *JMIR Formative Research*, *7*, e46757. <https://doi.org/10.2196/46757>
7. Gonsalves, P. P., Hodgson, E. S., Kumar, A., Aurora, T., Chandak, Y., Sharma, R., Michelson, D., & Patel, V.. (2019). Design and Development of the “POD Adventures” Smartphone Game: A Blended Problem-Solving Intervention for Adolescent Mental Health in India. *Frontiers in Public Health*, *7*. <https://doi.org/10.3389/fpubh.2019.00238>
8. Gonsalves, P. P., Sharma, R., Hodgson, E., Bhat, B., Jambhale, A., Weiss, H. A., Fairburn, C. G., Cavanagh, K., Cuijpers, P., Michelson, D., & Patel, V.. (2021). A Guided Internet-Based Problem-Solving Intervention Delivered Through Smartphones for Secondary School Pupils During the COVID-19 Pandemic in India: Protocol for a Pilot Randomized Controlled Trial. *JMIR Research Protocols*, *10*(10), e30339. <https://doi.org/10.2196/30339>
9. Hall, J., Jordan, S., Van Ommeren, M., Au, T., Sway, R. A., Crawford, J., Ghalayani, H., Hamdani, S. U., Luitel, N. P., Malik, A., Servili, C., Sorsdahl, K., Watts, S., & Carswell, K.. (2022). Sustainable Technology for Adolescents and youth to Reduce Stress (STARS): a WHO transdiagnostic chatbot for distressed youth. *World Psychiatry*, *21*(1), 156–157. https://doi.org/10.1002/wps.20947
10. Moeini, B., Bashirian, S., Soltanian, A. R., Ghaleiha, A., & Taheri, M. (2019). Examining the Effectiveness of a Web-Based Intervention for Depressive Symptoms in Female Adolescents: Applying Social Cognitive Theory. *Journal of research in health sciences*, *19*(3), e00454.
11. Mukherjee, A., Yatirajula, S. K., Kallakuri, S., Paslawar, S., Lempp, H., Raman, U., Essue, B. M., Sagar, R., Singh, R., Peiris, D., Norton, R., Thornicroft, G., & Maulik, P. K.. (2024). Using formative research to inform a mental health intervention for adolescents living in Indian slums: the ARTEMIS study. *Child and Adolescent Psychiatry and Mental Health*, *18*(1). <https://doi.org/10.1186/s13034-024-00704-4>
12. Osborn, T. L., Rodriguez, M., Wasil, A. R., Venturo-Conerly, K. E., Gan, J., Alemu, R. G., ... & Weisz, J. R. (2020). Single-session digital intervention for adolescent depression, anxiety, and well-being: Outcomes of a randomized controlled trial with Kenyan adolescents. *Journal of consulting and clinical psychology*, *88*(7), 657.
13. Pozuelo, J. R., Moffett, B. D., Davis, M., Stein, A., Cohen, H., Craske, M. G., Maritze, M., Makhubela, P., Nabulumba, C., Sikoti, D., Kahn, K., Sodi, T., Van Heerden, A., & O’Mahen, H. A.. (2023). User-Centered Design of a Gamified Mental Health App for Adolescents in Sub-Saharan Africa: Multicycle Usability Testing Study. *JMIR Formative Research*, *7*, e51423. <https://doi.org/10.2196/51423>
14. Raknes, S. (2020). The Happy Helping Hand used by Syrian displaced adolescents in Lebanon: a pilot study of feasibility, usefulness, and impact. *White paper*.
15. Raknes, S., & Chorna, T.. (2024). *The Helping Hand in Ukraine: Feasibility and Potential Impact*. <https://doi.org/10.21203/rs.3.rs-3333773/v1>
16. Srivastava, P., Mehta, M., Sagar, R., & Ambekar, A. (2020). Smartteen-a computer-assisted cognitive behavior therapy for Indian adolescents with depression-a pilot study. *Asian Journal of Psychiatry*, *50*, 101970.
17. Superable, G. M. (2022). Guided Self-Help Game-Based Application for Adolescents in the Philippines and Low-to Middle-Income Countries. Report.
18. Wasil, A. R., Park, S. J., Gillespie, S., Shingleton, R., Shinde, S., Natu, S., Weisz, J. R., Hollon, S. D., & Derubeis, R. J.. (2020). Harnessing single-session interventions to improve adolescent mental health and well-being in India: Development, adaptation, and pilot testing of online single-session interventions in Indian secondary schools. *Asian Journal of Psychiatry*, *50*, 101980. <https://doi.org/10.1016/j.ajp.2020.101980>
19. Wasil, A. R., Osborn, T. L., Weisz, J. R., & Derubeis, R. J.. (2021). Online single-session interventions for Kenyan adolescents: study protocol for a comparative effectiveness randomised controlled trial. *General Psychiatry*, *34*(3), e100446. <https://doi.org/10.1136/gpsych-2020-100446>
20. Yatirajula, S. K., Kallakuri, S., Paslawar, S., Mukherjee, A., Bhattacharya, A., Chatterjee, S., Sagar, R., Kumar, A., Lempp, H., Raman, U., Singh, R., Essue, B., Billot, L., Peiris, D., Norton, R., Thornicroft, G., & Maulik, P. K.. (2022). An intervention to reduce stigma and improve management of depression, risk of suicide/self-harm and other significant emotional or medically unexplained complaints among adolescents living in urban slums: protocol for the ARTEMIS project. *Trials*, *23*(1). https://doi.org/10.1186/s13063-022-06539-8
